# Sex differences in electrical activity of the brain during sleep: a systematic review of electroencephalographic findings across the human lifespan

**DOI:** 10.1186/s12938-025-01354-z

**Published:** 2025-03-12

**Authors:** Rhea Chapman, Sarasa Najima, Thaisa Tylinski Sant’Ana, Christy Chi Kiu Lee, Francesco Filice, Jessica Babineau, Tatyana Mollayeva

**Affiliations:** 1 KITE Toronto Rehabilitation Institute, University Health Network, 550 University Avenue 11th Floor, Rm 11-183, Toronto, ON M5G 2A2 Canada; 2 Biology Department, University of Toronto, Mississauga, ON Canada; 3 Arts and Science Department, University of Toronto, Toronto, ON Canada; 4 Library and Information Services, University Health Network, 550 University Avenue, Toronto, ON M5G 2A2 Canada; 5 The Institute for Education Research, University Health Network, Toronto, ON Canada; 6 Dalla Lana School of Public Health, University of Toronto, Toronto, ON Canada; 7 Temetry Faculty of Medicine, Rehabilitation Sciences Institute, University of Toronto, Toronto, ON Canada; 8 Global Brain Health Institute, Trinity College Dublin, University of Dublin, Dublin, Ireland

**Keywords:** Electroencephalogram, EEG, Equity, diversity, and inclusion, Method, Integrated ethics, Health equity, PROGRESS-Plus (Place of residence, Race/ethnicity, Occupation, Gender/Sex, Religion, Education, Socioeconomic status, Social capital; other contextual parameters, including age), Sex differences, Social determinants of health, Inequity, Neurodiversity, Neurobiology, Neuroimaging, Brain, Sleep stages

## Abstract

**Background:**

With the explosion of techniques for recording electrical brain activity, our recognition of neurodiversity has expanded significantly. Yet, uncertainty exists regarding sex differences in electrical activity during sleep and whether these differences, if any, are associated with social parameters. We synthesised existing evidence applying the PROGRESS-Plus framework, which captures social parameters that may influence brain activity and function.

**Methods:**

We searched five databases from inception to December 2024, and included English language peer-reviewed research examining sex differences in electrical activity during sleep in healthy participants. We performed risk of bias assessment following recommended criteria for observational studies. We reported results on sex differences by wave frequency (delta, theta, alpha, sigma, beta, and gamma) and waveforms (spindle and sawtooth), positioning results across age-related developmental stages. We created visualizations of results linking study quality and consideration of PROGRESS-Plus parameters, which facilitated certainty assessment.

**Results:**

Of the 2,783 unique citations identified, 28 studies with a total of 3,374 participants (47% male, age range 4–5 months to 101 years) were included in data synthesis. Evidence of high certainty reported no sex differences in alpha and delta relative power among participants in middle-to-late adulthood. Findings of moderate certainty suggest no sex differences in alpha power; and theta, sigma and beta relative power; and delta density. There is evidence of moderate certainty suggesting that female participants had a steeper delta wave slope and male participants had greater normalized delta power. Evidence that female participants have higher spindle power density is of low certainty. All other findings were regarded as very low in certainty. The PROGRESS-Plus parameters were rarely integrated into the methodology of studies included in this review.

**Conclusion:**

Evidence on the topic of sex differences in sleep wave parameters is variable. It is possible that the reported results reflect unmeasured social parameters, instead of biological sex. Future research on sex differences in sleep should be discussed in relevance to functional or clinical outcomes. Development of uniform testing procedures across research settings is timely. PROSPERO: CRD42022327644. Funding: Canada Research Chairs (Neurological Disorders and Brain Health, CRC-2021-00074); UK Pilot Award for Global Brain Health Leaders (GBHI ALZ UK-23-971123).

**Supplementary Information:**

The online version contains supplementary material available at 10.1186/s12938-025-01354-z.

## Introduction

Sex and gender consideration in neuroscience and health research are widely endorsed by major funding organizations, as there is strong evidence to demonstrate that biological and social factors contribute to differences in health outcomes and are, therefore, relevant for precision medicine and person-centered care [1, 2]. Sex refers to biological characteristics, including differences in chromosomes, reproductive organs, and gonadal hormones. Gender refers to sociocultural characteristics, reflecting the roles, responsibilities, identity, and behaviors of girls, boys, men, women and people of other genders [1, 2]. Both sex and gender are interconnected and influenced by environment and culture.

Several studies reported on sex differences in the structure and function of the brain, during both sleep and wakefulness [4, 5]. Such findings urged scientific journals and regulatory agencies to require researchers to consider biological sex in their research and raised awareness that ignoring sex has ramifications both in terms of rigor and reproducibility of research, potentially leading to costly consequences and unrealized benefit [4, 5]. However, sex interacts with several environmental, developmental, and genetic factors and should, therefore, be investigated in the context of other parameters that influence brain structure and function [6].

Evidence emerged suggesting that social experiences alter neuronal structure and function, and can shape epigenetic influences on central nervous system (CNS) development and decline [7, 8]. This discussion raises an important question regarding the degree to which the observed group-level differences are attributable to biological sex or socio-cultural gender [3], highlighting the need to examine how genetics, environment, and behavior influence neuroplasticity processes across the human lifespan.

Electroencephalography (EEG) is a non-invasive neuroimaging technique that allows researchers to observe dynamic processes in the brain as they happen. It is used to capture electrical activity of the brain during both sleep and wakefulness and allows for the analysis of frequency waves, absolute and relative spectral powers, asymmetry of spectral powers between the right and left hemisphere, intra- and inter-hemispheric magnitude, coherence and phase synchrony, and many other parameters [9]. This technique can be applied to a wide range of scientific inquiries, from the study of cognitive processes in psychology to the study of neural engineering and rehabilitation [10, 11].

Several studies have reported that sex differences exist in EEG frequency waves, in both healthy people and people with a wide range of conditions. For example, a recent study of sex differences in resting EEG in healthy young adults found that females had greater overall amplitudes in delta, alpha, and beta activity during the resting state, enhanced midline activity in theta, and parietal and midline activity in the alpha and beta bands, concluding that these findings indicate significant differences in neuronal activity between young adult female and male persons [12]. In the sleeping state, sex differences were observed in EEG functional connectivity in healthy people [13]. Synchronization intensities showed differences in all sleep stages: higher in female in non-rapid eye movement (NREM) sleep, and higher in male persons in alpha and beta bands in rapid eye movement (REM) sleep [13].

Recent research leveraging deep learning to predict a person’s sex using clinical EEG data of over 1000 people aged 18 to 88 reported that sex was detectable by neural networks with 81% accuracy [14]. Researchers found that the predictive ability was primary driven by EEG topographies, rather than waveforms and frequencies, emphasizing the need for future research considering age, health status, and state in which EEG data is collected (e.g., sleep versus wakefulness) to minimize confounding effects.

In this review, we aimed to synthesize evidence on sex differences in sleep wave parameters in healthy persons, utilizing the PROGRESS-Plus framework (i.e., Place of residence, Race/ethnicity/culture/language, Occupation, Gender/sex, Religion, Education, Socioeconomic status, Social capital, and other characteristics, including age) [15, 16]. We hypothesized that if we observe consistently reported differences in sleep wave parameters between male and female persons during ages that are linked to sex steroid hormone differentiation (i.e., adolescence, menopause) but not during adulthood stages (regardless of the extent of the PROGRESS-Plus consideration), then the difference is more likely driven by biology (i.e., sex), as opposed to social or environmental factors. However, if this hypothesis is not supported by the evidence, then social and environmental factors (i.e., PROGRESS-Plus parameters) are more likely driving the observed differences. We therefore conducted a systematic review to: (1) identify and critically appraise original studies that investigated sex differences in sleep wave parameters in healthy participants using EEG; (2) analyze data by sleep wave parameter and age-related developmental stages; and (3) interpret evidence through the PROGRESS-Plus lens [16].

## Results

### Study selection

**Fig. 1:**
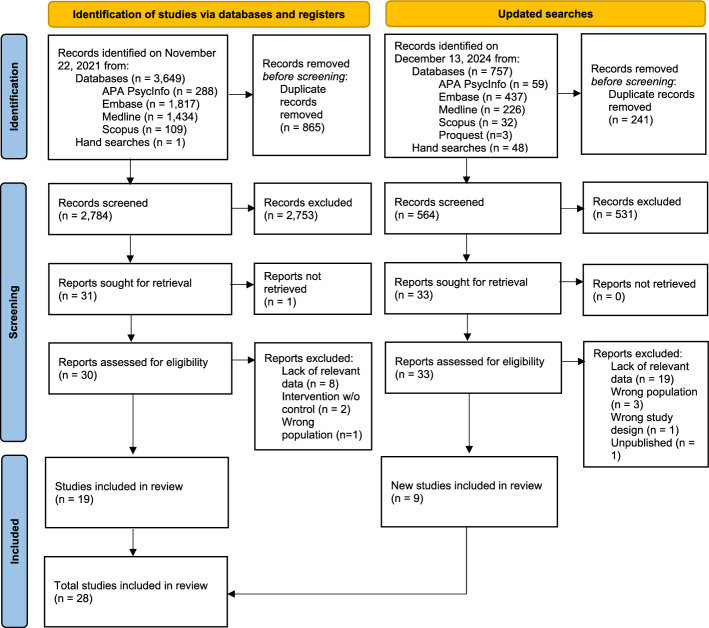
PRISMA flow diagram showing the process of study selection

We identified 3,648 records within the databases searched on November 22, 2021, and 757 records within the repeated databases searched on December 13, 2024. One additional record was located manually [17]. After the removal of duplicates, we screened 2,784 records from the initial searches and 564 records from the repeated searches. We identified 63 citations for full text review, 28 of which met inclusion criteria for data synthesis. Reasons for exclusion for the remaining 35 studies were recorded (Supplementary Material S1) and displayed in the PRISMA flow diagram (Fig. 1). The characteristics of the 28 included studies are reported in Tables 1, 2, and Supplementary Material S2.

**Table 1:** Summary of all included studies with sample characteristics regarding sex differences & PROGRESS-Plus-related results

Author (year); Journal; Country; Region; City; Location of research	(1) Objective (2) Design (3) Follow up/assessment times, if any (4) Inclusion criteria a. Social b. Clinical c. Behavioural d. Other (4) Exclusion criteria a. Social b. Clinical c. Behavioural d. Other	(1) Total sample size, n (M/F) (2) Attrition, % (if multiple assessments) (3) Age, years (mean ± SD) or range (4) Sex, %M (5) Other PROGRESS-Plus parameters reported (6) Additional PROGRESS-Plus parameters considered in analysis of sex diff (7) Developmental stage of participants	(1) Sleep wave (frequency range); technology used [bold indicates waves in which sex differences were investigated] (2) Other sleep measures (3) Statistical analysis/analysis controls for	(1) Sex related results (2) Other PROGRESS-Plus factors related to outcomes (3) Researcher(s) notes
1. Armitage R. (1995); *Sleep;* USA; Texas; Dallas; Sleep laboratory	(1) Analyze EEG parameters & interhemispheric variances by sleep stages & sex (2) Clinical trail (3) Two-time assessment (4) a. NR b. No meds except oral/implant CCs c. Pre-study alc & caff restriction; keep sleep–wake pattern for 5 d d. NR (5) a. NR b. NR c. NR d. First-degree relative w psy d/o	(1) 22 (11 M/11F) (2) NR (3) M (25.6 ± 4.2 years) F (24.8 ± 3.9 years) (4) 50 (5) NR (6) None (7) Emerging & early adulthood	(1) **δ (0.5– < 4 Hz),** **θ (4– < 8 Hz),** **α (8– < 12 Hz),** σ (12– < 16 Hz), β (16– < 32); standalone EEG (2) Sleep efficiency (3) 2-way ANOVA/sex	(1) No sex diff in δ, θ, & α across sleep stages. Overall δ pwr during NREM stages 2–4 was ↑ in F. No sex diff in δ pwr during REM (2) NR (3) Sex diff in EEG during sleep were minimal
2. Armitage R. et al. (2000); *Sleep;* USA; Texas; Dallas; Sleep laboratory	(1) Compare δ activity in NREM btw depressed & healthy participants by sex (2) Comparative study (3) Two-time assessment (4) a. NR b. NR c. 2 continuous d of sleep w/o disruption d. No family Hx of Axis I d/o (5) a. Shift worker b. Current Axis I/sleep d/o c. Substance abuse w/i 12 months pre-study d. NR	(1) 23 (15 M/8F) (2) NR (3) 22–40 years M (27.1 ± 5.9 years) F (30.9 ± 6.2 years) (4) 65 (5) NR (6) Age (7) Emerging & early adulthood	(1) **δ (0.5– < 4 Hz)**; standalone EEG (2) Sleep efficiency, latency (3) R-measures analyses of variance ANOVAs/sex, age, psychiatric status; mean, SD by sex	(1) Normal control F had a ↑ δ power than M, but results were not statistically sign; F showed 4.6% ↑ δ amp but results were not statistically sign (NREM 2–4) (2) Sex diff is likely to ↓ w ↑ age. F showed strong age effect w ↓ pwr & ampl; for M this effect was not stat sign (3) The amount or amp of δ activity showed sign group by sex interactions
3. Baker F. C. et al. (2012); *J Sleep Res;* Australia; Victoria; Abbotsford; Sleep laboratory	(1) Explore regional & sex diff in sleep & EEG changes in adolescents over 6–8 mos (2) Comparative study (3) Two-time assessment (4) a. Adolescents 11–14 y b. NR c. Keep sleep–wake pattern for 5 d d. NR (5) a. NR b. CNS d/o; lifetime psy/neuro/sleep d/o; loss of consciousness > 30 min c. NR d. NR	(1) 33 (18 M/15F) (2) 15 (3) 11–14 years M (12.7 ± 0.9 years) F (12.4 ± 0.7 years) (4) 55 (5) Place of residence, Tanner stage (6) Age (7) Childhood & adolescence	(1) **δ (0.3– < 4 Hz),** **θ (4– < 8 Hz),** **α (8– < 12 Hz),** **σ (12– < 15 Hz),** **β1 (15– < 23 Hz)**; standalone EEG (2) Sleep efficiency, latency (3) Two-way R-measures ANOVAs/sex, age	(1) No sign sex effects were found for θ pwr; no sign sex effects or interaction for δ pwr or amp. No sign effects of age, derivation, sex, or interactions for α, σ, or β pwr (2) Sign main effects of age were observed for δ activity δ pwr & amp ↓ in the occipital region w ↑ age during adolescence (3) θ, α, & σ pwr in NREM & REM sleep at the occipital site ↓ over time, w no sex diff in these age-related changes
4. Campbell I. G. et al. (2005); *Sleep*; USA; California; Davis; Participant’s home	(1) Study the timing of NREM δ EEG pwr decline & pubertal maturation btw sexes (2) Longitudinal study, semi-annual for 4 yrs (3) Two-time assessment (4) a. NR b. NR c. Keep sleep–wake pattern for 5 d/no naps d. NR (5) a. NR b. Hx of head injury/lifetime psy/neuro d/o c. NR d. NR	(1) 70 (35 M/35F) C9: 32 (16 M/16F) C12: 38 (19 M/19F) (2) NR (3) 1st recording mean 9.31 ± 0.04 (C9), 12.33 ± 0.04 (C12); 2nd recording mean 9.83 ± 0.05 (C9), 12.80 ± 0.04 (C12) (4) C9: 50 C12: 50 (5) Tanner stage (6) Age (7) Childhood & adolescence	(1) **δ (0.3–3 Hz),** **3–4 Hz,** **θ (4–6 Hz; 6–8 Hz)** **α (8–12 Hz),** **σ (12–15 Hz),** **β (15–23 Hz, 23–30 Hz),** 30–50 Hz*; standalone EEG (2) Sleep efficiency, density (3) Post hoc t tests/sex, age; correlation coefficients/ delta power density, Tanner stage *Freqs inferred from study Table 4 & text in results section	(1) M in C12 had sign ↑ δ pwr density than F due to a > δ-wave amp. C12 M had ↑ α & β pwr density in the first recording period & ↑ θ, α, σ pwr densities in the second recording period. No sex diff in δ pwr density or amp were observed in C9 (2) Sex diff present in C12 indicates the possibility of F beginning adolescent brain maturation earlier than M. Tanner stage was not correlated w δ activity (3) C12 M had 37% ↑ δ pwr per min than F, while C9 showed no sex diff. Similar results found six months later
5. Campbell I. G. et al. (2012); *PNAS;* USA; California; Davis; Participant’s home	(1) Compare adolescent δ decline in EEG data from 9- & 12-yr-old M & F (2) Longitudinal study, semi-annual for 6 or 7 yrs (3) Two-time assessment (6) a. NR b. NR c. Keep sleep–wake pattern for 5 d d. NR (7) a. NR b. Current psy/neuro d/o c. Sleep disturbance d. NR	(1) 67 (33 M/34F) C9: 30 (15 M/15F) C12: 37 (18 M/19F) (2) 16 (3) 9 years, 12 years (4) 50 (5) Place of residence, race (6) Age, Tanner stage (7) Childhood & adolescence, emerging & early adulthood	(1) **δ (1–4 Hz),** θ (4–8 Hz); standalone EEG (2) Sleep latency, efficiency (3) Gompertz equation/sex, age, Tanner stage	(1) F experienced the most rapid ↓ in δ pwr at 12.53 (± SE 0.19) yrs, 1.21 (± 0.25) yrs earlier than M. Sex diff sign & explaining 32% of the variance. The rate of ↓ was similar btw sexes. No sign diff btw sexes in the upper asymptote of the delta curve or in the change from the upper to lower asymptotes (2) As age ↑ δ pwr ↓, & adolescents peak pubertal maturation was positively correlated w δ pwr decline (3) The rate of ↓ varied sign among participants, w no correlation observed btw the rate of ↓ & other factors. Delayed maturation did not lead to a faster decline
6. Carrier J. et al. (2001); *Psychophysiology;* USA; Pennsylvania; Pittsburgh; Sleep laboratory	(1) Investigating the impact of age & gender on sleep EEG pwr spectral density in middle age participants (2) Cross-sectional study (3) Five-time assessment (4) a. NR b. Healthy c. NR d. No Hx of personal/family psy/neuro/sleep/medical d/o (5) a. NR b. Any medical condition; on sleep meds; obese (BMI > 27)/considerable wt loss; AHI > 10/PLMAI > 10 c. No alc/drugs ≥ 14 d pre-study d. First-degree relative w prev seizures/psy d/o	(1) 100 (53 M/47F) (2) NR (3) 20–60 yrs (4) 53 (5) Occupation (6) Age (7) Emerging & early adulthood, middle & late adulthood	(1) **δ (1–4 Hz),** **θ (NR),** σ (NR), **α (NR),** β (NR) **spindle (NR)**; standalone EEG (2) Sleep density (3) Multivariate modeling/sex, age	(1) F had ↑ spectral pwr density in δ, θ, low α bins in NREM1 (0.25 -9.00 Hz), NREM2 (0.25–10.00 Hz), NREM3 (0.25–9.00 Hz), NREM4 (0.25–10.00 Hz). F had ↑ spindle pwr density from 14.25 to 16.00 Hz in all four NREM periods compared to M (2) ↑ age is associated w fragile sleep wake system manifested through ↓ pwr spectral density in SW sleep & ↑ in high freq power (3) No sign interactions were found btw age & sex, suggesting that the aging process does not differently affect M & F btw 20 & 60 yrs old
7. Carrier J. et al. (2011); *Eur J Neurosci;* Canada; Quebec; Montreal; Sleep laboratory	(1) Study how age, sex, & topography impact SW characteristics in young & middle-aged participants (2) Longitudinal study (3) Multiple assessment (4) a. 2 age groups: 20–30 & 41–60 b. < 4 on BDS c. NR d. Had PSG screen (5) a. Night work b. On sleep altering meds; psy/neuro d/o Hx c. Smoking; Transmeridian travel 3 mos pre-study; sleep disturbance/sleep duration of ≤ 7/ ≥ 9 h; perimenopausal d. F’s using hormonal CCs/HRT	(1) 87 (44 M/43F) a. Young: 48 (26 M/22F) b. Middle-aged: 39 (18 M/21F) (2) NR (3) Young: 20–30 years (23.3 ± 2.4 years) Middle-aged: 41–60 years (61.9 ± 4.6) (4) Young: 54 Middle-aged: 46 (5) NR (6) Age (7) Emerging & early adulthood, middle & late adulthood	(1) **δ (1–4 Hz)**; standalone EEG (2) Sleep efficiency, latency (3) Two-way ANOVA/sex, age	(1) F exhibited ↑ SW amp & slope compared to M (2) ↑ in age is correlated w ↓ SW amp & slope (3) Compared to M, F showed ↑ SW amp, shorter SW freq, shorter positive phase duration. Older adults also showed slower SW density & amp
8. Dijk D. J. et al. (1989); *Sleep;* Netherlands; Groningen Province; Groningen; Sleep laboratory	(1) Identify sex diff by comparing sleep in young adult & published sleep deprivation EEG data (2) Cross-sectional study (3) Two-time assessment (4) a. NR b. NR c. No sleep complaints d. NR (5) a. NR b. NR c. NR d. NR	(1) 28 (13 M/15F) (2) NR (3) 19–27 years M (23.5 years) F (21.9 years) (4) 46 (5) NR (6) None (7) Emerging & early adulthood	(1) **EEG freq (0.25–15 Hz)**; standalone EEG (2) Sleep latency (3) Student’s t test/sex	(1) In NREM sleep, F had ↑ pwr densities in δ freq compared to M, except in the 12 & 13 Hz bins. In REM sleep, F showed ↑ pwr densities in all freq bins except the 9 & 10 Hz bins (2) Sex diff may not be associated w differential aging process due to its presence in participants in their 20s (3) Published data on sleep deprivation effects on EEG pwr spectra didn't indicate a common mechanism for sleep deprivation effects & sex diff in sleep EEGs
9. Dorokhov B. V. et al. (2024); *Chronobiol. Int.*; Russia; Moscow; Sleep laboratory	(1) Understand the paradoxical relationship between sex & objective & subjective sleep quality using EEG during nap (2) Cross-sectional (3) Three-time assessment (4) a. NR b. NR c. NR d. NR (5) a. ≤ 18 & > 23 yrs; shift worker b. Hx of mental/sleep d/o; current mild cold c. Complaints about poor phys condition & functioning; missed class in the 2 wks pre-study; crossed time zones 1 month pre-study; irregular sleep–wake schedule (1 + h diff in wkday bedtime, frequent sleep reduction) d. Pregnant/breastfeeding	(1) 80 (40 M/40F) (2) NR (3) M (20.4 ± 1.57 years) F (20.25 ± 1.14 years) (4) 50 (5) Education (6) None (7) Early adulthood	(1) **δ (1–4 Hz),** **θ (5–8 Hz),** **α (9–12 Hz),** **σ (13–16 Hz)**; standalone EEG (2) Spectral EEG markers of sleep & wake drives, sleep stage durations (3) 1st & 2nd PCA EEG spectrum, Pearson r, rANOVA/sex	(1) F had ↑ spectral pwr density across all sleep stages for δ, θ, α, σ (2) NR (3) F's stronger sleep drives linked to ↑ δ activity; sleep complaints may vary
10. Feinberg I. et al. (2006); *Am J Physiol Regul Interg Comp Physiol;* USA; California; Davis; Participant’s home	(1) Investigate the relationship btw δ decline, puberty, growth, sleep patterns, sex & age (2) Controlled clinical trial (3) Two-time assessment (4) a. NR b. No psy/neuro/medical d/o; on meds affecting CNS c. No sleep complaints; keep sleep–wake pattern for 5 d/no naps d. NR (5) a. NR b. NR c. NR d. First-degree relative w major psy d/o	(1) 69 (34 M/35F) C9: 31 (15 M/16F) C12: 38 (19 M/19F) (2) NR (3) 9 years, 12 years C9: (9 years ± 3 mos) C12: (12 years ± 3 mos) (4) C9: 48 C12: 50 (5) Place of residence, race, Tanner stage (6) Age, Tanner stage (7) Childhood & adolescence	(1) **δ (0.3–3 Hz)**, 1–4 Hz; standalone EEG (2) Sleep efficiency (3) ANOVA/sex, age, recording session; mixed effect analyses/ δ pwr density, age, height, weight, BMI, Tanner stage	(1) NREM δ pwr density did not alter from ages 9–11, w no sex diff. It ↓ by 25% btw 12 & 14, w F showing lower levels, indicating an earlier decline (2) The ↓ in δ pwr density is strongly linked to both age & sexual maturation stage. Tanner stage, height, weight, or BMI was unrelated to δ pwr density once the age effect was removed (3) In C9, there were no sign effects of recording session or sex on δ pwr density, & no interaction btw recording & sex. In C12, δ pwr density ↓ across four recordings & was sign ↓ in F than M, though the rate of ↓ was similar btw sexes from ages 12 to 14
11. Fukuda N. et al. (1999); *Psychiatry Clin Neurosci;* Japan; Hokkaido; Sapporo; Participant’s homes	(1) Explore sex effects on SWA in middle aged & elderly participants by spectral analysis (2) Cross-sectional study (3) Three-time assessment (4) a. NR b. No sign medical; No meds c. NR d. NR (5) a. NR b. NR c. NR d. NR	(1) 16 (8 M/8F) (2) NR (3) 54–72 years M (61.5 ± 4.66 years) F (62.38 ± 6.65 years) (4) 50 (5) NR (6) None (7) Middle & late adulthood	(1) **δ (0.5–4 Hz)**; standalone EEG (2) Sleep efficiency, latency (3) Mann–Whitney U test/sex	(1) F have longer SWS durations & ↑ rate of SWA than M. The total amount of δ band spectral pwr is ˃ in F compared to M. F exhibit clearer periodic fluctuations in δ band pwr throughout the night (2) Middle aged & elderly F showed a sign ↑ pwr level for both freq δ bands (3) Sign sex diff exist in SWS, w middle-aged & elderly F showing a more conservative SWS-generating mechanism compared to M
12. Hejazi S. N. et al. (2024); *J Psychiatr Res*; USA; Bethesda; NIH Clinical Centre	(1) Examine relationship between sex, age, & sleep patterns w low & high δ pwr (2) Randomized control trial (3) Two-time assessments (4) a. 18–65 yrs b. No psychotropic meds for 2 + wks (5 + for fluoxetine & 3 + for aripiprazole); absence of Axis I d/o c. NR d. No first-degree relative w DMS-IV Axis I d/o (5) a. NR b. NR c. NR d. NR	(1) 24 (8 M/16F) (2) NR (3) 20–56 years (33.75 ± 11.02 years) (4) 33 (5) NR (6) Age (7) Early & middle adulthood	(1) **Low δ (0.5–2 Hz),** **high δ (2–4 Hz)**; standalone EEG (2) TST, sleep efficiency, WASO (3) Mann–Whitney U test/sex	(1) No sign sex diff in **δ** spectral pwr density btw healthy M & F (2) NR for healthy participants (3) NR for healthy participants
13. Kluge M. et al. (2010); *PNEC;* Germany; Bavaria; Munich; Sleep laboratory	(1) Study sex diff in ghrelin’s impact on sleep & the secretion of GH & cortisol in elderly participants (2) RCT (PL-controlled) (3) One-time assessment (4) a. NR b. Healthy c. No caff, alc, naps d. NR (5) a. NR b. Any sleep disturbance/d/o; Any meds/HRT c. Sleep duration of ≤ 6 h/ ≥ 9 h/ transmeridian flight 3 mos pre-study d. Depressive symptoms	(1) 20 (10 M/10F) (2) NA (3) 60–70 years M (64 ± 2.2 years) F (63 ± 2.9 years) (4) 50 (5) NR (6) None (7) Middle & late adulthood	(1) **δ (0.5–4 Hz),** **θ (4.5–8 Hz),** **α (8.5–12 Hz),** **σ (12.5–16 Hz),** **β (16–20 Hz)**; standalone EEG (2) Sleep density, efficiency (3) Mean, SD by sex	(1) PL F had a ↑ pwr than M for δ; PL M had ↑ pwr for β. No diff in θ, α, or σ pwr (2) GH affects sleep in elderly M but not F resembling findings in young participants (3) In M, δ pwr sign ↑, while α & β pwr ↓ after GH compared to PL injection during the first half of the night. In both sexes, ghrelin caused comparable ↑, secretion patterns of GH & cortisol. GH caused a sign ↑ of stage 2, SWS, NREM, while ↓ of stage 1 & REM in elderly M. GH did not affect sleep in postmenopausal elderly F
14. Latta F. et al. (2005); *Sleep;* USA; Illinois; Chicago; Sleep laboratory	(1) Examine sex effects on sleep stages & EEG spectral pwr in older participants (2) Clinical trial (3) Two-time assessment (4) a. NR b. No obese participants; normal lab test; no Hx of psy/sleep/neuro/endocrine d/o; no meds/HRT c. Non-smoking; < 5 on GDS; > 25 on FMSE d. F 1 + y past menopause w no hot flash (5) a. Shift workers b. Diabetes/glucose intolerance c. 2 + caff drinks per d; shift workers/transmeridian travel 4 weeks pre-study d. NR	(1) 20 (10 M/10F) (2) 10 (3) M (59 ± 2 years) F (63 ± 2 years) (4) 50 (5) Place of residence (6) None (7) Middle & late adulthood	(1) **δ (0.5–4 Hz),** **α (8.5–12 Hz)**; standalone EEG (2) Sleep latency, sleep period, TST, sleep maintenance (3) 2-way ANOVA/sex	(1) Absolute δ & α activity was ↑ in F than M, irrespective of sleep stage. No sex diff in relative δ or α activity in either NREM or REM (2) As age ↑ there is an ↑ age related changes in sleep architecture that occur & differ btw sexes (3) Older F show ↓ δ activity than M. The sex difference in δ activity is more pronounced in REM than NREM sleep, w stable REM δ activity throughout the night in both sexes. When normalized for REM, F have ↓ NREM δ activity than M. Blood sampling ↓ total sleep time, sleep maintenance, SWS, & δ activity more in F. In F, but not M, blood sampling ↓ REM δ activity, which strongly correlates w NREM δ activity
15. Luo X. et al. (2024); *Front Psychol*; NR; Sleep laboratory	(1) To investigate the relationship between age, gender, & EEG functional connectivity (2) Retrospective study (3) Two-time assessment (4) a. NR b. No sleep-related meds c. NR d. NR (5) a. NR b. NR c. NR d. Incomplete data	(1) 135 (56 M/79F) (2) 13 (3) 25–101 M (57.89 ± 21.34 years) F (54.39 ± 21.65 years) (4) 41 (5) NR (6) Age (7) Early, middle & late adulthood	(1) **δ (0.5–4 Hz),** **θ (4–8 Hz),** **α (8–13 Hz),** **β (13–30 Hz),** **Spdl (11–16 Hz),** **Sawtooth (3–7 Hz);** standalone EEG (2) Sleep staging (3) Machine learning models (SVM, RF, KNN)/sex, age; one-way ANOVA/sex, age	(1) F had ↑ FC in α, β, & spdl bands across sleep stages; no sex diff in δ, θ, & sawtooth connectivity across sleep stages (2) Diff in functional connectivity was seen btw diff age groups (3) Enhanced β & spdl connectivity may support better memory consolidation in F
16. Ma J. et al. (2011); *J Clin Sleep Med;* USA; NR; Sleep laboratory	(1) Study gaboxadol’s effect on NREM sleep EEG patterns in transient insomnia using pwr spectral analysis (2) RCT (PL-controlled) (3) One-time assessment (4) a. 18–64 years b. NR c. NR d. NR (5) a. NR b. Sleep/psy d/o; > 5 on PSQI/ > 12 on ESS; sleep apnea/PLMD; sleep duration 6.5–9 h; bedtime btw 21:00–24:00 ≥ 4 times a wk; latency < 30 min c. MSLT < 10 min; excessively sleepy participants d. NR	(1) 822 (314 M/508F) (2) NA (3) 18–64 years M (28.78 ± 8.21 years) F (31.76 ± 10.70 years) (4) 38 (5) NR (6) None (7) Emerging & early adulthood, middle & late adulthood	(1) **δ (0.5–4.25 Hz),** **θ (4.25–8 Hz),** **α (8–12 Hz),** **σ (12–15 Hz),** **β (15– 32.5 Hz),** **Spdl (11–14 Hz);** EEG via PSG (2) Sleep latency, efficiency (3) Linear mixed-effects model/sex, frequency, & their interactions	(1) At baseline, F showed sign ↑ power spectral densities in low-freq (1–10 Hz) & high spdl/low β freq (14–18 Hz) ranges. High β (19–32 Hz) power spectral densities were > in F but the diff was not statistically sign compared to M. From the figure, sigma power spectral densities were > in F from 14–15 Hz but no sign sex differences from 12 to < 14 (2) NR for placebo prior to intervention (3) NR for placebo prior to intervention
17. Markovic A. et al. (2020); *Sci Rep;* Switzerland; Bern; Bern; Participant’s homes	(1) Examine sex diff in sleep EEG power & coherence across freq bands in NREM & REM in adolescents (2) Clinical Trial (3) Two-time assessment (4) a. 9–14 years b. NR c. NR d. Born after 30th week of pregnancy (5) a. NR b. NR c. NR d. Poor quality EEG data	(1) 61 (30 M, 31F) (2) NR (3) 9–14 years M (12.83 ± 0.75) F (12.12 ± 1.67) (4) 49 (5) Tanner stage (6) Age, Tanner stage (7) Childhood & adolescence	(1) **δ (1–4.6 Hz),** **θ (4.8–7.8 Hz),** **α (8–10.8 Hz),** **σ (11–16 Hz),** **β1 (16.2– 20 Hz),** **β2 (20.2– 24 Hz),** **γ1 (24.2–34 Hz),** **γ2 (34.2–44 Hz),** **Spdl (10–16 Hz)**; **Slow spindle (10–12 Hz); Fast spindle (12–16 Hz)**; standalone EEG (2) Sleep quality, latency, time, efficiency (3) ANOVA/sex, age, Tanner stage, relatedness	(1) No diff in absolute EEG pwr btw M & F in the lower freq bands (< 11 Hz) during NREM & REM sleep. F showed sign ↑ pwr in the σ band during NREM sleep & ↑ absolute pwr in higher freq (β1 to γ2) across NREM & REM sleep. When normalized, most pronounced sex diff were in NREM δ pwr. High-freq brain activity (16.2–44 Hz) was ↑ in F. F had sign ↑spdl amp & density. In analysis of F with no menarche & age-matched M, same results were observed for slow spdl; for fast spdl no sex diff in density F showed > coherence than M across all freq bands in both NREM & REM, except in the α band where M had > values in occipital & temporal regions during NREM, & no sign sex diff during REM. The most pronounced sex diff were in δ during NREM & REM sleep, & in σ during NREM. Pwr decline occurred at a later age & pubertal maturation stage in M than F (2) Sign age effects in EEG pwr in NREM & REM in δ band, w shift twd frontal regions as age ↑; α, σ, γ1, & γ2 bands (in NREM) shifted twd posterior regions w age. Older age linked to shorter slow spdl duration, ↓ spdl amp, & ↑ spdl freq. Age affected coherence across bands & states, with some connections ↓ but an overall ↑ in coherence with age (3) Oscillatory activity across freq bands & sleep states more coherent in F than M, suggesting greater connectivity in F, except for α band. It is important taking sex into account when designing & interpreting studies of sleep neurophysiology
18. Mongrain V. et al. (2005); *Sleep;* Canada; Quebec; Montreal; Sleep laboratory	(1) Explore relationship of chronotype on sleep stages & quantitative sleep EEG btw sexes (2) Clinical trial (3) Two-time assessment (4) a. No shift work b. Healthy c. Non-smoker; > 85% sleep efficiency; latency < 30 min; AHI + PLMI < 5 per h; MSLT > 7 min; No drugs; no transmeridian travel 3 mos pre-study d. NR (5) a. NR b. NR c. Bedtime btw 7–9 h; 3 + alc drink/2 + caff drink per d d. NR	(1) 24 (12 M/12F) Morning: 12 (6 M/6F) Evening: 12 (6 M/6F) (2) NR (3) 19–34 years; Morning: (24.7 ± 1.5 years) Evening: (23.4 ± 0.7 years) (4) 50 (5) Language, occupation, social capital (6) None (7) Emerging & early adulthood	(1) **δ (0.75–4 Hz),** **θ (4–8 Hz),** α (8–12 Hz), low σ (12–14 Hz), **high σ (14–16 Hz),** β (16–24 Hz); EEG via PSG (2) Sleep quality, sleep regulation, sleep regulation (3) 2-way ANOVA/sex	(1) F had ↑ spectral pwr in δ, θ & high σ compared to M (2) Younger adult's regulation differs btw diff chronotypes (3) Sleep-stage analysis showed no diff in sleep architecture btw morning & evening types. Morning-type M had > stage 1 sleep & ↓ sleep efficiency than evening-type M. Morning types had > spectral pwr in low σ during NREM sleep, while the biggest diff btw sexes was in high σ. The decay rate of SWA was faster in morning types but similar btw F & M
19. Mourtazaev et al. (1995); *Sleep*; Netherlands; Leiden; Participant’s homes	(1) Establish how age & sex affect NREM EEG; whether neuronal SW-generating mechanism is involved (2) Cross-sectional (3) One-time assessment (48-h recording; two 10-h periods starting “light off” analysed) (4) a. NR b. Healthy; no somatic/neuro/psy d/o; no sleep-related meds c. No sleep complaints d. NR (5) a. NR b. NR c. NR d. NR	(1) 59 (27 M/32F) (2) NA (3) 26–101 years (4) 46 (5) NR (6) Age (7) Young (26–35), middle (51–60), late adulthood (66–75, 85–101)	(1) **SW (0.5–2 Hz)**; EEG via PSG (2) SW duration, SW%, max, total (3) ANOVA/sex, age, & their interaction; Student–Newman–Keuls/age; mean, SD by sex & age group	(1) Total SWP was sign > in F than M (2) There was a ↓ in all sleep parameters with ↑ age (3) Sex has direct effect on EEG amp & does not affect internal generation of SW in EEG; may be caused by sex-related anatomical diff such as head size, thickness of skull, brain anatomic orientation, cell density & anatomy, electrochemical capacity of cells, etc
20. Pun M. et al. (2023); *J Sleep Res*; Canada; Alberta; Calgary; Participant’s home	(1) Examine relationship btw sleep spdl characteristics & cognitive function in older adults (2) Cross-sectional (3) One-time assessment (4) a. NR e. BMI < 35 kg m^−2^; no cog decline (MoCA ≥ 24) f. Non-smoker for 1 + years; sedentary (< 30 min of exercise 4d/week) g. NR (5) a. NR e. NR f. NR g. NR	(1) 21 (7 M/14F) (2) NA (3) 51–80 years M (66.86 ± 8.6 years) F (65.9 ± 5.0 years) (4) 67 (5) Education (6) None (7) Middle & late adulthood	(1) **Spdl density (11–16 Hz)**; EEG via PSG (2) TST, sleep efficiency, latency, REM, time in each sleep stage, # of awakenings, sleep spdl characteristics, cognitive findings, # of spdls in NREM 2,3 (3) One-way ANOVA, Bonferroni-corrected pairwise comparisons/OSA, sex; mean, SD for no OSA by sex	(1) No sex difference in spdl density in frontal & central electrodes (calculated by SR authors using mean & SD data) (2) Middle aged participants have ↓spdl density is associated w poor cognitive performance (3) Fast spdls linked to better cog performance in F but weaker in M w/ OSA
21. Ringli M. et al. (2013); *Int J Psychophysiol;* Switzerland; Zurich; Zurich; Sleep laboratory	(1) To examine whether sexually dimorphic features are also reflected in topography of sleep SWA (2) Cross-sectional (3) Two-time assessment (4) a. Right-handed b. No psychopathology, chronic medical diseases, sleep complaints/primary sleep d/o c. Non-smoker d. NR (5) a. NR b. On psychoactive agent/meds c. > 4 cups of coffee/same amount of caff per d.; ≤ 1 glass of alc e. NR	(1) 22 (11 M/11F) (2) NR (3) M 8.7–19.4 years (13.4 ± 3.9 years) F 9.1–19.0 years (13.4 ± 3.9 years) (4) 50 (5) Tanner stage (6) Age (7) Childhood & adolescence	(1) **SW (0.75–4.5 Hz)**; standalone EEG (2) Sleep efficiency, latency (3) ANOVA/sex, age	(1) F showed a sign ↑ in SWA pwr in the right & left temporal region compared to M. M had sign ↑ SWA in the right frontal region than F (2) No sign effects of age (3) In F, SWA during the first 60 min of NREM sleep was ↑ over bilateral cortical areas, while in M it was ↑ over the right prefrontal cortex. Sleep variables showed no sign sex diff except for a ↑ percentage of SWS & > absolute SWA in F
22. Rosinvil T. et al. (2021); *SleepJ*; Canada; Quebec; Montreal; Sleep laboratory	(1) Investigate a data-driven approach to identifying age & sex differences in SW (2) Cohort study (3) One-time assessment (4) a. NR b. No drugs; no symptoms of depression/anxiety; AHI < 10; PLMI < 10 c. NR d. Premenopausal women had regular cycle in last yr; menopausal women had no cycle for 1 + years & no vasomotor complaints (5) a. Night worker b. On sleep-affecting meds c. Smoker; self-reported sleep complaints; unusual sleep duration (< 7 h/ > 9 h) d. Transmeridian travel 3 mos pre-study e. Perimenopausal F	(1) 284 (132 M/152F) C1–younger: 97 (45 M/52F) C1–older: 110 (49 M/61F) C2–younger: 38 (19 M/19F) C2–older: 39 (19 M/20F) (2) NA (3) 20–71 C1–younger (23.8 ± 2.8 years) C1–older (57.5 ± 5.1 years) C2–younger (22.7 ± 2.4 years) C2–older (59.6 ± 5.4 years) (4) Sex%M (5) NR (6) Age (7) Emerging, early, middle, & late adulthood	(1) **SW (PtP ≥ 75 μV & NegA ≥ 40 μV, negative to positive deflection ≥ 125 to ≤ 1,500 ≤ 1,000 ms)**; EEG via PSG (2) Standard & adapted criteria, SNR (3) Two-way ANOVA/sex, age; adaptation for amp detection/sex, age	(1) F had ↑ SW density, amp, steeper slopes w/ standard criteria. Age- & sex- adapted criteria removed sex diff in SW density, but F maintained ↑ amp & steeper slopes (2) Older M showed the greatest decline in SW amp & slope compared to older F; older M produced higher proportion of low-amp SW (< 80 µV) (3) ↑ SW amp in F may result from thinner skulls. Adapted criteria minimized detection bias, showing consistent age-related ↓ in SW gen., esp. in older M
23. Ujma P. P. et al. (2019); *Neurobiol Aging;* Hungary/Germany; Central Hungary/ Bavaria; Budapest/ Munich; Sleep laboratory	(1) Investigate age & sex effects on sleep EEG functional connectivity in adults (2) Quantitative study (3) Two-time assessment (4) a. NR b. No drugs except CC; no neuro/psy d/o c. < 2 cups of caff before noon d. NR (5) a. NR b. NR c. NR d. NR e. Poor EEG data	(1) 172 (94 M/78F) (2) NR (3) 17–69 years (29.74 ± 10.71 years) (4) 55 (5) NR (6) Age (7) Childhood & adolescence, emerging & early adulthood, middle & late adulthood	(1) δ (1–3 Hz), θ (3.25–7.75 Hz), **α (8–10.75 Hz),** **σ (11–15.75 Hz),** **β (16–25 Hz)** **γ (25.25–48 Hz)** frequencies; EEG via PSG (2) Sleep efficiency (3) Welch t-test/sex, age, general intelligence	(1) Connectivity was ↑ in F than in M in NREM σ freq range (13.5–14.5 Hz). M had ↑ connectivity in NREM & REM β ranges, (15.75–27.25 Hz) & (12.25–24.75 Hz), respectively, & in NREM high α /low σ (10.25–11.75 Hz), and in **γ** (2) Connectivity in θ (NREM 4.25–5.5 Hz, REM 2.5–5.25 Hz) & β (NREM 30.75–40 Hz, REM 26.75–40 Hz) ranges ↑ w age. Connectivity ↓ in NREM σ range (10.25–14.75 Hz) (3) F had sign > connectivity in the high σ freq range compared to M, w the opposite for the α / σ sigma & β ranges. Connectivity was not sign linked to general intelligence in either sex. Sleep spdl-freq activity loses synchrony w aging & found that high β pwr ↑ w age
24. Ujma P.P. et al. (2022); *Sci Rep*; Germany/Hungary; Munich/Budapest; Sleep laboratory	(1) Study EEG envelope spectrum through comparing EEG of epileptic patient & healthy participants (2) Retrospective study (3) Multiple time assessment (4) a. NR b. Healthy; no Hx of neuro/psy d/o; no drugs except CC c. > 2 cups of coffee before noon; no alc d. NR (5) a. NR b. NR c. NR d. NR	(1) 176 (95 M/81F) (2) NR (3) 17–69 years (29.8 ± 10.66 years) (4) 54 (5) NR (6) Age (7) Childhood & adolescence, emerging & early adulthood, middle & late adulthood	(1) **Low δ** (0.5–2 Hz), **high δ** (2–4 Hz), θ (4–7 Hz), α (7–10 Hz) **low σ (10–12.5 Hz)** **high σ (12.5–16 Hz),** **β (16–30 Hz),** γ (30–49 Hz); standalone EEG (2) Coupling btw different brain oscillations (3) Elastic net regression using MATLAB lasso () function multivariate analyses/sex, age, IQ	(1) M ↓ amplitude of β pwr oscillation at < 0.75 Hz & > 1.75 Hz; M ↑ amplitude at ~ 1 Hz irrespective of sleep state M ↓ amplitude of NREM low **σ** pwr oscillation at ~ 0.05–0.1 & ~ 0.5–1.5 Hz; M ↑ amplitude at ~ 0.25–0.5 Hz & > 1.75 Hz (2) Older age linked to ↓low-freq & ↑ high-freq oscillations in NREM. In REM, age linked to a trend for ↑ low- & high-freq pwr oscillations & ↓ at ~ 0.5–1 Hz (3) NREM envelope spectrum was a highly reliable individual marker strongly associated w ageing & partially sexually dimorphic
25. Ventura S. et al. (2022); *SleepJ*; Ireland; Cork; Sleep laboratory	(1) Investigate influences of sex on macrosleep structure & sleep spdls for infants of 4–5 mos (2) Randomized control study (3) One-time assessment (4) a. NR b. Healthy c. NR d. Born after 37 wks of GA; singleton (5) a. NR b. NR c. NR d. NR	(1) 91 (54 M/37F) (2) NA (3) 4–5 mos (39.8 weaks ± 1.2 weaks) (4) 59 (5) Parent’s ethnicity, PNA, PMA (6) Age (GA, PMA, PNA) (7) Infancy & tolddlerhood	(1) **Spdl (~ 11–15 Hz)**; standalone EEG (2) Normative values for sleep macrostructure at 4–5 mos of age, brain symmetry index (3) Univariable & multivariable linear regression analyses/sex, age (GA, PNA, PMA), timing of nap	(1) M had ↓ spdl pwr (25.4% & 24.5% less, in univariate & multivariate regression, respectively); no sex diff in spdl density (2) Neither GA, PNA, nor PMA were statistically sign in models for spdl pwr, freq, & density (3) Diff at young age may be driven by diff hormonal & brain connectivity profiles
26. Yoon J. et al. (2021); *J Sleep Res;* Korea; Gyeonggi; Ansan; Participant’s homes	(1) Identify role of age & sex in sleep structure w EEG spectral pwr analyses of middle-aged/older participants (2) Cohort study (3) Two-time assessment (4) a. NR b. NR c. NR d. Underwent PSG protocol in the 6th biennial exam btw 2011–2012 (5) a. NR b. Major neuro/psy d/o c. Substance abuse d. Poor quality EEG	(1) 644 (330 M/314F) (2) NR (3) 45–69 years M (57.7 ± 6.5yrs) F (57.9 ± 6.9 years) (4) 53 (5) Place of residence, ethnicity (6) Age (7) Middle & late adulthood mourt	(1) **δ (0.75–4 Hz),** **θ (4–8 Hz),** **α (8–12 Hz),** **σ (12–14 Hz),** **β (14–30 Hz)**; EEG via PSG (2) Sleep efficiency (3) Student’s t-test/sex; ANOVA/age; multivariable linear regression analyses of pwr /sex, age, AHI, BMI, TST	(1) Non-OSA M had ↑ β relative spectral pwr during the whole night, especially N1 sleep; M had ↑ σ relative pwr during N1. M had ↓ θ relative pwr during REM M had ↓ absolute spectral pwr in all frequency bands during the whole night of sleep, REM, & NREM (2) Relative δ pwr ↓, whereas θ, α & σ pwr ↑ during whole night w ageing. Effect of age & sex on relative spectral pwr varied throughout whole night, NREM & REM, & specific sleep stages (3) Age & sex-related diff in sleep pwr were similar between OSA & non-OSA individuals
27. Yuksel D. et al. (2021); *Sleep Health*; USA; California; Menlo Park; Sleep laboratory	(1) Study sex & age diff in stress related sleep disturbances (2) Multicenter study (3) Two-time assessment (4) a. NR b. Free of major mental & phy condition; no insomnia/other sleep d/o c. Keep sleep–wake cycle for 5d d. NR (5) a. NR b. NR c. NR d. NR	(1) 106 (57 M/49F) (2) NR (3) 12.1–19.9 M (15 ± 1.9 years) F (15.3 ± 2 years) (4) 54 (5) Place of residence, ethnicity, pubertal development (6) None (7) Adolescence	(1) **δ (0.3–4 Hz),** **θ (> 4–8 Hz),** **α (> 8–12 Hz)** **σ (> 12–15 Hz),** **low β (> 15–23 Hz),** **high β (> 23–30 Hz)**; standalone EEG (2) Macro/microstructure, sleep efficiency, WASO, NREM/REM, HR, ANS HR variability; night effect (3) Mean, SD by sex	(1) No sign diff between sexes in relative power for all freq bands (calculated by SR authors using mean & SD data) (2) NR (3) Sex-based stress reactivity in sleep may contribute to ↑ insomnia rates in F
28. Zhang Y. Z. et al. (2021); *J Neurosci*; USA; California; Davis; Sleep laboratory	(1) Compile a dataset of longitudinal measurements of spdl microstructure in adolescence (2) Longitudinal (3) Multiple time assessment (4) a. NR b. NR c. NR d. NR (5) a. NR b. NR c. NR d. NR	(1) 98 (51 M/47F) C6: 28 (17 M/11F) C9: 32 (15 M/17F) C12: 38 (19 M/19F) (2) NR (3) 5.96–18.4 years (4) 52 C6: 61 C9: 47 C12: 50 (5) NR (6) Age (7) Early, middle & late childhood & adolescence	(1) **Spdl (12–15 Hz)**; standalone EEG (2) Central spdl change w age (freq, amp, density) (3) Mixed-effects models/sex, age^2^, age^3^	(1) For central spdl amp, M had sign larger ↓ & sign later age for most rapid ↓ (1.4 yrs later for M compared to F); for frontal spdl amp, the age of most rapid ↓ was sign later in M than F (1.5 yrs later in M). No sex diff in spdl density peak for central & frontal electrodes (2) Spdl freq ↑ linearly with age; density peaked at 15 yrs; amp most rapid ↓ at 15 (3) Spdl amp ↓ is suggestive of synaptic pruning, which occurs earlier in F

In this table we have used the terms ‘sex’, ‘male’ & ‘female’ when researchers reported results based on biological attributes of their participants, regardless of the term used in the original text

*AHI* apnea–hypopnea index, *alc* alcohol, *amp* amplitude, *(r)ANOVA* (repeated) analysis of variance, *ANS* autonomic nervous system, *BDS* Beck Depression Scale, *BMI* body mass index, *btw* between, *C* cohort, *caff* caffeine, *cog* cognitive, *CC* contraceptive, *CI* confidence interval, *CNS* central nervous system, *CS* cross-sectional, *d* days, *diff* difference(s), *DMS-IV* Diagnostic & Statistical Manual of Mental Disorders, Fourth Edition, *d/o* disorder, *ECG* electrocardiogram, *EEG* electroencephalogram, *ESS* Epworth Sleepiness Scale, *F* female(s), *FC* functional connectivity, *FMSE* Folstein Mini-Mental Status Examination, *freq* frequency, *f/u* follow up, *G* group, *GA* gestational age, *GDS* Geriatric Depression Scale Short Form, *GH* growth hormone, *GM* grey matter, *Hx* history, *HR* heart rate, *HRT* hormone replacement therapy, *L* longitudinal, *LASSO* least absolute shrinkage & selection operator, *ln-trans* ln-transformed, *KNN* K-Nearest Neighbor, *MANOVA* Multivariate Analysis of Variance, *M* male, *Meds* medications, *MI* mutual information, *Min* minute(s), *ML* machine learning, *mos* months, *MSLT* Multiple Sleep Latency Test, *NA* not applicable, *NegA* negative peak amplitude, *neuro* neurological, *NR* not reported, *NREM* non-rapid eye movement, *OSA* obstructive sleep apnea, *PC* principal component, *PDS* Pubertal Developmental Scale, *phys* physical, *PL* placebo, *PLMAI* Periodic Limb Movements Arousal Index, *PLMD* Periodic Limb Movement Disorder, *PLMI* Periodic Limb Movements Index, *PMA* postmenstrual age, *PNA* postnatal age, *PtP* peak-to-peak, *PSD* power spectral density, *PSG* polysomnography, *Psy* psychiatric, *PSQI* Pittsburgh Sleep Quality Index, *pwr* power, *RCT* randomized controlled trial, *REM* rapid eye movement, *RF* random forest, *sign.* Significant, *spdl(s)* spindle(s), *SR* systematic review, *SW* slow-wave, *SWA* slow-wave activity, *SWS* slow-wave sleep, *SVM* support vector machine, *TST* total sleep time, *USA* United States of America, *w* w, *WASO* wake after sleep onset, *w/i* within, *wk* week, *wt* weight, *w/o* without, *yr(s)* year(s)

↓, decreased; ↑, increased; < , less; > , greater/more; β, beta; α, alpha; δ, delta; θ, theta; σ, sigma; γ, gamma

**Table 2:** Included evidence in SR with EEG waves and measurement units

#	Author; method	Wave (frequency range)	Sleep parameter (unit); definitions, where provided
1	Armitage (1995); EEG	Delta (0.5– < 4 Hz) Theta (4– < 8 Hz) Alpha (8– < 12 Hz)	• Delta, theta, and alpha power (µV^2^); absolute value of the area of the half-wave squared by frequency category
2	Armitage et al. (2000); EEG	Delta (0.5– < 4 Hz)	• Delta power (µV^2^); derived from power spectral analysis which generated a vector of data describing power in the delta band • Delta amplitude (µV^2^); half-wave zero-cross analyses; cumulative squared voltage of all points in the delta zero-cross bin using period-amplitude analysis
3	Baker et al. (2012); EEG	Delta (0.3– < 4 Hz) Theta (4– < 8 Hz) Alpha (8– < 12 Hz) Sigma (12– < 15 Hz) Beta 1 (15– < 23 Hz)	• Power (µV^2^⋅s); derived from power spectral analysis • Amplitude (µV); zero-cross measures using period-amplitude analysis Power spectra and period amplitude values were averaged across NREM sleep (Stages 2–4) and REM sleep separately for the entire night
4	Campbell et al. (2005); EEG	Delta (0.3–3 Hz) 3–4 Hz Theta (4–6 Hz; 6–8 Hz) Alpha (8–12 Hz) Sigma (12–15 Hz) Beta (15–23 Hz; 23–30 Hz) 30–50 Hz *Frequencies inferred from study Table 4 and text in results section	• Power density (µV^2^⋅sec); power per minute of NREM • Delta amplitude (µV); half-wave detection by zero crossing using period-amplitude analysis
5	Campbell et al. (2012); EEG	Delta (1–4 Hz) Theta (4–8 Hz)	• Theta and delta power (µV^2^); spectral analysis • Theta and delta power decline (µV2); wave power at each semiannual recording plotted against age for each male and each female person. The delta power decline across adolescence was fit with a Gompertz equation: $Power=D-A{\bullet e}^{{-e}^{-C(age-M)}}$ • Delta amplitude (NR): from the upper to lower asymptotes of the delta curve
6	Carrier et al. (2001); EEG	0.25–32z bins were collapsed into 1–Hz frequency ranges (1 Hz: 0.25–1.00 Hz, 2 Hz: 1.25–2.00 Hz, 3 Hz: 2.25–3.00 Hz, etc.)	• Spectral power density (log transformed); power densities were calculated on the 128 Hz signals for consecutive 4-s epochs and 0.25 Hz frequency bandwidths by a Fast Fourier Transform routine. Awake time was excluded based on visual scoring in 60-s epochs. Artifact-laden 4-s epochs were identified by automated procedures. Average power density was calculated as the mean of the artifact-free 4-s sleep epochs for each N-REM period. For comparison of power spectral densities, the 0.25-Hz bins were collapsed into 1-Hz frequency ranges (~ 1 Hz: 0.25–1.00 Hz, 2 Hz:1.25–2.00 Hz, 3 Hz: 2.25–3.00 Hz, etc.)
7	Carrier et al. (2011); EEG	1–4 Hz < 4 Hz *Focuses on slow wave; inferred as Delta	• SW amplitude (µV); difference in voltage between negative and positive peak of unfiltered signal expressed in microvolt • SW slope (µV/s); slope between negative peak and positive peak
8	Dijk et al. (1989); EEG	0.25–15 Hz *Frequencies bands inferred from commonly used categorizations	• Power density, expressed as a percentage (females as % of the average value in males); spectral analysis
9	Dorokhov et al. (2024); EEG	Delta (1–4 Hz) Theta (5–8 Hz) Alpha (9–12 Hz) Sigma (13–16 Hz)	• Spectral power density (ln-transformed); calculated from EEG signals recorded at Fz, F4, Cz, Pz, and O2, referenced to M1/M2. Artifact-free epochs were processed using Fast Fourier Transform. Analysis focused on the first 16 frequency bands, from 1 to 16 Hz (e.g., 0.50–1.49 Hz for 1 Hz, 1.50–2.49 Hz for 2 Hz, etc.)
10	Feinberg et al. (2006); EEG	Delta (0.3–3 Hz) 1–4 Hz	• Delta power density (µV^2^sec/1000); power in 0.3–3 Hz divided by minutes of artifact-free NREM
11	Fukuda et al. (1999); EEG	Delta (0.5–2 Hz, 2–4 Hz)	• Delta spectral power; calculated by Fast Fourier Transform over 1024 data points with a cosine bell window
12	Hejazi et al. (2024); EEG	Delta (0.5–4 Hz): Low Delta (0.5–2 Hz) High Delta (02–4 Hz)	• Delta power spectral density (µV^2^/Hz); analyzed from C3-A2 and C4-A1 signals (filtered 0.5–30 Hz) during NREM using Welch’s periodogram (0.2 Hz and 1 Hz bins, six 5-s epochs, Hamming window) and a Chebyshev Type II filter
13	Kluge et al. (2010); EEG	Delta (0.5–4 Hz) Theta (4.5–8 Hz) Alpha (8.5–12 Hz) Sigma (12.5–16 Hz) Beta (16–20 Hz)	• Delta, theta, alpha, sigma, and beta power (µV^2^); Fast Fourier Transform routine using a rectangular window for consecutive, non-overlapping 2 s miniepochs
14	Latta et al. (2005); EEG	Delta (0.5–4 Hz) Alpha (8.5–12 Hz)	• Delta and alpha activity (µV^2^); absolute spectral power in the 0.5–4 Hz and 8.5–12 Hz frequency bands, respectively • Total power (µV^2^); calculated over the frequency range 0.5 to 12.5 Hz • Relative delta and alpha activity (%); percentage of the total power for each 30-s epoch
15	Luo et al. (2024); EEG	Delta (0.5–4 Hz), Theta (4–8 Hz), Alpha (8–13 Hz), Beta (13–30 Hz), Spindle (11–16 Hz), Sawtooth (3–7 Hz)	• Functional connectivity, quantified using mutual information (MI); MI evaluated both amplitude and phase information between EEG signals, classified as segments using 0.5–30 Hz band-pass filtering
16	Ma et al. (2011); EEG via PSG	Delta (0.5–4.25 Hz) Theta (4.25–8 Hz) Alpha (8–12 Hz) Sigma (12–15 Hz) Beta (15–32.5 Hz) Spindle (11–14 Hz)	• Delta, theta, alpha, sigma, beta, spindle spectral power density, presented as a ratio (females as % difference to males)
17	Markovic et al. (2020); EEG	Delta (1–4.6 Hz) Theta (4.8–7.8 Hz) Alpha (8–10.8 Hz) Sigma (11–16 Hz) Beta 1 (16.2–20 Hz) Beta 2 (20.2–24 Hz) Gamma 1 (24.2–34 Hz) Gamma 2 (34.2–44 Hz) Spindle (10–16 Hz): Slow spindle (10–12 Hz) Fast spindle (12–16 Hz)	• Absolute power (µV^2^); calculated per epoch using 5-s windows, Hanning window, no overlap • Normalized power (µV^2^); power at each derivation normalized by the total power across derivations • Spindle amplitude (µV); not defined • Spindle density (num/s); not defined • Connectivity, measured with coherence; index of brain connectivity during sleep reflecting interactions between spatially segregated populations of neurons. Calculated between all possible channel pairs (i.e., 1653 connections) as (|P_xy_ (f)|^2^)/P_xx_ (f)P_yy_ (f), where P_xy_(f) is the cross-spectral density and P_xx_(f) and P_yy_(f) are the auto-spectral density functions of the two
18	Mongrain et al. (2005); EEG via PSG	Delta (0.75–4 Hz) Theta (4–8 Hz) Low Sigma (12–14 Hz) High Sigma (14–16 Hz)	• Spectral power; Fourier transforms performed on 4-s artifact-free sections using a cosine window tapering
19	Mourtazaev et al. (1995); EEG via PSG	Slow Wave (0.5–2 Hz) *Focuses on slow wave; inferred as Delta	• Slow wave power (µV^2^); manual and computer-estimated based on the band-pass filter (0.5–2.0 Hz) described by Kemp and Lopes da Silva
20	Pun et al. (2023); EEG via PSG	Spindle (11–16 Hz)	• Spindle density (# events per min); calculated by counting spindles in NREM 2 & 3 separately from the central and frontal electrodes, then dividing by the time spent in each stage. Used computerized detection algorithm (11–16 Hz band-pass)
21	Ricci et al. (2021); EEG	Sigma (11–16 Hz) Spindle (10–16 Hz) Fast Spindle (12–16 Hz)	• Sigma power (µV^2^); computed by summing the power density data (including lower and upper limits of the frequency band), adjusting for rejected epochs, averaging for C3 and C4 • Spindle density (spindles/min); total number of spindles in N2 sleep and divided by the time in minutes of N2 sleep • Spindle power (µV^2^); highest power S within the spindle • Fast spindle percent (%); percent of fast spindles out of all identified spindles • Percent change in spindle/sigma activity: calculated as a percent change with the formula [(follow-up value—baseline value)/baseline value]*100. Linear models wee used to calculate age-related percent change as a function of groups: 12–14 years, 15–17 years, and 18–22 years
22	Ringli et al. (2013); EEG	Slow Wave (0.75–4.5 Hz)* *Focuses on slow wave; inferred as Delta	• Power, measured as slow wave activity (µV^2^); calculated as mean power in the range of 1–4.5 Hz during the first 60 min of NREM sleep stages 2 and 3
23	Rosinvil et al. (2021); EEG via PSG	Slow Wave (PtP ≥ 75 μV and NegA ≥ 40 μV, negative to positive deflection ≥ 125 to ≤ 1,500 ≤ 1,000 ms) *Focuses on slow wave; inferred as Delta	• Slow wave density (number of slow waves per minute of NREM sleep, N2–N3 combined) • Slow wave peak-to-peak amplitude (µV); difference in voltage between negative and positive peak of filtered signal, averaged over all-night NREM with N2–N3 combined • Slow wave slope (µV/sec); ratio between the peak-to-peak amplitude and the delay between the two peaks
24	Ujma et al. (2019); EEG via PSG	Sigma (11–15.75 Hz) Alpha (8–10.75 Hz) Beta (16–25 Hz) Gamma (25.25-48 Hz)	• Functional connectivity, measured as weighted phase-lag index (WPLI); calculated between all possible electrode pairs from the resulting time–frequency data and finally averaged across all data segments and electrode pairs belonging to the same cluster, respectively
25	Ujma et al. (2022); EEG	Beta (16–30 Hz) Low Sigma (10–12.5 Hz) High Sigma (12.5–16 Hz) **(**0.01 Hz–4 Hz w 0.01 Hz increments)	• Amplitude; not defined
26	Ventura et al. (2022); EEG	Spindle (~ 11–15 Hz)	• Spindle spectral power, median (µV^2^); calculated from power spectral density estimates of each spindle • Spindle density (spindles/min); number of sleep spindles per minute of NREM
27	Yoon et al. (2021); EEG via PSG	Delta (0.75–4 Hz) Theta (4–8 Hz) Alpha (8–12 Hz) Sigma (12–14 Hz) Beta (14–30 Hz)	• Absolute spectral power (µV^2^, ln transformed); calculated the spectral band power for each 30-s epoch using Fast Fourier Transform with a Hanning window (3-s sliding window with 50% overlap). Spectral power was calculated for the whole night of sleep, REM and NREM sleep, and each stage of NREM including N1, N2 and N3 • Relative spectral power (%); calculated for each frequency band (delta [0.75–4 Hz], theta [4–8 Hz], alpha [8–12 Hz], sigma [12–14 Hz] and beta [14–30 Hz]) by dividing the power of each band by the total EEG power (0.75–30 Hz) at C4 electrode
28	Yuksel et al. (2021); EEG	Delta (0.3–4 Hz) Theta (> 4–8 Hz) Alpha (> 8–12 Hz) Sigma (> 12–15 Hz) Low Beta (> 15–23 Hz) High Beta (> 23–30 Hz)	• Relative power (ratios); calculated for each frequency band as a function of the EEG total power (1–30 Hz), and then averaged across C3 and C4
29	Zhang et al. (2021); EEG	Spindle (12–15 Hz)	• Spindle amplitude (µV); calculated the peak-trough amplitude as the averaged amplitude difference between each sorted amplitude of peak and trough • Spindle density (#spindles/min); calculated as the average number of spindles per minute of NREM artifact-free sleep

### Study sample characteristics

The 28 studies included in the systematic review involved a total of 3,374 participants (47% male participants) [5, 17–43]. The percentage of male participants in each of the included studies ranged from 33% [34] to 67% [38]. The age of participants (in years) ranged from 4-5 months [42] to 101 [36, 37]. The smallest sample size was 16 participants [33] and the largest was 822 participants [31].

Twenty-eight studies reported on sex differences among their participants [5, 17–43]. One study (4%) included participants solely in the infancy and toddlerhood stage of development (4–5 months of age) [42], six studies (21%) included participants soleley in early, middle, and late childhood and adolescence [5, 20, 22–25]; five studies (18%) included participants solely in emerging and early adulthood [18, 19, 26, 30, 32]; one study (4%) examined middle adulthood [35]. No studies investigated sex differences solely in the late adulthood developmental stage. Fifteen studies (54%) included participants spanning age-related developmental stages [17, 21, 27–29, 31, 33, 34, 36–41, 43]. Detailed characteristics of the included studies can be found in Table 1.

### EEG markers and parameters

#### Overall characteristics

Twenty-three studies (82%) reported results on sex differences in the parameters of the delta wave frequency [5, 17–24, 26–37, 39, 43]. Sex differences in the theta wave frequency were reported in 14 (50%) studies [5, 17, 18, 20, 21, 23, 24, 26, 27, 30–32, 36, 43], alpha in 14 (50%) studies [5, 17, 18, 20, 21, 24, 27, 30–32, 35, 36, 41, 43] and sigma in 12 studies (43%) [5, 17, 20, 21, 24, 26, 30–32, 40, 41, 43]. Ten studies (36%) reported on sex differences in beta wave frequency [5, 17, 20, 21, 24, 31, 36, 40, 41, 43] and three studies (11%) in gamma [5, 24, 41]. Six studies (21%) reported on spindles [5, 25, 27, 36, 38, 42] and one study (4%) on sawtooth [36] waveforms.

We observed considerable between-study variations in the recording techniques, in the frequency band definitions and categorization among the included studies, irrespective of whether the data emerged from EEG as part of polysomnography, or standalone EEG. We refer the reader to Table 2 for definitions of the studied parameters.

**Fig. 2:**
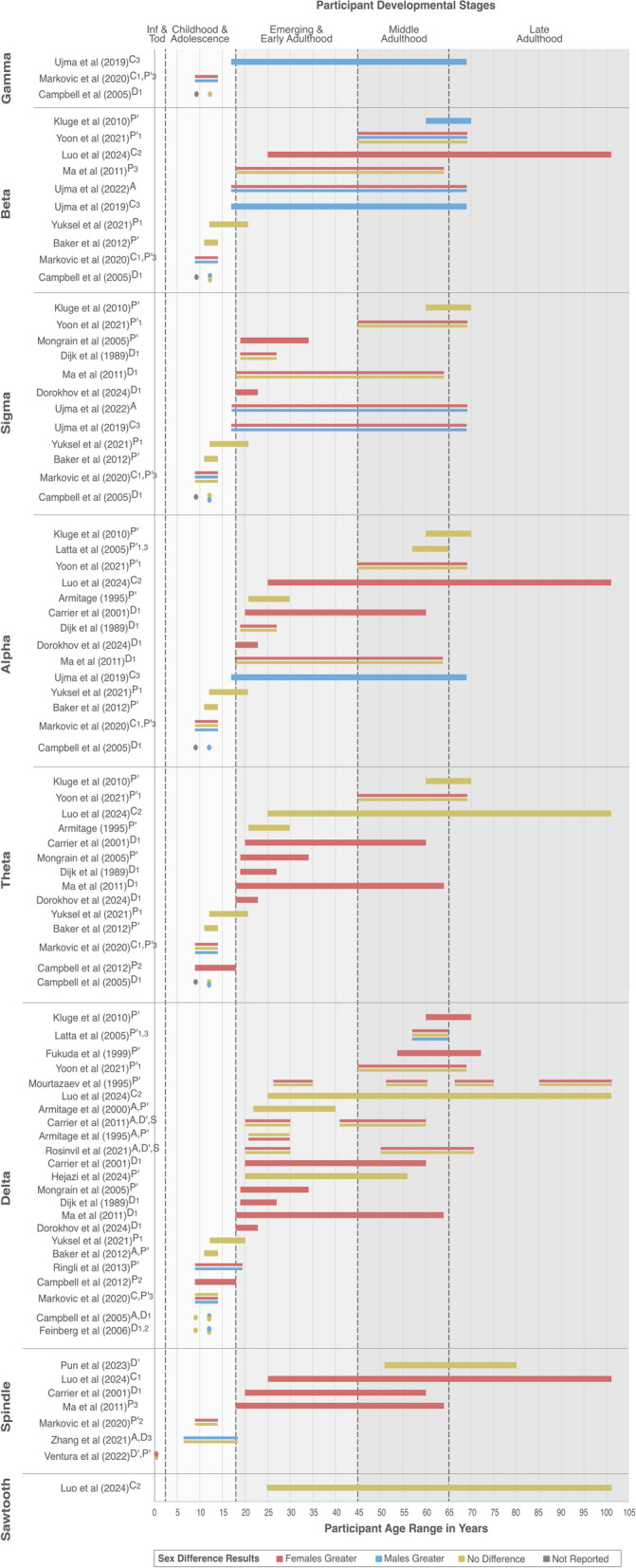
Sex differences in sleep parameters, by brain wave frequencies. Color indicates sex differences in sleep marker values: females greater (pink), males greater (blue), no difference (yellow), grey (not reported). Superscript indicates parameter(s) investigated in the study: A, amplitude; C1, coherence; C2, mutual information; C3, weighted-phase lag index; D’, density; D1, power density; D2, change in power density; D3, density peak; P’, power; P1 relative power; P2, change in power; P3, normalized activity. Length of bars indicates the age range of participants in each study; a circle indicates that a cohort of a single age was included. Please refer to the Results section and Fig. 3 for studies reporting multiple sex difference results

**Fig. 3:**
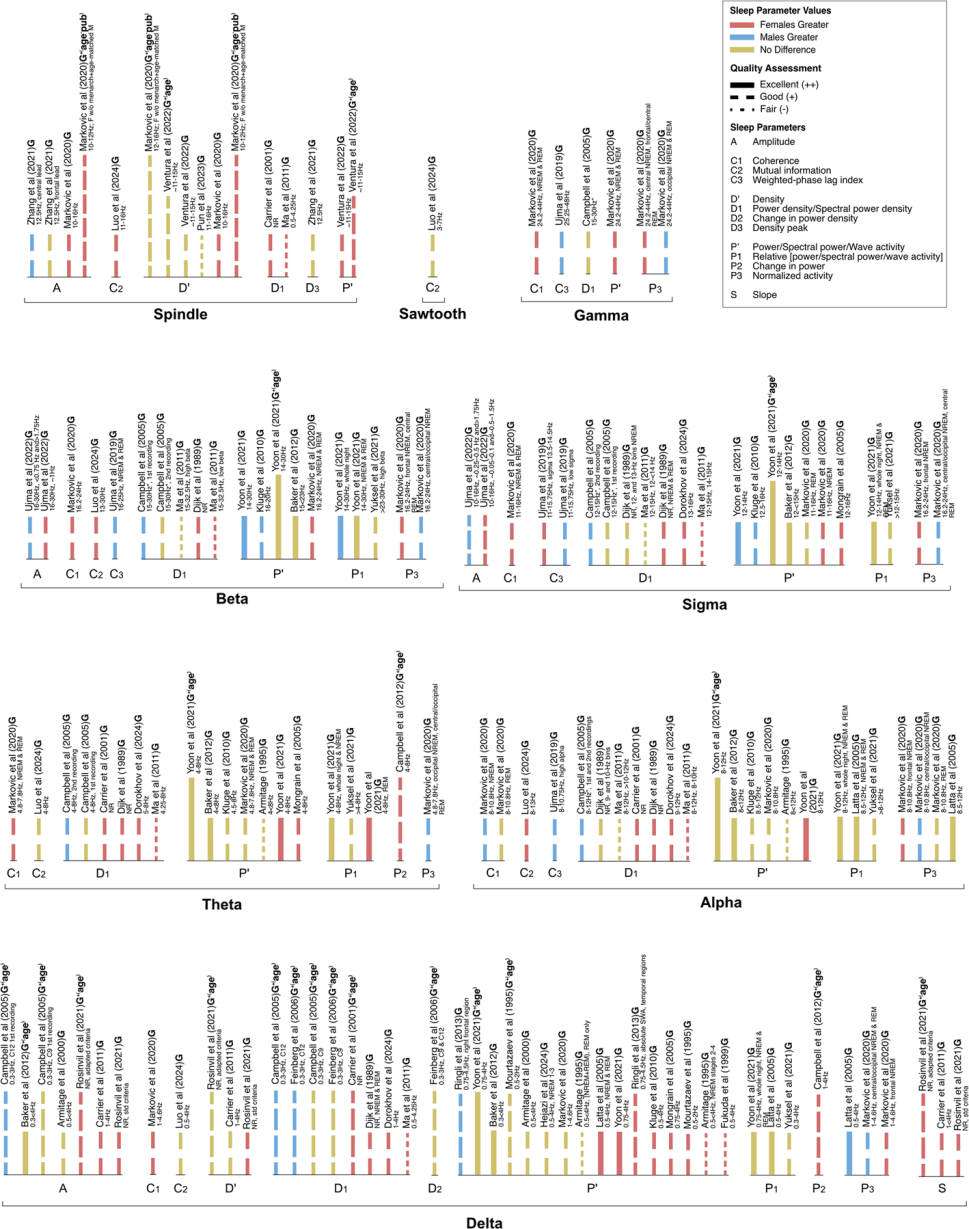
Sex differences in sleep parameters, by brain wave frequencies. Labels indicates the parameter investigated in the studies: A, amplitude; C1, coherence; C2, mutual information; C3, weighted-phase lag index; D’, density; D1, power density; D2, change in power density; D3, density peak; P’, power; P1 relative power; P2, change in power; P3, normalized activity. Color indicates sex differences in sleep marker values: females greater (pink), males greater (blue), no difference (yellow). Length of bars corresponds to number of PROGRESS-Plus parameters considered in statistical analysis of the study, shown as a superscript: G, gender/sex; + , plus (additional parameters, shown in parentheses). Line style corresponds to Quality Assessment of the study: Excellent (+ + , thick solid lines), Good (+ , medium dashed lines), Fair (-, thin dotted lines)

We observed variations in the reporting of the age of participants. Twenty-two studies (79%) reported age of their samples as means and standard deviations (SD) [5, 17–20, 22, 24, 26, 28, 29, 31–36, 38–43]. Fifteen studies (54%) reported both mean with SD and age ranges [5, 17, 26, 29, 31, 33, 34, 36, 38–43], and three studies (11%) [25, 27, 37] reported only age range, which in some cases spanned several decades (Table 1, Fig. 2). This variation required us to use age-related developmental stages to contextualize the evidence and preserve all the data in data synthesis (Fig. 2). This allowed us to make comparisons of results across similar age groups. The decision to combine specific age-related developmental stages was made since few studies had a sample that could be categorized under a singular age-related development stage. Studies reported on several sleep wave parameters (Fig. 3).

#### Sex differences in delta waves

Twenty-three included studies (82%) provided relevant information on sex differences in delta frequency waves [5, 17–24, 26–37, 39, 43], across various age-related stages of development: five (18%) of these studies included participants  in childhood and adolescence [9, 20, 22–24], five (18%) in emerging and early adulthood [18, 19, 26, 30, 32], and one (4%) in middle and late adulthood [35]*.* Twelve studies (43%) included participants spanning across several age-related developmental stages [17, 21, 27–29, 31, 33, 34, 36, 37, 39, 43].

##### Early, middle, and late childhood and adolescence:

Campbell et al. (2005) reported no sex differences in delta amplitude and power density in the 9-year-old cohort; however, sex differences were observed in the 12-year-old cohort, where male participants expressed higher values in both amplitude and power density compared to female participants [24]. In female participants only, of both age cohorts, researchers observed weak negative correlations between power density and Tanner stage, which was observed in the first recording but not second, which took place 6 months after the first[24]. Campbell et al. [23] reported on change in power as participants aged (i.e., from age 9–16 (cohort 1) and age 12–18 (cohort 2)) [23]. Researchers found that the age of steepest delta power decline differs between the sexes, with decline occurring at an earlier age in females [23]. Feinberg et al. [22] reported no sex differences in power density for the 9-year-old cohort; in the 12-year-old cohort, male participants had greater power density as compared to female [22]. Delta power density did not change between ages 9 and 11 for both sexes; however, it declined between ages 12 and 14 in both sexes, with no sex differences in the rate of decline [22]. Baker et al. [20] found no sex differences in delta power and amplitude in their participants aged 11–14 years [20]. Markovic et al. [5] in their participants aged 9 to 14 years, reported no sex differences in absolute power across brain regions in NREM and REM sleep; however, when the data were normalised, sex differences were observed, with male participants showing greater power in the central/occipital region during both REM and NREM sleep, and female in the frontal region during NREM [5]. This group also reported differences in the connectivity, expressed as coherence, with female participants having greater values in NREM and REM sleep [5]. Ringli et al. [39] observed increased delta power from the ages of 10–17 in male participants in the right frontal region; female participants had higher power values in the left and right temporal regions [39].

##### Emerging and early adulthood:

Dijk et al. [30] reported that female participants aged 19–27 years exhibited higher delta power density during REM and NREM sleep as compared to their male counterparts [30]. In the research by Mongrain et al. [26] with participants aged  19–34 years, researchers found female participants to have higher spectral power as compared to male participants [26]. Armitage [18] found higher overall delta power during NREM stages 2–4 in their female participants aged ~ 20–30 years; during REM and all-night sleep (REM + NREM), no difference was observed [18]. In a study published five years later with participants aged 22–40, Armitage found no sex differences in the amplitude and power of delta sleep [19]. In a study of delta sleep during napping state in participants aged 18–23, Dorokhov et al. found that female participants had greater spectral power density as compared to male [32].

##### Middle and late adulthood:

Latta et al. (2005) reported greater absolute delta power in female participants compared to male during both NREM and REM sleep, but no sex difference in relative delta power. When the data were normalised, sex difference for relative delta power was observed, with male participants expressing greater power.

##### Studies spanning several developmental stages:

Mourtazaev et al. [37] reported greater delta power in female participants in a sample with ages ranging from 26 to 101; the age and sex interaction was not statistically significant [37]. Carrier et al. [27] reported greater power spectral density among female participants aged 20–60, as compared to male [27]; the results were similar in the study by Ma et al. [31], with a sample aged 18–64 [31]. Carrier et al. [28] reported greater delta wave amplitude in female participants. In their analysis of sex differences in density, the researchers found that main sex effect was not statistically significant, but observed statistically significant results for age group, sex, and derivation interaction [28]. Fukuda et al. [33] reported greater spectral power in female as compared to male, in a sample of participants aged 54–72 [33]. Hejazi et al. [34] found no sex differences in NREM (N1-N3) delta power in their participants aged 20–56 years old [34]. Kluge et al. [17] found sex difference in delta power in their sample aged 60–70 years old, with females exhibiting higher values [17]. Yoon et al. [43] reported greater absolute delta power in female participants compared to male, but no sex difference for relative delta power in their sample aged 45–69 [43].

Luo et al. [36] reported no sex difference in functional connectivity, quantified using mutual information, in delta wave frequency in both NREM (N1-N3) and REM in their participants aged 25–101 [36]. Rosinvil et al. [29] reported that female showed greater slow wave density, amplitude and slope as compared to male participants in a sample aged 20–71; however, after application of age-and sex-adapted criteria, sex difference in slow wave density became not statistically significant, while the sex differences in the amplitude and slope remained significant [29].

#### Sex differences in theta waves

Fourteen studies provided results on sex differences in theta waves  across developmental stages [5, 17, 18, 20, 21, 23, 24, 26, 27, 30–32, 36, 43]. Four of these studies included participants in childhood and adolescence [5, 20, 23, 24] and four in emerging and early adulthood [18, 26, 30, 32]. Six studies included participants spanning developmental stages [17, 21, 27, 31, 36, 43].

##### Early, middle, and late childhood and adolescence:

In their cohort aged 12 years, Campbell et al. [24] reported no sex differences in theta power density in NREM sleep in the first recording session, however male participants showed greater values in the second recording session [24]. In the study by Campbell et al. [23], researchers investigated change in power, reporting that the steepest decline in theta power occurred at an earlier age in female participants as compared to male [23]. Markovic et al. [5], in their sample of 9–14-year-olds, reported on three parameters: coherence, absolute power, and normalised power. Compared to male, female participants had greater coherence in NREM and REM sleep, with no sex difference observed in absolute power. When power data were normalized, sex differences were observed in certain topographic regions [5]. Baker et al. [20] reported no sex differences in their 11–14-year-old participants in theta power during NREM and REM sleep [20].

##### Emerging and early adulthood:

Dijk et al. [30] reported that female participants in their sample aged 19–27 years exhibited greater theta power density during REM and NREM sleep as compared to male [30]. Dorokhov et al. [32] reported sex difference in theta spectral power densities, with female participants showing higher power densities across all sleep stages during a nap [32]. In a sample of participants aged 19–34 years, Mongrain et al. [26] found higher power in NREM in female as compared to male participants [26]. Armitage [18] reported no sex difference in all-night sleep theta power in their participants aged ~ 20 to 30 years [18].

##### Studies spanning several developmental stages:

Ma et al. [31] reported female participants had higher power spectral density as compared to male, in a sample aged 18–64 years [31]. Carrier et al. [27] reported higher power spectral density for female participants as compared to male in a sample aged 20–60 [27]. Kluge et al. [17] reported no sex difference in theta power in their sample aged 60–70[17]. Yoon et al. [43] found female participants had greater absolute spectral power using ANOVA; however, sex was not significant in multivariate modeling accounting for sex and age [43]. The researchers reported greater relative spectral power in female participants during REM sleep, and no sex differences in NREM and whole-night sleep [43]. Yuksel et al. [21] reported no sex difference in relative power in NREM in both recording nights [21].

Luo et al. [36] in their sample of participants aged 25–101 years observed no sex difference in theta wave functional connectivity, quantified using mutual information [36].

#### Sex differences in alpha waves

Fourteen studies investigated sex differences in parameters of alpha wave frequency in participants across age-related developmental stages: three of these studies included participants in childhood and adolescence [5, 20, 24], three in emerging and early adulthood [18, 30, 32], and one in middle adulthood [35] (Fig. 2). The remaining seven studies [17, 21, 27, 31, 36, 40, 43] included participants spanning across age-related developmental stages.

##### Early, middle, and late childhood and adolescence:

Campbell et al. [24] reported higher power density for male participants than female in their cohort aged 12 in both recording sessions, which were conducted 6 months apart [24]. Baker et al. [20] found no significant sex differences in alpha power in NREM and REM in their sample of adolescents aged 11–14 years [20]. Markovic et al. [5] found no significant differences in absolute alpha power in NREM and REM in their sample of 9–14-year-olds, although Markovic et al. [5] noted sex differences in normalised power in NREM sleep, with female participants showing greater power in the frontal region and male participants in the central/occipital regions [5]. These researchers also reported on coherence, with male participants showing greater values in NREM, and no sex difference in REM sleep [5].

##### Emerging and early adulthood:

Dijk et al. [30] found that alpha power densities during NREM and REM sleep were higher in female participants, except for the 9- and 10-Hz during REM sleep [30], in a sample aged 19–27. Armitage [18] reported no significant difference in alpha power between the sexes for ~ 20 to 30-year-old participants [18]. Dorokhov et al. [32] observed that female participants demonstrated higher spectral power densities, compared to male in their sample aged 18–23 [32].

##### Middle and late adulthood:

Latta et al. [35] found no sex differences in absolute, relative, and normalised alpha wave activity in their sample aged ~ 57–65 [35].

##### Studies spanning several developmental stages:

Yoon et al. (2021), in their participants aged 45–69 years, reported higher absolute alpha power in female as compared to male using ANOVA; sex was not significant in the multivariate regression accounting for sex and age. No sex difference was observed in relative alpha power during whole night, NREM, and REM sleep [43]. Yuksel et al. [21] found no sex difference in relative power [21]. Kluge et al. [17] found no sex difference in alpha power in participants aged 60–70 years [17]. Ma et al. [31], in their sample aged 18–64, observed a higher power spectral density in their female participants as compared to male, in the frequency band 8–10 Hz and no sex differences for >10–12 Hz [31].

Ujma et al. [41], in their sample aged 17–69 years, reported that males expressed greater alpha wave connectivity, quantified as weighted phase-lag index, in NREM than female participants [40]. Luo et al. [36] observed greater functional connectivity, quantified using mutual information, in the alpha frequency band in female compared to male participants, across all stages of sleep, in participants aged 25–101 years [36].

#### Sex differences in sigma waves

Twelve studies reported results on sigma wave frequency [5, 17, 20, 21, 24, 26, 30–32, 40, 41, 43]. Three studies included participants in childhood and adolescence [5, 20, 24] and three in emerging and early adulthood [26, 30, 32]. The remaining six studies included participants across age-related developmental stages [17, 21, 31, 40, 41, 43].

##### Early, middle, and late childhood and adolescence:

In their cohort of 12-year-olds, Campbell et al. [24] reported higher power density values in male participants relative to female in the second recording, which occurred six months after the first, during which no sex differences were observed [24]. Baker et al. [20] found no sex differences in sigma power in their sample of 11–14 year-olds [20]. Markovic et al. [5] observed greater absolute sigma power in females during NREM sleep in their cohort of participants aged 9–14 years; and no sex difference in REM sleep [5]. When the data were normalised, researchers observed sex differences during NREM and REM sleep across brain regions [5]. The researchers also reported on coherence, with female participants expressing greater values in both NREM and REM [5].

##### Emerging and early adulthood:

Dijk et al. [30] found that sigma power densities during NREM and REM sleep were higher in females, except for the 12- and 13-Hz during NREM sleep [30], in their sample aged 19–27. In the study by Mongrain et al. [26] including participants aged 19–34 years, female participants had greater sigma power in NREM [26]. Dorokhov et al. [32] reported that female participants exhibited higher sigma spectral power density as compared to males [32].

##### Studies spanning several developmental stages:

Ujma et al. [41] in their sample of 17–69 year-olds reported greater connectivity in female participants in the frequency range 13.5–14.5 Hz, and greater values in male participants for the low sigma range [41]. In the study of Ujma et al. [40], researchers reported sex differences in sigma amplitude, in their sample aged 17–69 years [40]. Ma et al. [31] studied power spectral density, reporting no sex difference in the 12 to < 14 Hz range, and greater values among females in the 14–15 Hz range [31]. Kluge et al. [17] reported no sex difference in sigma power in their sample aged 60–70 years [17].

Yoon et al. [43], in their participants aged 45–69 years, reported higher absolute sigma power in female as compared to male participants using ANOVA; sex was not significant in the multivariate regression accounting for sex and age [43]. No sex difference was observed in relative sigma power during whole night, NREM, and REM sleep [43]. Yuksel et al. [21] reported no sex difference in relative sigma power in NREM sleep [21].

#### Sex differences in beta waves

Ten studies explored sex differences in EEG parameters in the beta frequency range in samples that include participants from various age-related developmental stages: three of these studies included participants in childhood and adolescence [5, 20, 24], and the remaining seven studies included participants across developmental stages [17, 21, 31, 36, 40, 41, 43].

##### Early, middle, and late childhood and adolescence:

Campbell et al. [24] reported higher density values in the first night recording among males in the 12-year-old cohort; however, no sex differences were observed in the second recording 6 months later [24]. Markovic et al. [5], in their sample of 9–14-year-olds, found that female participants exhibited greater absolute power; when the data were normalised, sex differences were observed in certain brain regions [5]. The researchers also reported on coherence, with female participants showing greater values in NREM and REM [5]. Baker et al. [20] found no significant difference in beta power in their cohort of 11–14-year-old participants [20].

##### Studies spanning several developmental stages:

Ujma et al. [41] in their sample aged 17–69 observed higher connectivity, quantified as weighted phase-lag index, in male in NREM and REM, as compared to female participants [41]. Luo et al. [36] reported higher functional connectivity, quantified as mutual information, in female compared to male participants in a sample ranging from ages 25 to 101 [36]. In the study of Ujma et al. [40], researchers reported that sex differences exist in beta amplitude, in their sample aged 17–69 years [40]. Ma et al. [31], in the sample of 18–64 year-olds, reported that female participants had a greater power spectral density in the 15–18 Hz range, but no sex difference in beta frequency ranges > 18 Hz [31].

Kluge et al. [17], in their sample aged 60–70, found that male participants had greater beta power [17]. Yoon et al. [43] reported that male participants had lower absolute spectral power in ANOVA, but that sex was not significant in multivariate analysis accounting for age [43]. Males had greater relative power during whole night sleep, but no sex differences were observed in NREM or REM sleep [43]. Yuksel et al. [21] reported no sex differences in relative power [21].

#### Sex differences in gamma waves

Sex difference in gamma waves in sleep was explored in three studies [5, 24, 41]: two studies included participants in childhood and adolescence [5, 24], and one study included participants spanning across developmental stages [41].

##### Early, middle, and late childhood and adolescence:

Markovic et al. [5] reported that female participants expressed greater absolute gamma power in comparison to male in NREM and REM sleep, in their sample aged 9–14 [5]. When the data were normalised, sex differences were observed in specific brain regions [5]. The researchers also reported on sex differences in coherence; results indicated that female participants showed greater values in NREM and REM as compared to male [5].

##### Studies spanning several developmental stages:

Ujma et al. [41] found that male participants exhibited greater connectivity for gamma waves in NREM and REM in their sample of participants aged 17–69 [41].

#### Sex differences in transient waveform events

In addition to reporting on sex difference in EEG waves in specific frequency bands, six groups of researchers reported on sex difference in transient waveforms events known as sleep spindles [5, 25, 27, 36, 38, 42]. One group also reported on sawtooth waves [36], a variant of theta activity, with each wave also containing a notch, making it sawtooth-shaped.

Sex difference in spindles were reported in participants in infancy and toddlerhood in one study [42], in childhood and adolescence in two studies [5, 25], and in participants spanning across developmental stages in three studies [27, 36, 38]. Sex difference in sawtooth waveform was reported in participants spanning age-related developmental stages in one study [36].

##### Infancy and toddlerhood:

Ventura et al. [42] reported that female participants in their sample aged 4–5 months expressed greater spindle spectral power as compared to male [42]. No sex differences were observed in spindle density [42].

##### Early, middle, and late childhood and adolescence:

Markovic et al. [5] reported that female participants in their cohort aged 9–14 expressed greater spindle amplitude in comparison to male [5]. The researchers also found greater spindle density in female participants; however, when the analysis was performed in female participants with no menarche and age-matched male participants, females still expressed greater density in slow spindles; but no sex difference was observed in fast spindles [5]. Zhang et al. [25] reported on sex difference in spindle amplitude in their cohorts aged 9 and 12; results were reported by brain region [25]. For both cohorts, male participants had greater amplitude in the central lead as compared to female; no sex difference was observed in the frontal lead [25]. These researchers also reported on the density peak, with results showing no sex difference for central or frontal leads [25].

##### Studies spanning several developmental stages:

Carrier et al. [27] reported that female participants in their sample aged 20–60 had greater power spectral density and compared to male [27]. Luo et al. [36] reported on sex differences in spindle and sawtooth connectivity, quantified by mutual information, in their sample aged 25–101. Female participants expressed greater spindle connectivity as compared to male, and no sex difference in sawtooth waves [36]. Pun et al. [38] reported no sex difference in spindle density in their participants aged 51–80 [38].

### PROGRESS-Plus considerations

**Fig. 4:**
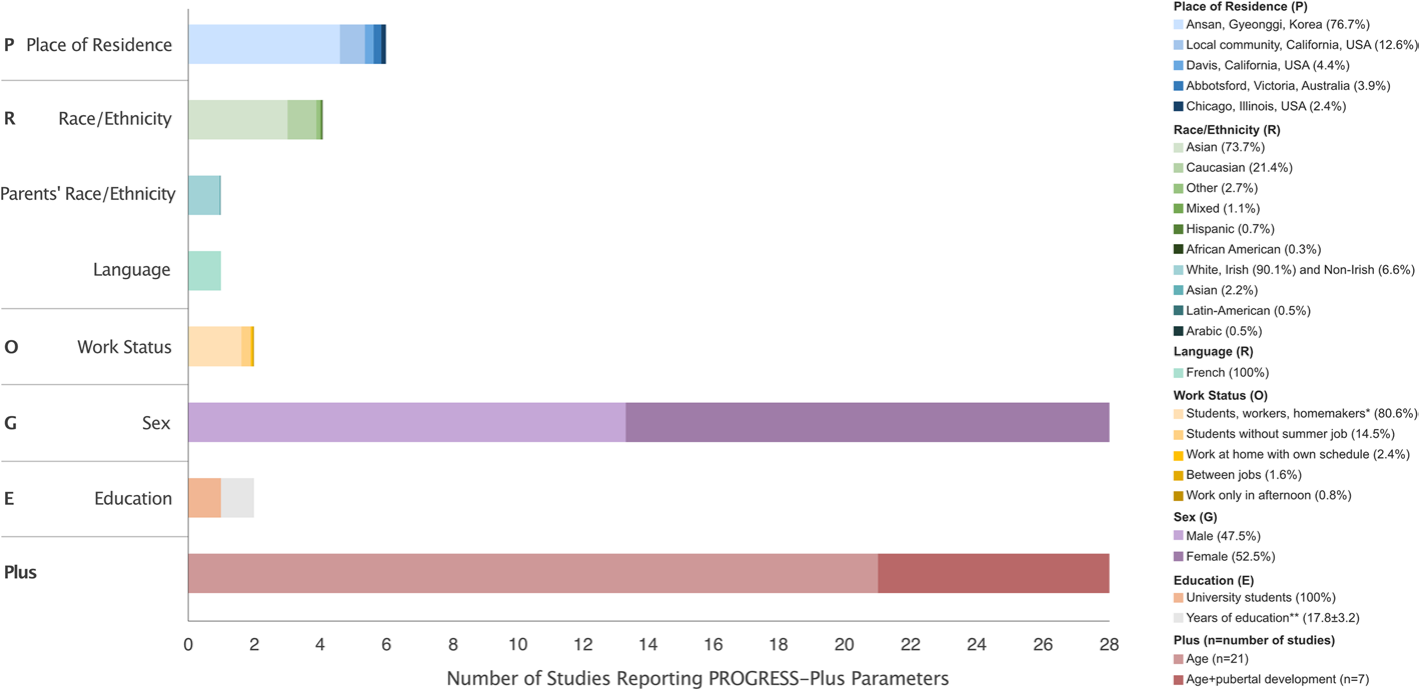
Number of studies reporting at least one PROGRESS-Plus parameter: P, place of residence (blue, n = 6); R, race/ethnicity/culture/language (green, n = 6); O, occupation (yellow, n = 2); G, gender/sex (purple, n = 28); E, education (orange, n = 2); plus, additional parameters (red, n = 28). Stacked bars represent proportion of participants (%) in each category: place of residence, race/ethnicity, language, work status*, sex, and education**. Stacked bars for plus parameters represent number of studies reporting age only (n = 21) or both age and pubertal development (n = 7). *Carrier et al. [27] reported that more than 95% of participants in their sample were students, workers, or homemakers, but did not provide specific breakdown of work status. **Pun et al. [38] reported education in years (mean and standard deviation)

**Fig. 5:**
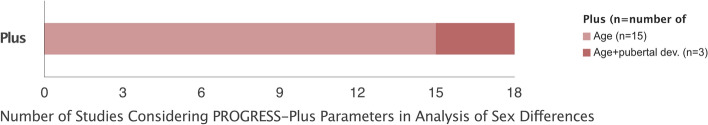
Number of studies considering additional PROGRESS-Plus parameters in analysis of sex differences: age only (n = 15); age and pubertal development (n = 3)

A detailed description of the characteristics of included studies using the PROGRESS-Plus framework can be found in Supplementary Material S2, Figs. 4 and 5.

#### Place of residence of participants

Seven studies reported on participants’ place of residence [20–23, 35, 37, 43]. These seven studies included participants from Abbotsford (Australia) [20], Davis [21–23] and Chicago (USA) [35], Ansan (Korea) [43], and Leiden (Netherlands) [37].

Although only indirectly linked to participants’ place of residence, the country in which research was conducted is important to consider, as participant recruitment often occurs locally. Out of the 28 studies, most studies were conducted in high-income countries. Eleven studies were conducted in the USA [18, 19, 21–25, 27, 31, 34, 35], four in Canada [26, 28, 29, 38], and two in Switzerland [5, 39], the Netherlands [30, 37] and Germany and Hungary [40, 41], each. One study was conducted in each of the following countries: Ireland [42], Australia [20], Germany [17], Korea [43], Japan [33], and Russia [32]. Luo et al. [36] used Sleep-EDF Database from the Research Resource for Complex Physiologic Signals established under the auspices of the National Institutes of Health, but did not specify the country of data origin [36].

#### Race/Ethnicity/Culture/Language

Five studies reported on race/ethnicity of research participants or their parents [21–23, 42, 43] and one study reported on language [26]. Yoon et al. [43] noted in the discussion that race has influence on morphological features due to genetic factors, which may be the reason for the absence of sigma peaks in Koreans [43].

#### Occupation

Two studies [26, 27] reported on occupation of their participants. The majority of participants (> 95%) in the study by Carrier et al. [27] were students, workers, and homemakers [27]. Out of the 24 participants in the study by Mongrain et al. [26], 18 were students (without a summer job), three worked at home with their own schedule, two were in between jobs, and one participant worked afternoon shifts [26].

#### Education

Two studies reported on the education level of their participants [32, 38]. In the study by Dorokhov et al. [32], participants were university students [32]. In the study by Pun et al. [38], the researchers reported years of education of their participants: 18.1 ± 4.3 years for males and 17.7 ± 2.7 years for females [38].

#### Remaining parameters (gender, religion, social capital, and socioeconomic status)

Gender as a sociocultural construct [1], religion, social capital, and socioeconomic status of research participants were not reported in the studies included in this review.

#### Plus

We observed a variety of statistical approaches and Plus variables considered in the study of sex differences in sleep wave parameters (Supplementary Material S3).

##### Age

All 28 studies reported the participants’ age as mean and standard deviation, range, or both [5, 17–43]. One study reported on gestational age and post-natal age of their research participants [42]. Of these, 18 studies considered age in the analyses of sex difference [5, 17, 19, 20, 22–25, 27–29, 34, 36, 37, 39–42].

##### Pubertal stage

Seven studies reported on pubertal stages of their research participants [5, 20–24, 39], of which six used Tanner stage to measure pubertal development [5, 20, 22–24, 39] and one used the Pubertal Developmental Scale [21]. Of these, three studies considered Tanner stage in the analyses of sex differences [5, 22, 23].

### Risk of bias in studies and certainty of the evidence

We used the NIH tool to rate studies an “excellent” (i.e., low risk of bias), “good” (i.e., moderate risk of bias), “fair” (i.e., high risk of bias) rating (Supplementary Material S4). The criteria for the six potential sources of bias were rated as “Yes”, “No”, “Cannot determine”, “Not applicable” or “Not reported”. If an item was rated as “No”, it was regarded as a potential risk of bias. The challenge faced by study authors in assessing risk of bias using the NIH tool was in designating “Cannot determine” or “Not reported” versus a definite rating. For instance, if little information was provided for Item 5 (“Was a sample size justification, power description, or variance and effect estimates provided?”), the authors were left with no clear indicators to distinguish “Cannot determine” (described all or some components but not in detail) versus “Not reported” (did not describe). Thus, we rated studies that provided at least some information as “Yes” for a conservative evaluation. Likewise, Item 11 (“Were the outcome measures [dependent variables] clearly defined, valid, reliable, and implemented consistently across all study participants?”) was rated “Yes” because the minimal requirement was agreed to be a standardized measure of outcome. Thus, we rated three studies as “excellent” [20, 35, 43], 21 studies as “good” [5, 17, 19, 21–30, 32, 34, 36, 37, 39–42], and four studies as “fair” [18, 31, 33, 38] (Supplementary Material S4).

Figure 3 positions results based on frequency bands, sleep wave parameter, quality assessment, and PROGRESS-Plus variables considered in the analysis of sex difference. Using the criteria for certainty assessment outlined in the methods section, sex differences in alpha and delta relative power [35, 43], reported in two studies of excellent quality, were assessed as high in certainty. Results suggest moderate certainty that no sex differences exist in alpha power [5, 17, 20, 43], in theta [21, 43], sigma [21, 43], and beta relative power [21, 43], and in delta density [28, 29]. There is also moderate certainty that female participants had greater delta slope [28, 29], and male participants had greater normalized delta power [5, 35]. Low certainty evidence points to sex difference in spindle power density, with greater values in female [27, 31] compared to male participants. Evidence for all other sleep parameters was assessed as very low in certainty.

## Discussion

### Summary

In our systematic review, we included 28 studies that assessed sex differences in sleep wave parameters across ages and age-related developmental stages. We did not observe consistently reported differences in sleep wave parameters between male and female persons during ages that are known to be linked to sex steroid hormone differentiation (i.e., adolescence, menopause) and, therefore, we cannot conclude that the reported differences are driven by biology (i.e., sex). The results, nonetheless, provide an opportunity for a unique discussion with both research and clinical implications.

The strongest emerging pattern was that the majority of studies investigating parameters of delta, theta, and spindle waveform reported greater values among female participants as compared to male, or no sex difference (Fig. 2). The majority of these studies included samples that spanned adulthood developmental stages. When it comes to sigma, beta, and gamma waves, there were less consistent results on sex differences. Despite observed trends, the certainty of evidence for most studied parameters was very low. The varied results regarding sex differences in sleep parameters may suggest that physiological and pathophysiological processes in sleep reflect the great variability in a person’s environmental and social influences (i.e., PROGRESS-Plus), as opposed to biological sex, which were rarely considered in data analysis of sex difference. In light of this, we conclude that future studies on sex differences in sleep parameters that integrate social variables hold great potential to advance our understanding of the brain activity in sleep on a lifespan continuum.

### Sleep wave parameters

Various sleep waves have been reported to oscillate differently during stages of sleep and levels of wakefulness [44]. The infra-slow oscillations (ISO, < 0.5 Hz), most prominent during non-rapid eye movement (NREM) sleep, have been suggested to express the overall neuronal connectivity of the brain. During wakefulness, these ISO are less prominent than in sleep, and they have been linked to baseline cortical excitability and large-scale network coordination, influencing vigilance and the transition between sleep and wakefulness [44, 45]. In our review, we did not observe studies that reported on sex differences in ISO, which position these waves as a priority to fill this knowledge gap. This is especially relevant because no studies included in this review connected results on sex differences in sleep wave parameters to EEG activity during waking state.

Delta waves (i.e., usually ~ 0.5 to 4 Hz frequency band, but defined differently by each study author, see Table 2) dominate during deep sleep (N3 sleep, also known as slow wave sleep (SWS)) and are essential for restorative and neuroplasticity processes, including tissue repair and memory consolidation [46]. In the waking state, occurrence of delta waves suggests compromised brain processes, including exhaustion and extreme fatigue [44]. Several studies included in this review reported on sex differences in a number of delta wave parameters, illustrated in Figs. 2 and 3. The studies to date did not, however, link the results concerning sex difference to the restorative and neuroplasticity processes in their study samples.

Theta waves (i.e., usually ~ 4 to 7 Hz frequency band, but defined differently by each study author, see Table 2) emerge during the transition from wakefulness to sleep, and are present during REM sleep [44, 47]. In the waking state, theta activity is associated with drowsiness or a meditative state [46]; however, when excessive, can be indicative of attentional deficits, frustration, annoyance, and embarrassment [48, 49]. Frontal theta waves in the waking state are linked to tasks involving working memory, attention, and emotional processing [50]. In sleep, one study included in this review reported on sex differences in central and occipital region during the childhood and adolescence, with male participants showing greater values [5]. The significance of this finding remains to be established.

Alpha waves (i.e., usually ~ 8 to 12 Hz, but defined differently by each study author, see Table 2), prominent during relaxed wakefulness, particularly when the eyes are closed and the person is in a calm, resting state, have been associated with introspection, relaxation, and inhibition of active processing during tasks requiring focused attention [44]. These waves start to diminish with sleep onset, giving way to slower EEG rhythms [47]. The sex-specific results of studies included in this review were mixed, with some results pointing towards greater values for female participants in alpha power density [27, 30–32], and others reporting no differences, primarily in power [5, 17, 18, 20, 43] and relative power [21, 35, 43]. Several studies included participants spanning a wide range of ages, which could affect precision in analysis and reporting, as dominant influences from specific ages in relatively small sample sizes of studies included in the review may skew results for the entire range in either direction [27, 31, 36, 41, 43]. Future studies on sex differences in alpha activity in sleep are greatly needed.

Sigma waves (i.e., usually ~ 12 to 16 Hz frequency band, but defined differently by each study author, see Table 2) play a critical role in memory consolidation and synaptic plasticity [44, 51, 52]. While sigma activity is not typically observed during wakefulness, it represents a key marker of sleep depth and stability [44, 51]. Sex differences in sigma wave parameters reported in studies included in this review were mixed, with no clear direction to discuss the results and their relevance to the waking state (Figs. 2 and 3).

Beta waves (i.e., usually ~ 13 to 30 Hz frequency band, but defined differently by each study author, see Table 2), most prominent during wakefulness, particularly during active cognitive processing, problem-solving, and decision-making, are generally distributed over the frontal and central regions of the brain [44]. During sleep, beta activity decreases significantly but can reappear transiently during periods of arousal or light sleep [44] and in REM [53]. Further exploration on sex differences in beta activity in sleep is needed, since the results of several studies reported contrasting results regarding sex difference in the same parameters, such as power density [24, 30, 43] and power [5, 17, 20, 43] (Fig. 3).

High-frequency oscillations (HFOs, > 30 Hz) include gamma waves (30–80 Hz), ripples (80–200 Hz), and fast ripples (200–500 Hz) [44]. Gamma waves are reported to be associated with higher cognitive functions, including attention, memory encoding, and sensory perception, and are present during both wakefulness and REM sleep [44, 47]. Ripples and fast ripples are typically associated with epileptic activity but, during wakefulness, they may also reflect high-level cognitive processes and rapid information processing [44]. Three studies included in this review reported on sex differences in gamma waves, but on different parameters [5, 24, 41]. The evidence is insufficient to discuss with any degree of certainty and emphasizes the need for further investigations.

Sleep spindles are believed to protect sleep by inhibiting external sensory input, and their frequency and distribution are linked to cognitive performance and development [47, 51]. Sleep spindles are generated by an interplay between neurons in the thalamic reticular nucleus and the relay nuclei and conveyed to the cortex by the pattern of burst firing [54]. Reported sex differences in amplitude, density, power, and connectivity of sleep spindles [5, 25, 27, 31, 36, 38, 42] could be linked to differences in the structure of the thalamus or deafferentation of the cortex, synaptic connectivity among the cell types and corticothalamic projections, differences in neural processing, input from arousal systems and hormones, among many other influences, that are believed to play an important role in maintaining NREM sleep continuity. However, results on sex difference in fast and slow spindles [5] may suggest differences in activation patterns between the two, as fast spindles have been shown to be associated with increased activity in sensorimotor areas, the hippocampus, and the mesial frontal cortex, while slow spindles with activation in the superior frontal gyrus [55]. Study of sex difference in sleep spindles are of interest to different disciplines in health research, due to their implications in learning and memory [56], and in sensory gating [57, 58] during sleep.

Sawtooth waves are produced in the transition to and throughout REM sleep. A recent study has reported a large set of regions in the parietal, frontal, and insular cortices that have shown increases in 2–4 Hz power during sawtooth activity, associated with a strong and widespread increase in high frequencies, suggesting that the waves may be involved in cognitive processes during REM sleep [59]. The cortical generators and functional significance of the waves and results on no sex difference in one study included in this review [36] need to be replicated before definite conclusions can be drawn. Because of the association between REM sleep and dreaming state, it is also possible that that the link exists between the intensity of the visual nature of dreaming and the occurrence of sawtooth waves. The validity of these hypothesis requires further study.

#### PROGRESS-Plus considerations

The EEG data of male and female participants recruited to studies concerning sex difference synthesized in this review likely reflect participants’ diverse and complex brain activity at the time of investigation. This activity reflects, at least partially, aspects of social identity and a variety of socio-cognitive processes on a time continuum uniting sleep and waking states [60]. We aimed to capture participant characteristics and social identities through analysis of PROGRESS-Plus parameters considered in research. We observed that only age and pubertal stage were considered in data analyses of sex difference. The influence of age on sex differences in sleep waves reported in number studies included in this review (Table 1), should be viewed from the lens of social parameters, which received no consideration in data analysis. It is becoming increasingly clear that these parameters have influence on the brain’s structure and function in significant ways that must be considered in sleep research [61, 62].

### Study strengths & limitations

This is the first comprehensive systematic review aggregating data from published studies to examine sex differences in different sleep wave parameters. Our subgroup analyses and visual data presentation allows for nuanced comparisons across studies. We have presented data on the PROGRESS-Plus-related characteristics of study samples, allowing discussion of generalizability and external validity of the existing evidence.

We recognise several limitations. To reduce variability and enhance scientific validity, we limited study inclusion to healthy participants; however, among healthy people there are natural hormonal fluctuations, such as menstrual cycle or menopause and testosterone fluctuations [63–65], that are expected to introduce variability in sleep parameters. While several studies included in this review noted the use of contraceptives in their female participants [18, 28, 40, 41] and a few shared relevant information about participants’ menstrual cycle [17, 19, 26–28, 35], none investigated the role of sex steroid hormones in sleep wave parameters. The absence of this consideration is crucial as it can disrupt sex-specific findings leading to an over-simplification of the study of biological sex differences, which does not allow for discussion concerning sexual dimorphism or lack of thereof [6].

Likewise, confounding influences from other variables, including gender, stress, diet, physical activity, sleep environment, among others [66–69], may also influence sleep parameters and reported results on sex differences, but such variables were not investigated in the studies included in this review. Another factor to consider is age. While age was considered in the studies, participant age ranges spanned many years, thus requiring us to use broad developmental categories to analyze results without sacrificing significant data points. We emphasise that future studies should investigate sex differences in sleep wave parameters with greater precision in age, as sex-specific physiology is age-dependent. The integration of advanced statistical techniques and machine learning [70, 71] could lead to a more precise identification of sex-specific sleep wave parameters, should they exist,  which is hardly feasible through traditional hypothesis-driven analytic methodologies that assume linearity in the relationship between variables.

Additional limitations include heterogeneity and variability in recording methods and definitions of wave band frequencies (Table 2) among the studies included in this review. Small sample sizes and sex distribution imbalances in some studies (Table 1) may have limited the statistical power to detect sex differences.

It is noteworthy that our searchers were limited to studies published in English, leading to a possible exclusion of evidence published in other languages, and thereby affecting the generalizability of results to the global community. To minimize this bias, we searched Embase, which indexes English language publications from Europe and Asia, through which we retrieved relevant publications coming from these regions.

To complete our discussion, we follow with recommendations for future research on the topic of sex differences in sleep wave parameters. The variability in data recording and processing (i.e., manual versus automated), the number of electrodes, and the leads from which the data were recorded may have contributed to variation observed in our evidence synthesis. Even across studies with the same recording protocol (i.e., PSG with EEG or standalone EEG) researchers defined frequency bands differently and performed data manipulations in the analyses of wave parameters. While it is understandable that different approaches to data processing would be required based on the research objective, to report raw/unprocessed data by sex in the future can guide a richer, more nuanced understanding of brain activity in sleep powered by advanced signal processing, machine learning, and deep learning techniques. A consensus between researchers across health pillars [72] on developing best practices to study sex difference in sleep parameters while considering social and environmental factors is of utmost importance to avoid oversimplification of sex effects, promote inclusivity in research, and improve the generalizability of research results.

## Conclusions

Sleep research to date has not been successful in producing consistent results on sex differences in sleep wave parameters. We found that high certainty exists in evidence reporting no sex difference in alpha and delta relative power [35, 43]. Among the few findings of moderate certainty reporting sex differences are those that suggest a greater delta slope in female participants [28, 29] and greater normalized delta power in males [5, 35]. All other results were of low or very low certainty. It is possible that the reported results in the studies included in this review reflect unmeasured social parameters, instead of biological sex. Considering that methodological variability may influence findings of studies included in this review, all efforts should be made to standardize and uniform testing procedures across research settings.

## Methods

### Protocol and registration

We registered the protocol for this systematic review on the prospective register of systematic reviews (PROSPERO) of the Centre for Reviews and Dissemination (Supplementary Material S5). We followed the reporting guideline of the Preferred Reporting Items for Systematic Reviews and Meta-Analyses (PRISMA) to conduct the review (Supplementary Material S6).

### Data search and sources

#### Search strategy

We developed a search strategy (Supplementary Material S7) in collaboration with an information specialist (JB) at a large rehabilitation research-teaching hospital. The search strategy used a mix of keywords and subject headings (e.g. MeSH, Emtree) combined using the Boolean operators AND and OR, and applied the following four concepts: (A) sex differences, (B) EEGs, (C) electrophysiological markers and (D) sleep. Search terms for concept A were sourced from a previous review and used with modifications [73]. When possible, we applied limits to focus on human studies and to exclude conference proceedings. Results were limited to English language publications. No date limits were used.

We searched MEDLINE ALL (Ovid), Embase Classic and Embase (Ovid), APA PsycInfo (Ovid), and Scopus from each database’s inception on November 22, 2021. A supplemental search was conducted in Dissertations and These Global (Proquest) to review relevant dissertations or theses reference lists. We repeated searches on December 11, 2024. We exported results from each database into Endnote and were subsequently imported into the Systematic Review Accelerator’s Deduplicator for duplicate removal before the screening stage. We cross-checked the references list of all included studies.

#### Eligibility criteria based on the PICOS approach

We defined eligibility criteria for study inclusion a priori, using the PICOS approach:

**P** (Participants): human participants of all ages who at the time of participation in research were considered healthy, defined as a person who is not treated with medications that cross the blood–brain barrier or diagnosed with any form of illness.

**I** (Interventions): this element was not applicable as this was a systematic review of observational studies.

**C** (Comparisons): sex differences in reported outcomes.

**O** (Outcomes): sleep wave parameters as reported by researchers.

**S** (Study design): observational studies of any study design (i.e., cohort, cross-sectional, case control, case series) focusing on sex differences in sleep wave parameters, as presented in study objectives and/or hypothesis.

We excluded non-human studies, conference proceedings, case reports, commentaries, reviews, dissertations, and book chapters.

#### Inclusion and exclusion criteria

We included peer-reviewed studies published in English language that reported on sex differences in sleep wave parameters, including indices derived from these parameters. We excluded studies that focused on a different but concurrent topic in sleep wave (e.g. sleep stage distribution, % sleep stage, and wave frequency, duration, incidence, periodicity, etc.) or had reported sex differences in response to intervention [74–78]. We considered baseline data or data from untreated arm (i.e., control arm) in intervention studies reporting on sex differences in sleep wave parameters in healthy persons. Case reports dissertations and studies with no primary data were excluded.

### Data collection and analysis

#### Selection process

Two review authors (CCKL and TM or TTS and TM) conducted the title/abstract screening. Six review authors (CCKL, RC, SN, FF, TTS, and TM) performed full text review of potentially relevant studies. Studies that did not meet the inclusion criteria were excluded. The senior author reviewed the quality of the first and second levels of screening.

Two review authors (CCKL and RC) edited the data extraction form used by Mollayeva et al. [79] to include PROGRESS-Plus parameters. Five review authors (CCKL, RC, SN, FF, TTS) used the form to extract data from eligible studies. The form included: (i) study identifiers (i.e., author names, publication year, setting, country); (ii) study characterizations (i.e., research objective(s), sample size, inclusion and exclusion criteria using the PROGRESS-Plus lens, etc.); participant characteristics (i.e., mean age and standard deviation, sex, collected/reported PROGRESS-plus characteristics); research study characteristics (i.e., study design, studied wave parameters [frequency, power, density, amplitude, etc.], statistical analysis); and results pertinent to our research objectives. When information was unclear, we contacted study authors to elaborate on the results and provide further details [32, 43].

Six review authors (CCKL, RC, SN, FF, TTS and TM) checked the accuracy of data extraction. We resolved inconsistencies through group discussion.

#### Synthesis methods

Multiple authors (RC, CCKL, SN, FF, TTS and TM) compiled study characteristics into a table to assess similarities between studies across PICOS criteria, which was then used in further data synthesis by the team. We categorized data by wave frequency or morphology (delta, theta, alpha, sigma, beta, gamma; spindle, sawtooth), investigated parameters (amplitude, connectivity, density, power, etc.), and measured units (e.g., Hz, $\mu {V}^{2}/s$, $\mu {V}^{2}$, etc.). Due to variability in age ranges of participants across the studies, we grouped the age-related developmental stages defined by Lally and Valentine-French into fewer categories to facilitate comparison of results [80]. Lally and Valentine-French define the developmental stages as: prenatal (before birth); infancy and toddlerhood (from birth to age 2); early childhood (ages 2–6); middle and late childhood (age 6 to the onset of puberty); adolescence (from the onset of puberty until age 18); emerging adulthood (ages 18–25); early adulthood (ages 25 to 40–45); middle adulthood (ages 40–45 to 60–65); and late adulthood (65 onward) [80]. For our analysis, we combined them into five categories: infancy and toddlerhood (from birth to < age 2), early, middle, and late childhood and adolescence (ages 2 to < 18); emerging and early adulthood (ages 18 to < 45); middle adulthood (ages 45 to < 65); and late adulthood (65 onwards) [80]. We conducted subgroup analyses by frequency wave, sleep wave parameter, and study quality to explore whether results on sex differences varied.

We observed heterogeneity across all PICOS criteria, confirming that the assumptions for conducting a classical meta-analysis were not met [74]. Further, variations in EEG recording techniques, definitions sleep wave parameters, and differences in the study samples and characteristics prevented us from performing data conversions. Therefore, we synthesized data descriptively, using approach proposed by Slavin [75], and denoted differences between the sexes as follows: higher values in males; higher values in females; and for no difference. We used statistical significance of results as reported by study authors. We created figures to summarize the results for each study by age (treated as a continuous variable) and age-related developmental stage to facilitate more refined comparisons and capture any distinct patterns in sex differences across life stages.

#### Assessment of risk bias in included studies and certainty assessment

We assessed risk of bias in the included studies using the tool developed by the National Institutes of Health (NIH) for observational cohort and cross-sectional studies [76]. The tool addresses the presence of six potential sources of bias within (1) study participation, (2) study attrition (for cohort studies), (3) association bias, (4) outcome measurement bias, (5) confounding and statistical analysis and (6) reporting [78]. The senior author (TM) conducted a training and calibration session on the quality assessment procedure with four review authors (TTS, RC, SN, and FF) on eight included studies, following which the three review authors (TTS, SN, and FF) together with the senior author (TM) applied the tool to each included study, and recorded supporting information for judgements of risk of bias for each domain. In addition to the crude calculation of the number of biases present out of total number of possible, we rated the study quality as follows: (i) excellent (“ +  + ”) when all or most of the criteria were fulfilled (i.e. allowing at most one ‘cannot determine’ or ‘not reported’); (ii) good (“ + ”) when half of the criteria were fulfilled; (iii) fair (“−”) when less than half of the criteria were fulfilled (Supplementary Material S4). We did not exclude studies based on the quality assessment, but considered each study’s quality in the data analysis, reporting, and interpretation.

The GRADE system downgrades the evidence from intervention studies by evaluating the extent of study limitations, including risk of bias, inconsistency of associations, imprecision, indirectness, and publication bias. The certainty of evidence in observational studies included in this review was completed qualitatively, following study quality assessment (e.g., excellent, good, and fair) based on the risk of bias assessment across six domains. We assessed the certainty of the evidence as high if two or more excellent quality studies coming from different team of investigators were concordant regarding the observed sex differences in a specific sleep parameter of the same wave band or waveform and no discordant results from studies of equal quality were present. We assessed certainty of evidence as moderate if two or more studies of good and/or excellent quality were concordant in their results, with a maximum of one discordant result in studies of good or excellent quality. We assigned low certainty if at least two fair and/or good quality studies were concordant in results, with a maximum of one discordant result in studies of fair or good quality. In all other situations, we assessed the certainty as very low.

#### Sensitivity analysis

We conducted sensitivity analyses to examine the robustness of findings. We visually positioned results on the lifespan continuum, to evaluate the consistency of the results among the same age-related developmental stages and reporting EEG parameters, and to see results of each study in reference to all other studies. Finally, we conducted subgroup analyses based on risk of bias assessment and the type and number of PROGRESS-Plus covariates considered in data analyses. This allowed us to evaluate the impact of study quality and participants’ characteristics on the consistency of the results.

### Publication bias

Due to the limited number of studies reporting on the same EEG parameter, high heterogeneity in terms of study design, population, and definition of sleep wave parameters and frequency bands (Tables 1, 2), we did not perform evaluation of the publication bias using statistical tests [77]. We applied visual inspection to handle data issues and certainty assessment to provide valuable insights on the state of evidence on the topic [75].

### Dealing with missing data

We contacted study authors to verify key study characteristics and obtain missing data. We received responses from two authors [32, 43] on the requested information.

### Ethical review

We did not seek ethical approval, as this study did not involve primary data collection.

## Supplementary Information

Supplementary material 1.

Supplementary material 2.

Supplementary material 3.

Supplementary material 4.

Supplementary material 5.

Supplementary material 6.

Supplementary material 7.

## Data Availability

No datasets were generated or analysed during the current study.

## Abbreviations

APA: American Psychology Association
CRD: Centre for Reviews and Dissemination
CNS: Central nervous system
EEG: Electroencephalography
Hz: Hertz
NREM: Non-rapid eye movement
REM: Rapid eye movement
NIH: National Institutes of Health
PROGRESS-Plus: Place of residence, Race/ethnicity/culture/language, Occupation, Gender/ Sex, Religion, Education, Socioeconomic status, Social capital, Other
PROSPERO: International Prospective Register of Systematic Reviews
PRISMA: Preferred Reporting Items for Systematic Reviews and Meta-Analyses
MeSH: Medical subject headings
SD: Standard deviation
SWS: Slow-wave sleep
ISO: Infra-slow oscillations
HFO: High-frequency oscillations
RCT: Randomized controlled trials
