# Supplementary material for: Sex differences in electrical activity of the brain during sleep: a systematic review of electroencephalographic findings across the human lifespan
Source: Biomed Eng Online. 2025 Mar 12;24:33. doi: 10.1186/s12938-025-01354-z (PMC11899717; doi:10.1186/s12938-025-01354-z)
Supplement: Supplementary file 1 — Supplementary material 1. [file 12938_2025_1354_MOESM1_ESM.docx]

**Supplementary Material S1.** Excluded studies and reason for exclusion

**Report not retrieved:** Article requested on Research Gate on February 28, 2021, but could not be obtained

Armitage, R. & Smith, Carlyle & Thompson, S. & Hoffmann, R.. (2001). Sex differences in slow-wave activity in response to sleep deprivation. Sleep Research Online. 4. 33-41.

| **#** | **Citation** | **Reason for Exclusion** |
| --- | --- | --- |
| 1 | Åkerstedt T, Lekander M, Nilsonne G, Tamm S, D'onofrio P, Kecklund G, Fischer H, Schwarz J. Effects of late-night short-sleep on in-home polysomnography: relation to adult age and sex. J Sleep Res. 2018 Aug;27(4):e12626. doi: 10.1111/jsr.12626. Epub 2017 Oct 30. PMID: 29082633. | Lack of relevant data: Only looking at REM, N1, N2, N3, etc. No direct comparison between sexes |
| 2 | Antonijevic IA, Murck H, Frieboes R, Holsboer F, Steiger A. On the gender differences in sleep-endocrine regulation in young normal humans. Neuroendocrinology. 1999 Oct;70(4):280-7. doi: 10.1159/000054487. PMID: 10529623. | Intervention w/out control: Intervention (GHRH and isotonic saline), separate data for control not shared |
| 3 | Baena D, Toor B, Ray LB, Smith D, Kong P, Lopez J, Hoffmann R, Bertram H, Robillard R, Armitage R, Fogel SM. Sleep spindles in adolescents with major depressive disorder. J Affect Disord. 2024 Jan 1;344:535-545. doi: 10.1016/j.jad.2023.10.039. Epub 2023 Oct 10. PMID: 37827259. | Lack of relevant data: Sex-specific data of healthy controls not presented on sleep spindles in numeric or visual form |
| 4 | Bódizs R, Gombos F, Ujma PP, Kovács I. Sleep spindling and fluid intelligence across adolescent development: sex matters. Front Hum Neurosci. 2014 Nov 28;8:952. doi: 10.3389/fnhum.2014.00952. PMID: 25506322; PMCID: PMC4246682. | Lack of relevant data: Data not obtained after contacting study author |
| 5 | Campbell IG, Figueroa JG, Bottom VB, Cruz-Basilio A, Zhang ZY, Grimm KJ. Maturational trend of daytime sleep propensity in adolescents. Sleep. 2024 Jan;47(1):zsad263. doi: 10.1093/sleep/zsad263. | Lack of relevant data: No sex-specific objective or hypothesis; focus on MSLT |
| 6 | Castro JS, Leslie ATFS, Guinsburg R. Perinatal factors associated with amplitude-integrated electroencephalography abnormalities in preterm infants on the first day of life. J Pediatr (Rio J). 2020 Sep-Oct;96(5):644-651. doi: 10.1016/j.jped.2019.06.004. Epub 2019 Jul 17. PMID: 31325413; PMCID: PMC9432229. | Lack of relevant study population: We considered preterm babies (between 23 and 32 weeks) as not fully developed, thus not meeting the healthy study population criteria |
| 7 | Chellappa SL, Steiner R, Oelhafen P, Cajochen C. Sex differences in light sensitivity impact on brightness perception, vigilant attention and sleep in humans. Sci Rep. 2017 Oct 27;7(1):14215. doi: 10.1038/s41598-017-13973-1. PMID: 29079823; PMCID: PMC5660221. | Intervention w/out control: Intervention, light exposure. No data available that is not related to light exposure |
| 8 | Choi H, Jeong J, Kim H, Shin C, Yoon IY. Implication of Fast Activities of Spectral Analysis in Subjective Sleep Complaints of Elderly Women. J Geriatr Psychiatry Neurol. 2019 Jan;32(1):24-30. doi: 10.1177/0891988718813711. Epub 2018 Nov 26. PMID: 30477382. | Lack of relevant data: Data for participants who were not depressed according to GDS score were not presented separately from those who were depressed, and inclusion criteria allowed for participation of people taking medications that cross blood-brain barrier |
| 9 | Chylinski D, Narbutas J, Balteau E, Collette F, Bastin C, Berthomier C, Salmon E, Maquet P, Carrier J, Phillips C, Lina JM, Vandewalle G, Van Egroo M. Frontal grey matter microstructure is associated with sleep slow waves characteristics in late midlife. Sleep. 2022 Nov 9;45(11):zsac178. doi: 10.1093/sleep/zsac178. PMID: 35869626; PMCID: PMC9644125. | Lack of relevant data: Sex not primary focus of the study |
| 10 | Corsi-Cabrera M, Sánchez AI, del-Río-Portilla Y, Villanueva Y, Pérez-Garci E. Effect of 38 h of total sleep deprivation on the waking EEG in women: sex differences. Int J Psychophysiol. 2003 Nov;50(3):213-24. doi: 10.1016/s0167-8760(03)00168-5. PMID: 14585490. | Lack of relevant data: No numerical wave data available, and the only table that compares males and females is with eyes open and during vigilance task performance |
| 11 | Darchia N, Campbell IG, Basishvili T, Eliozishvili M, Tchintcharauli T, Oniani N, Sakhelashvili I, Feinberg I. Sleep electroencephalogram evidence of delayed brain maturation in attention deficit hyperactivity disorder: a longitudinal study. Sleep. 2022 Sep 8;45(9):zsac163. doi: 10.1093/sleep/zsac163. PMID: 35866992. | Lack of relevant data: Sex-specific data not presented |
| 12 | Emegbo S. Age and sex differences in human sleep: Objective versus self-reported measures in healthy adults. [thesis]. Guildford: University of Surrey; 2012. | Wrong study design: Thesis |
| 13 | Eriksson MH, Baldeweg T, Pressler R, Boyd SG, Huber R, Cross JH, Bölsterli BK, Chan SYS. Sleep homeostasis, seizures, and cognition in children with focal epilepsy. Dev Med Child Neurol. 2023 May;65(5):701-711. doi: 10.1111/dmcn.15403. Epub 2022 Sep 7. PMID: 36069073. | Lack of relevant data: Sex-specific data not presented |
| 14 | Gaillard JM, Blois R. Spindle density in sleep of normal subjects. Sleep. 1981;4(4):385-91. doi: 10.1093/sleep/4.4.385. PMID: 7313391. | Lack of relevant data: Does not include any wavelength categories or specific data, more like a review |
| 15 | Gaudreault PO, Lina JM, Descoteaux M, Gosselin N, Doyon J, Deslauriers-Gauthier S, Carrier J. The length of the thalamo-cortical white matter fibers brings insight into sex differences in sleep spindle frequency. bioRxiv. 2022 May 11;491489. doi: 10.1101/2022.05.11.491489. | Unpublished manuscript: DOI is linked to a pre-print from 2022 that does not seem to have been published |
| 16 | Greenlund IM, Smoot CA, Carter JR. Sex differences in blood pressure responsiveness to spontaneous K-complexes during stage II sleep. J Appl Physiol (1985). 2021 Feb 1;130(2):491-497. doi: 10.1152/japplphysiol.00825.2020. Epub 2020 Dec 10. PMID: 33300855; PMCID: PMC7948112. | Lack of relevant data: Different research question, relates to blood pressure and K–complexes |
| 17 | Hein M, Lanquart JP, Loas G, Hubain P, Linkowski P. Alterations of neural network organization during REM sleep in women: implication for sex differences in vulnerability to mood disorders. Biol Sex Differ. 2020 Apr 25;11(1):22. doi: 10.1186/s13293-020-00297-5. PMID: 32334638; PMCID: PMC7183628. | Lack of relevant data: Data for participants who were not depressed according to BDI score were not presented separately from those who were depressed |
| 18 | Höller Y, Eyjólfsdóttir SG, Rusiňák M, Guðmundsson LS, Trinka E. Movement Termination of Slow-Wave Sleep-A Potential Biomarker? Brain Sci. 2024 May 13;14(5):493. doi: 10.3390/brainsci14050493. PMID: 38790471; PMCID: PMC11120257. | Lack of relevant data: No healthy control data presented |
| 19 | Iwagami M, Seol J, Hiei T, Tani A, Chiba S, Kanbayashi T, Kondo H, Tanaka T, Yanagisawa M. Association between electroencephalogram-based sleep characteristics and physical health in the general adult population. Sci Rep. 2023 Dec 8;13(1):21545. doi: 10.1038/s41598-023-47979-9. PMID: 38066043; PMCID: PMC10709300. | Lack of relevant data: Different focus – clinical sleep markers (i.e., total sleep time [TST], sleep efciency, sleep onset latency, N1%, N2%, N3%, REM%, wake after sleep onset |
| 20 | Kiss O, Goldstone A, de Zambotti M, Yüksel D, Hasler BP, Franzen PL, Brown SA, De Bellis MD, Nagel BJ, Nooner KB, Tapert SF, Colrain IM, Clark DB, Baker FC. Effects of emerging alcohol use on developmental trajectories of functional sleep measures in adolescents. Sleep. 2023 Sep 8;46(9):zsad113. doi: 10.1093/sleep/zsad113. PMID: 37058610; PMCID: PMC10848227. | Lack of relevant study population: Moderate-heavy drinking considered as unhealthy; participants transitioned between no/low and moderate/heavy categories. Data for participants who remained strictly in the no/low drinking category (healthy) throughout the study not provided separately. |
| 21 | Kizilkilic EK, Karadeniz D, Senel GB. Attention and executive function impairments in obstructive sleep apnea are associated with decreased sleep spindles. Acta Neurol Belg. 2024 Oct;124(5):1507-1515. doi: 10.1007/s13760-024-02534-9. Epub 2024 Apr 2. PMID: 38563875. | Lack of relevant data: Sex-specific data not presented for healthy controls |
| 22 | Messman BA, Wiley JF, Yap Y, Tung YC, Almeida IM, Dietch JR, Taylor DJ, Slavish DC. How much does sleep vary from night-to-night? A quantitative summary of intraindividual variability in sleep by age, gender, and racial/ethnic identity across eight-pooled datasets. J Sleep Res. 2022 Dec;31(6):e13680. doi: 10.1111/jsr.13680. Epub 2022 Jul 10. PMID: 35811092; PMCID: PMC9649840. | Lack of relevant data: Different focus on sleep duration and efficiency |
| 23 | Mongrain V, Carrier J, Dumont M. Difference in sleep regulation between morning and evening circadian types as indexed by antero-posterior analyses of the sleep EEG. Eur J Neurosci. 2006 Jan;23(2):497-504. doi: 10.1111/j.1460-9568.2005.04561.x. PMID: 16420456. | Lack of relevant data: The paper doesn’t share sex differences or data for the sexes evaluated for |
| 24 | Oh CH, Wallace ML, Germain A. Childhood trauma and gender: Synergistic and additive effects on sleep in healthy young adults. Sleep Health. 2022 Oct;8(5):498-504. doi: 10.1016/j.sleh.2022.06.008. Epub 2022 Aug 12. PMID: 35965190; PMCID: PMC10262693. | Lack of relevant data: Reported delta sleep percentage in model |
| 25 | Poirson B, Vandel P, Bourdin H, Galli S. Age-related changes in sleep spindle characteristics in individuals over 75 years of age: a retrospective and comparative study. BMC Geriatr. 2024 Sep 20;24(1):778. doi: 10.1186/s12877-024-05364-9. PMID: 39304816; PMCID: PMC11414178. | Lack of relevant study population: Did not meet inclusion criteria based on number of disorders in study population |
| 26 | Ricci A, He F, Calhoun SL, Fang J, Vgontzas AN, Liao D, Bixler EO, Younes M, Fernandez-Mendoza J. Sex and Pubertal Differences in the Maturational Trajectories of Sleep Spindles in the Transition from Childhood to Adolescence: A Population-Based Study. eNeuro. 2021 Jul 14;8(4):ENEURO.0257-21.2021. doi: 10.1523/ENEURO.0257-21.2021. PMID: 34168053; PMCID: PMC8281264. | Lack of relevant study population: Did not state that study population was healthy and controlled for psychiatric/learning disorder in multivariate modelling |
| 27 | Sanchez E, El-Khatib H, Arbour C, Bedetti C, Blais H, Marcotte K, Baril AA, Descoteaux M, Gilbert D, Carrier J, Gosselin N. Brain white matter damage and its association with neuronal synchrony during sleep. Brain. 2019 Mar 1;142(3):674-687. doi: 10.1093/brain/awy348. PMID: 30698667; PMCID: PMC6391600. | Lack of relevant data: Sex-specific data not presented for healthy controls |
| 28 | Sun H, Ye E, Paixao L, Ganglberger W, Chu CJ, Zhang C, Rosand J, Mignot E, Cash SS, Gozal D, Thomas RJ, Westover MB. The sleep and wake electroencephalogram over the lifespan. Neurobiol Aging. 2023 Apr;124:60-70. doi: 10.1016/j.neurobiolaging.2023.01.006. Epub 2023 Jan 19. PMID: 36739622; PMCID: PMC9957961. | Lack of relevant data: No sex-specific objective or hypothesis |
| 29 | Talukder A, Yeung D, Li Y, Anandanadarajah N, Umbach DM, Fan Z, Li L. Comparison of power spectra from overnight electroencephalography between patients with Down syndrome and matched control subjects. J Sleep Res. 2024 Oct;33(5):e14187. doi: 10.1111/jsr.14187. Epub 2024 Feb 27. PMID: 38410055; PMCID: PMC11347723. | Lack of relevant data: Sex-specific data not presented for healthy controls |
| 30 | Ueda R, Takeichi H, Kaga Y, Oguri M, Saito Y, Nakagawa E, Maegaki Y, Inagaki M. Atypical gamma functional connectivity pattern during light sleep in children with attention deficit hyperactivity disorder. Brain Dev. 2020 Feb;42(2):129-139. doi: 10.1016/j.braindev.2019.11.001. Epub 2019 Nov 22. PMID: 31761311. | Lack of relevant data: Sex-specific data not presented for healthy controls |
| 31 | Ujma PP, Konrad BN, Simor P, Gombos F, Körmendi J, Steiger A, Dresler M, Bódizs R. Sleep EEG functional connectivity varies with age and sex, but not general intelligence. Neurobiol Aging. 2019 Jun;78:87-97. doi: 10.1016/j.neurobiolaging.2019.02.007. Epub 2019 Feb 21. PMID: 30884412. | Lack of relevant data: The paper looks at sleep EEG in relation to intelligence and there is no data provided on sleep EEG irrespectively |
| 32 | Ujma PP, Simor P, Steiger A, Dresler M, Bódizs R. Individual slow-wave morphology is a marker of aging. Neurobiol Aging. 2019 Aug;80:71-82. doi: 10.1016/j.neurobiolaging.2019.04.002. Epub 2019 Apr 16. PMID: 31103634. | Lack of relevant data: Sex not primary focus of the study |
| 33 | Valomon A, Riedner BA, Jones SG, Nakamura KP, Tononi G, Plante DT, Benca RM, Boly M. A high-density electroencephalography study reveals abnormal sleep homeostasis in patients with rapid eye movement sleep behavior disorder. Sci Rep. 2021 Feb 26;11(1):4758. doi: 10.1038/s41598-021-83980-w. PMID: 33637812; PMCID: PMC7910582. | Lack of relevant data: Sex-specific data not presented for healthy controls |
| 34 | van Putten MJAM, Olbrich S, Arns M. Predicting sex from brain rhythms with deep learning. Sci Rep. 2018 Feb 15;8(1):3069. doi: 10.1038/s41598-018-21495-7. PMID: 29449649; PMCID: PMC5814426. | Lack of relevant data: EEG was done on awake patients |
| 35 | Zeng G, Zhou Y, Yang Y, Ruan L, Tan L, Luo H, Ruan J. Neural oscillations after acute large artery atherosclerotic cerebral infarction during resting state and sleep spindles. J Sleep Res. 2023 Oct;32(5):e13889. doi: 10.1111/jsr.13889. Epub 2023 Mar 21. PMID: 36944554. | Lack of relevant data: Sex-specific data not presented for healthy controls |
