# Supplementary material for: Sex differences in electrical activity of the brain during sleep: a systematic review of electroencephalographic findings across the human lifespan
Source: Biomed Eng Online. 2025 Mar 12;24:33. doi: 10.1186/s12938-025-01354-z (PMC11899717; doi:10.1186/s12938-025-01354-z)
Supplement: Supplementary file 2 — Supplementary material 2. [file 12938_2025_1354_MOESM2_ESM.docx]

**Supplementary Material S2.** Study and sample characteristics, including inclusion/exclusion criteria and consideration of PROGRESS-Plus parameters.

| Author (year); Journal | **Research equity consideration**   1. Country; region; city; location of research 2. Study objective 3. PROGRESS-Plus consideration in research design 4. Source of informed consent/language of consent 5. Attrition description | **Inclusion criteria \| Justification**   1. Participants’ social characteristics 2. Participants’ clinical characteristics 3. Participants’ behavior 4. Other parameters | **Exclusion criteria \| Justification**   1. Participants’ social characteristics 2. Participants’ clinical characteristics 3. Participants’ behavior 4. Other parameters | **PROGRESS-Plus equity reporting**  (1) Place of residence of research participants  (2) Race/ethnicity/culture/language  (3) Occupation  (4) Gender/Sex (%M)  (5) Religion  (6) Education  (7) Socioeconomic status  (8) Social capital  (9) Plus parameters | **Research notes on PROGRESS-Plus** |
| --- | --- | --- | --- | --- | --- |
| Armitage R. (1995); *Sleep.* | 1. USA; Texas, Dallas; University of Texas; Southwestern Medical Center Sleep Study Unit 2. Analyze EEG parameters and interhemispheric variances by sleep stages and sex 3. Research concerns sex diff 4. NR / NR 5. NA | 1. NR 2. No meds except oral/implant CCs **\|** NR 3. a. Pre-study alc & caff restriction **\|** NR 4. Keep sleep-wake pattern for 5 d **\|** NR 5. NR | 1. NR 2. NR 3. NR 4. First-degree relatives w psy d/o **\|** NR | 1. NR 2. NR 3. NR 4. 11M, 11F (50%)* 5. NR 6. NR 7. NR 8. NR 9. **Age:** mean 25.6±4.2 yrs (M), 24.8±3.9 yrs (F) | 1. Sex plays little role in moderating EEG frequency |
| Armitage R. et al. (2000); *Sleep.* | 1. USA; Texas, Dallas; University of Texas; Southwestern Medical Center Sleep Study Unit 2. Compare δ activity in NREM btw depressed and healthy pcps by sex 3. Research concerns sex diff 4. NR / NR 5. NA | 1. NR 2. NR 3. 2 continuous d of sleep w/o disruption **\|** NR 4. No family hx of axis 1 d/o **\|** NR | 1. Shift worker **\|** NR 2. Current axis I/sleep d/o **\|** NR 3. Substance abuse w/i 12 months pre-study **\|** NR 4. NR | 1. NR 2. NR 3. NR 4. 15M, 8F† (65%)* 5. NR 6. NR 7. NR 8. NR 9. **Age:** range 22-40 yrs; mean 27.1±5.9 yrs (M), 30.9±6.2 yrs (F)* | 1. Sex diff in δ activity in healthy adults are likely to decline with ↑ age. 2. Influence of age × δ activity varied as a function of both psychiatric status & sex. |
| Baker F. C. et al. (2012); *J Sleep Res.* | 1. Australia; Victoria, Abbotsford; Compumedics 2. Explore regional and sex diff in sleep and EEG changes in adolescents over 6–8 months 3. Research concerns sex diff and age 4. Given by parents and assented by participant / NR 5. 1 F and 1 M had contaminated data, 1 M had extremely poor sleep | 1. Adolescents 11-14 yrs **\|** Sleep architecture changes during adolescence 2. NR 3. Keep sleep-wake pattern for 5 d **\|** NR 4. NR | 1. NR 2. a. CNS d/o 3. Lifetime psy/neuro/sleep d/o **\|** NR 4. Loss of consciousness >30 min **\|** NR 5. NR 6. NR | 1. Victoria, Abbotsford ‡ 2. NR 3. NR 4. 18M, 15F (55%)* 5. NR 6. NR 7. NR 8. NR 9. a. **Age:** range 11-14 yrs; mean 12.7±0.9 yrs (M), 12.4±0.7 yrs (F)* 10. **Tanner stage**: median 2 (M), 3 (F) | 1. Significant sex main effect on δ activity 2. Adolescence is a period of significant change in EEG frequencies |
| Campbell I. G. et al. (2005); *Sleep.* | 1. USA; California, Davis; Subject’s home 2. Compare adolescent δ decline in EEG data from 9- and 12-year-old M and F 3. Research concerns sex diff and age 4. Given by parent and assented by participant 5. NA | 1. NR 2. NR 3. Keep sleep-wake pattern for 5 d **\|** NR 4. NR | 1. NR 2. a. Hx of head injury 3. Lifetime psy/neuro d/o **\|** NR 4. NR 5. NR | 1. NR 2. NR 3. NR 4. 35M, 35F (50%)* 5. NR 6. NR 7. NR 8. NR 9. a.  **Age:** 1^st^ recording mean 9.31±0.04 yrs (C9), 12.33±0.04 yrs (C12); 2^nd^ recording mean 9.83±0.05 yrs (C9), 12.80±0.04 yrs (C12)* 10. **Tanner stage:** 1^st^ recording mean 1.32±0.10 (C9), 2.66±0.22 (C12), 2^nd^ recording mean 1.31±0.11 (C9), 2.89±0.22 (C12) | 1. Sex diff was seen in slow-wave EEG in C12 F cohort implying adolescent brain maturation may occur faster in F than M 2. Age was a predictor of sleep schedules in both recordings |
| Campbell I. G. et al. (2012); *PNAS.* | 1. USA; California, Davis; Subject’s home 2. Study the timing of NREM δ EEG power decline and pubertal maturation 3. Research concerns sex diff and age 4. NR / NR 5. Pcps included if they completed at least 3 yrs of study | 1. NR 2. NR 3. Keep sleep-wake pattern for 5 d/no naps **\|** NR 4. NR | 1. NR 2. Current psy/neuro d/o **\|** NR 3. Sleep disturbance **\|** NR 4. NR | 1. University town near University of California Davis 2. 79% non-Hispanic white, 7% Asian, 5% Hispanic, 3% African American, 7% mixed race 3. NR 4. C9: 15M, 15F (50%);  C12: 18M, 19F (49%)* 5. NR 6. NR 7. NR 8. NR 9. a. **Age:** 9 and 12 yrs* 10. **Tanner stage:** data reported visually (Fig. 2)* | 1. Clear relationship btw age of rapid pubertal maturation and delta power decline 2. ↑ age was associated with less time asleep, ↓ sleep efficiency 3. Sample demographic may not be representative of society |
| Carrier J., et al. (2001); *Psychophysiology.* | 1. USA; Pennsylvania; Pittsburgh; Western Psychiatric Institute and Clinic; Sleep and Chronobiology Center 2. Investigating the impact of age and gender on sleep EEG power spectral density in middle-aged pcps 3. Research concerns sex and gender diff, and age 4. Given by participant / NR 5. NA | 1. NR 2. Healthy **\|** NR 3. NR 4. No history of personal/family psy/neuro/sleep/medical d/o **\|** NR | 1. NR 2. a. Any medical condition 3. On sleep meds **\|** NR 4. Obese (BMI >27)/considerable wt loss **\|** NR 5. AI > 10 **\|** NR 6. PLMAI > 10 **\|** NR 7. No alc/drugs ≥14 d pre-study **\|** NR 8. First-degree relative with prev psy d/o or seizures **\|** NR | 1. NR 2. NR 3. >95% were students, workers, or homemakers 4. 53M, 47F (53%)* 5. NR 6. NR 7. NR 8. NR 9. **Age:** range 20-60 yrs* | 1. Gender effects differed across freq bins, but no sign interactions were found btw age and gender, suggesting that the aging process does not differently affect M and F btw 20 and 60 years old 2. Age had a significant role on sleep EEG spectral power density |
| Carrier J., et al. (2011); *Eur. J. Neurosci.* | 1. Canada; Quebec; Montreal; Unviersité Laval; Centre d’étude du sommeil et des rythmes biologiques 2. Study how age, sex, and topography impact SW characteristics in healthy young and middle-aged pcps 3. Research concerns sex diff and age 4. Given by participant / NR 5. NA | 1. 2 age groups: 20-30 yrs and 41-60 yrs **\|** NR 2. <4 on BDS **\|** NR 3. NR 4. Had PSG screen **\|** NR | 1. Night work **\|** NR 2. a. On sleep altering meds **\|** NR 3. Psy/neuro d/o hx **\|** NR 4. a. Smoking **\|** NR 5. Transmeridian travel 3 mos pre-study **\|** NR 6. Sleep disturbance+sleep duration of ≤7/≥9h **\|** NR 7. Perimenopausal **\|** NR 8. F’s using hormonal CCs or HRT **\|** NR | 1. NR 2. NR 3. NR 4. Young: 26M, 22F† (54%);  Middle-aged: 18M, 21F† (46%)* 5. NR 6. NR 7. NR 8. NR 9. **Age:** range 20-30 yrs (Young), 41-60 yrs (Middle-aged); mean 23.3±2.4 yrs (Young), 51.9±4.6 yrs (Middle-aged)* | 1. During adulthood, the characteristics of SWS are sign influenced by age and gender. 2. These findings may be important for understanding diff in sleep patterns for patients with psychopathological conditions |
| Dijk D. J. et al. (1989); *Sleep.* | 1. Netherlands; Groningen province; Groningen; University of Groningen; Department of Biological Psychiatry and Zoological Laboratory 2. Identify sex diff by comparing sleep in young adult and published sleep deprivation EEG data 3. Research concerns sex diff and age 4. NR / NR 5. NR | 1. NR 2. NR 3. No sleep complains **\|** NR 4. NR | 1. NR 2. NR 3. NR 4. NR | 1. NR 2. NR 3. NR 4. 13M, 15F (46%)* 5. NR 6. NR 7. NR 8. NR 9. **Age:** range 19-27 yrs; mean 23.5 yrs (M), 21.9 yrs (F) | 1. Sex diff weren’t just in δ freq but were present in nearly all analyzed freq bins 2. Sex diff in sleep are present in people’s 20s |
| Dorokhov B. V. et al. (2024);  *Chronobiol. Int.* | 1. Russia, Moscow, Institute of Higher Nervous Activity and Neurophysiology of Russian Academy of Sciences, Sleep Laboratory 2. Understand the paradoxical relationship between sex and objective and subjective sleep quality using EEG 3. Research concerns sex diff 4. Given by participant / NR 5. NA | 1. NR 2. NR 3. NR 4. NR | 1. a. ≤18 & ≥23 yrs **\|** NR 2. Shift worker **\|** NR 3. a. Hx of mental/sleep d/o **\|** NR 4. Currently experiencing mild cold **\|** NR 5. a. Complaints about poor phys condition & functioning **\|** NR 6. Missed class in the 2 wks pre-study 7. Crossed time zones 1 month pre-study **\|** NR 8. Irregular sleep-wake schedule (1+ h diff in wkday bedtime, frequent sleep reduction) 9. Pregnant/breastfeeding | 1. NR 2. NR 3. NR 4. 40M, 40F (50%)* 5. NR 6. University students 7. NR 8. NR 9. **Age:** mean 20.4±1.57 yrs (M), 20.25±1.14 yrs (F) | 1. Higher score on principle components of EEG, amp of NREM SWA, stronger sleep and wake drive in F |
| Feinberg I. et al. (2006); *Am J Physiol Regul Interg Comp Physiol.* | 1. USA; California; Davis; Subject’s home 2. Investigate the relationship btw δ decline, puberty, growth, sleep patterns, and age 3. Research concerns sex diff and age 4. NR / NR 5. NA | 1. NR 2. a. No psy/neuro/medical d/o **\|** NR 3. On meds affecting CNS **\|** NR 4. a. No sleep complaint **\|** NR 5. Keep sleep-wake pattern for 5 d/no naps **\|** NR 6. NR | 1. NR 2. NR 3. NR 4. First-degree relative w major psy d/o **\|** NR | 1. Davis community in California 2. 80% Caucasian, 7% Asian, 4% Hispanic. 2% African American 7% mixed race 3. NR 4. C9: 15M, 16F (48%);  C12: 19M, 19F (50%)* 5. NR 6. NR 7. NR 8. NR 9. a.  **Age:** 9 yrs±3 mos (C9), 12 yrs±3 mos (C12)* 10. **Tanner stages:** mean 1.3±0.1 (C9), 2.5±0.2 (C12)* | 1. There was no diff in the decline rate btw M and F 2. Comparing both age cohorts showed that F begins brain maturation before M 3. The ↓ in δ power density is strongly linked to both age and sexual maturation stage |
| Fukuda N. et al. (1999); *Psychiatry Clin. Neurosci.* | 1. Japan; Hokkaido; Sapporo; Subject’s homes 2. Investigate sex effects on SW activity in middle aged and elderly pcps by spectral analysis 3. Research concerns sex diff and age 4. NR / NR 5. NA | 1. NR 2. a. No sig medical history **\|** NR 3. No meds **\|** NR 4. NR 5. NR | 1. NR 2. NR 3. NR 4. NR | 1. NR 2. NR 3. NR 4. 8M, 8F (50%)* 5. NR 6. NR 7. NR 8. NR 9. **Age:** range 54-72 yrs; mean 61.5±4.66 yrs (M), 62.38±6.65 yrs (F) | 1. Sign gender diff exist in SWS. The generating mechanism of SWS in middle-aged and elderly F may still be conservative compared to M 2. No significant sex differences in visually scored EEG parameters |
| Hejazi S. N. et al. (2024); | 1. USA, MD, Bethesda, National Institute of Health Clinic Center, Sleep Laboratory 2. Examine relationship between sex, age, and sleep patterns with low and high δ pwr 3. Research concerns sex and age 4. Given by participant /NR 5. NA | 1. 18-65 yrs **\|** NR 2. a. No psychotropic meds for 2+ wks (5+ for fluoxetine & 3+ for aripiprazole) **\|** NR 3. Absence of Axis I d/o **\|** NR 4. NR 5. No first-degree relative w DMS-IV Axis I d/o **\|** NR | 1. NR 2. NR 3. NR 4. NR | 1. NR 2. NR 3. NR 4. 8M, 16F (33%)* 5. NR 6. NR 7. NR 8. NR 9. **Age:** 20-56 yrs; mean 33.75±11.02 yrs* | 1. Negtive association between δ pwr during early NREM sleep & age. 2. Greater age-related decline in δ pwr seen in M |
| Kluge M. et al. (2010); *PNEC.* | 1. Germany; Bavaria; Munich; Max Planck Institute of Psychiatry 2. Study ghrelin’s impact on sleep, the secretion of growth hormone (GH) and cortisol in elderly people 3. Research concerns sex diff and age 4. Given by participant / NR 5. NA | 1. NR 2. Healthy **\|** NR 3. No caff, alc, naps **\|** influence vigilance 4. NR | 1. NR 2. a. Any sleep disturbance/d/o **\|** NR 3. Any meds/HRT **\|** NR 4. a. Sleep duration of ≤6h/≥9h **\|** NR 5. Transmeridian flight 3 mos pre-study **\|** NR 6. Depressive symptoms **\|** sleep disturbance | 1. NR 2. NR 3. NR 4. 10M, 10F† (50%)* 5. NR 6. NR 7. NR 8. NR 9. **Age:** range 60-70 yrs; mean 64±2.2 yrs (M), 63±2.9 yrs (F)* | 1. Ghrelin affects sleep in elderly M but not F resembling findings in young subjects 2. F sex hormones has an affect on sleep patterns |
| Latta, F. et al. (2005); *Sleep.* | 1. USA; Illinois; Chicago; University of Chicago; General Clinical Research Center 2. Examine sex effects on sleep stages and EEG spectral power in older adults 3. Research concerns sex diff and age 4. Given by participant / NR 5. 1 F recording lost due to technical error | 1. NR 2. a. Nonobese pcps **\|** NR 3. Normal lab tests **\|** NR 4. No hx of psy/sleep/neuro/endocrine d/o **\|** NR 5. No meds/HRT **\|** NR 6. a. Non-smoking **\|** NR 7. <5 on GDS, >25 on FMSE **\|** NR 8. F 1+ y past menopause w no hot flash **\|** NR | 1. Shift workers **\|** NR 2. Diabetes/glucose intolerance **\|** NR 3. a. 2+ caff drinks per d **\|** NR 4. Transmeridian travel 4 weeks pre-study **\|** NR 5. NR | 1. Illinois, Chicago ‡ 2. NR 3. NR 4. 10M, 10F† (50%)* 5. NR 6. NR 7. NR 8. NR 9. **Age:** mean 59±2 yrs (M), 63±2 yrs (F) | 1. Sex diff in δ activity were more pronounced in REM than in NREM 2. When normalized for REM activity, the sex diff in δ activity in NREM reversed, showing ↓ activity in F 3. Sleep-stage distribution was similar in both sex groups |
| Luo X. et al. (2024);  *Front. Psychol*. | 1. NR; Sleep laboratory 2. To investigate the relationship between age, gender, and EEG functional connectivity 3. Research concerns sex diff and age 4. Given by participant / NR 5. Incomplete data | 1. NR 2. No sleep-related meds **\|** NR 3. NR 4. NR | 1. NR 2. NR 3. NR 4. Incomplete data **\|** NR | 1. NR 2. NR 3. NR 4. 56M, 79F (41%)* 5. NR 6. NR 7. NR 8. NR 9. **Age:** range 25-101 yrs; mean 57.89±21.34 yrs (M), 54.39±21.65 yrs (F)* | 1. Diff in functional connectivity was seen btw diff age groups 2. F had stronger functional connectivity of high-band |
| Ma, J. et al. (2011); *J Clin. Sleep Med.* | 1. USA; Sleep laboratory 2. Study gaboxadol’s effect on NREM sleep EEG patterns in transient insomnia using power spectral analysis 3. Research concerns sex diff 4. Given by participant / NR 5. NA | 1. 18-64 y **\|** NR 2. NR 3. NR 4. NR | 1. NR 2. a. Sleep/psy d/o **\|** NR 3. >5 on PSQI/>12 on ESS **\|** NR 4. Sleep apnea/PLMD **\|** NR 5. Sleep duration 6.5-9h, bedtime btw 21:00-24:00 ≥4 times a wk, latency <30 min **\|** NR 6. a. MSLT <10 min **\|** NR 7. Excessively sleepy pcps **\|** NR 8. NR | 1. NR 2. NR 3. NR 4. 314M, 508F (38%)* 5. NR 6. NR 7. NR 8. NR 9. **Age:** range 18-64 yrs; mean 28.78±8.21 yrs (M), 31.76±10.70 yrs (F) | 1. The effect was sex-dependent, with a ↑ effect observed in F than M 2. There were no differences in MSLT, sleep efficiency, (BMI), or age btw the 3 treatment groups, or btw M and F within them |
| Markovic A. et al (2020); *Sci Rep.* | 1. Switzerland; Bern; Subject’s homes 2. Examine sex diff in sleep EEG power and coherence across freq bands in NREM and REM in adolescents 3. Research concerns sex diff and age 4. Given by parent and assented by participant / NR 5. NA | 1. 9-14 yrs \| NR 2. NR 3. NR 4. Born after 30th week of pregnancy \| NR | 1. NR 2. NR 3. NR 4. Poor quality EEG data \| unable to analyze data | 1. NR 2. NR 3. NR 4. 30M, 31F (49%)* 5. NR 6. NR 7. NR 8. NR 9. a. **Age**: range 9-14 yrs; mean 12.83±0.75 yrs (M), 12.12±1.67 yrs (F)* 10. **Tanner stage**: mean 2±1 (M), 3±1 (F)* | 1. M showed a ↓ and F showed an ↑ of SPDL freq with age 2. Observed a significant interaction btw age and gende for all frequencies, with an exception of θ and α, both sleep states with M experiencing ↑ in coherence at older age as compared to F |
| Mongrain, V. et al. (2005); *Sleep.* | 1. Canada; Quebec; Montreal; Sacré-Cœur Hospital of Montréal; Chronobiology laboratory 2. Explore relationship of chronotype on sleep stages and quantitative sleep EEG btw sexes 3. Research concerns sex diff 4. Given by participant / NR 5. NA | 1. No shift work **\|** NR 2. Healthy **\|** NR 3. a. Nonsmoker **\|** NR 4. >85% sleep efficiency, latency <30 min, AHI+PLMI <5 per h, MSLT >7 min **\|** NR 5. No drugs **\|** NR 6. Transmeridian travel 3 mos pre-study **\|** NR 7. NR | 1. NR 2. NR 3. a. Bedtime btw 7-9h **\|** NR 4. 3+ alc drink/2+ caff drink per day **\|** NR 5. NR | 1. NR 2. French questionnaire used 3. 18 pcps were students without a summer job, 2 were between jobs, 1 person only worked in the afternoon, and 3 worked at home with their own schedule 4. 12M, 12F† (50%)* 5. NR 6. NR 7. NR 8. NR 9. **Age**: range 19-34 yrs; mean 24.7±1.5 yrs (Morning type), 23.4±0.7 yrs (Evening type) | 1. Morningness-eveningness seems to affect sleep in a sex-specific manner, with M being > affected by their chronotypes 2. Prominent sex diff found in high σ range |
| Mourtazaev M. S. et al. (1995); *Sleep.* | 1. Netherlands; South Holland (Zuid-Holland); Leiden; Participant’s homes 2. Establish how age and sex affect NREM EEG; whether neuronal SW-generating mechanism is involved 3. Research concerns sex diff and age 4. NR / NR 5. NA | 1. NR 2. a. Healthy \| NR 3. No somatic/neuro/psy d/o \| NR 4. No use sleep-related meds\| NR 5. No sleep complaints \| NR 6. NR | 1. NR 2. NR 3. NR 4. NR | 1. Leiden, Netherlands ‡ 2. NR 3. NR 4. 27M, 32F (46%)* 5. NR 6. NR 7. NR 8. NR 9. **Age**: range 26–35 yrs (Young), 51–60 yrs (Middle), 66–75, 85–101 yrs (Late adulthood)* | 1. SWS decreases with age. The age effect from 30 to 55 years is substantial: it is about equal to interindividual differences 2. In all age groups, F had sign > total SWP; in all age groups except 85–101, F had sign > max SWP (calculated by SR authors using mean and SD data) |
| Pun M. et al. (2023);  *J Sleep Res*. | 1. Canada, Alberta, Calgary, University of Calgary, Cumming School of Medicine 2. Examine relationship btw sleep spindle characteristics and cognitive function in older adults 3. Research concerns sex 4. Given by participant/NR 5. NA | 1. NR 2. a. BMI < 35 kg m^-2^ **\|** NR 3. No cog decline (MoCA ≥24) **\|** NR 4. a. Non-smoker for 1+ yrs **\|** NR 5. Sedentary (<30 min of exercise 4d/wk) **\|** NR 6. NR | 1. NR 2. NR 3. NR 4. NR | 1. NR 2. NR 3. NR 4. 7M, 14F (67%)* 5. NR 6. 17.8±3.2 yrs 7. NR 8. NR 9. **Age:** range 51-80 yrs; mean 66.86±8.6 yrs (M), 65.9±5.0 yrs (F) | 1. Middle aged pcps have ↓spindle density is associated with poor cognitive performance. 2. F had more NREM stage 2 sleep which may be attributed to hormonal differences |
| Ringli M. et al. (2013); *Int J Psychophysiol.* | 1. Switzerland; Zurich; University Children’s Hospital; Child Development Center 2. Examine whether sexually dimorphic features are reflected in topography of sleep SWA 3. Research concerns sex diff 4. Given by participant or parents for underage subjects / NR 5. NA | 1. Right-handed **\|** NR 2. No psychopathology/chronic/primary sleep d/o **\|** NR 3. a. Non-smoker **\|** NR 4. No sleep complaints **\|** NR 5. NR | 1. NR 2. On psychoactive agent/meds **\|** NR 3. a. >4 cups of coffee/same amount of caff per d, ≤1 glass of alc **\|** NR 4. NR | 1. NR 2. NR 3. NR 4. 11M, 11F (50%)* 5. NR 6. NR 7. NR 8. NR 9. a. **Age:** range 8.7-19.4 yrs (M), 9.1-19 yrs (F); mean 13.4±3.9 yrs (M), 13.4±3.9 yrs (F)* 10. **Tanner stage:** mean 6.3±3.6 (M), 7.6±3.9 (F) | 1. Age unrelated 2. Cortical areas governing functions in which one sex outperforms other exhibit ↑ sleep SWA and may indicate maturation of sex-specific brain function and higher cortical plasticity during development |
| Rosinvil T. et al. (2021);  *SleepJ.* | 1. Canada, Quebec, Montreal, Hôpital du Sacré-Coeur de Montréal 2. Investigate a data-driven approach to identifying age and sex differences in SW 3. Research concerns sex diff and age 4. Given by participant 5. NA | 1. NR 2. a. No drugs **\|** NR 3. No symptoms of depression/anxiety **\|** NR 4. AI <10 **\|** NR 5. PLMI <10 **\|** NR 6. NR 7. a. Premenopausal women had regular cycle in last yr **\|** NR 8. Menopausal women had no cycle for 1+ yrs & no vasomotor complaints **\|** NR | 1. Night worker **\|** NR 2. On sleep-affecting meds **\|** NR 3. a. Smoker **\|** NR 4. Self-reported sleep complaints **\|** NR 5. Unusual sleep duration (<7h/>9h) **\|** NR 6. Transmeridian travel 3 mos pre-study **\|** NR | 1. NR 2. NR 3. NR 4. 284 (132M/152F)   C1-younger: 97 (45M/52F)  C1-older: 110 (49M/61F)  C2-younger: 38 (19M/19F)  C2-older: 39 (19M/20F)*   1. NR 2. NR 3. NR 4. NR 5. **Age:** range 20-71 yrs; mean   23.8±2.8 yrs (C1-younger)  57.5±5.1 yrs (C1-older)  22.7±2.4 yrs (C2-younger)  59.6±5.4 yrs (C2-older )* | 1. Older adults show overall less SW but more likely to produce low amp SW. 2. Sex may impact amplitude of SW through sleep regulatory mechanisms unrelated to SW generation |
| Ujma P. P. et al. (2019); *Neurobiol Aging.* | 1. Hungary/Germany; Central Hungary/Bavaria; Budapest/Munich; Max Planck Institute of Psychiatry/Institute of Behavioral Sciences of Semmelweis University 2. Investigate age and sex effects on sleep EEG functional connectivity in adults 3. Research concerns sex diff and age 4. Given by participant / NR 5. NA | 1. NR 2. a. No drugs except CC **\|** NR 3. No neuro/psy d/o **\|** NR 4. <2 cups of caff before noon **\|** NR 5. NR | 1. NR 2. NR 3. NR 4. Poor EEG data **\|** unable to obtain data needed for analysis | 1. NR 2. NR 3. NR 4. 94M, 78F (55%)* 5. NR 6. NR 7. NR 8. NR 9. **Age:** range 17-69 yrs; mean 29.74±10.71 yrs* | 1. General intelligence was not significantly associated with connectivity in either sex 2. NREM EEG connectivity in σ freq range increases ↓ with aging 3. Connectivity was substantially greater in F than in M in high σ freq range |
| Ujma P.P. et al. (2022);  *Sci Rep*. | 1. Gemany/Hungary, Munich/Budapest, Max Institute of Psychiatry/Psychophysiology and Chronobiology Research Group of Semmelweis University, Sleep Laboratory 2. Study EEG envelope spectrum through comparing EEG of epileptic patient and healthy pcps 3. Research concerns sex diff and age 4. Given by participant / NR 5. NA | 1. NR 2. a. Healthy **\|** NR 3. Hx of neuro/psy d/o **\|** NR 4. No drugs except CC **\|** NR 5. a. >2 cups of coffee before noon **\|** NR 6. No alc **\|** NR 7. NR | 1. NR 2. NR 3. NR 4. NR | 1. NR 2. NR 3. NR 4. 95M, 81F (54%)* 5. NR 6. NR 7. NR 8. NR 9. **Age:** range 17-69 yrs; mean 29.8±10.66 yrs* | 1. Low freq NREM β rhythm was lower in M. NREM envelope spectrum was a highly reliable individual marker strongly associated with ageing & partially sexually dimorphic |
| Ventura S. et al. (2022);  *SLEEPJ*. | 1. Ireland, Cork, Cork University Maternity Hospital; Sleep laboratory 2. Investigate influences of sex on macro sleep structure and sleep spindles for infacts of 4-5 mos 3. Research concerns sex diff 4. Obtained / NR 5. One did not fall asleep, 1 had abonormal EEG, did not have Griffith's-III neurodevelopmental assessment, GDQ score was <=85 , failed macrostructure, had REM before reaching N2 | 1. NR 2. Healthy **\|** NR 3. NR 4. a. Born after 37 wks of GA **\|** NR 5. Singleton **\|** NR | 1. NR 2. NR 3. NR 4. NR | 1. NR 2. Mother's ethnicity:   White Irish: 90.1%; Non-Irish White: 6.6%; Asian: 2.2%; Latin-American: 1.1%  Father’s ethnicity:  White Irish: 90.1%; Non-Irish White: 6.6%; Asian: 2.2%; Arabic: 1.1%   1. NR 2. 54M, 37F (59%) *† 3. NR 4. NR 5. NR 6. NR 7. **Age**: range 4-5 mos; mean 39.8 wks±1.2 wks*   **Postnatal age of mother**: 19.5±1.3 wks*  **Postmenstrual age of mother:** 59.3±1.8 wks* | 1. Fast spindle density higher in F 2. No sex diff in sleep macrostructure parameters |
| Yoon J. et al. (2021); *J Sleep Res.* | 1. Korea; Gyeonggi; Ansan; Subject’s homes 2. Identify role of age and sex in sleep structure with EEG spectral power analyses of middle-aged/older adults 3. Research concerns sex diff and age 4. NR / NR 5. NA | 1. NR 2. NR 3. NR 4. Underwent PSG protocol in the 6^th^ biennial exam btw 2011-2012 **\|** NR | 1. NR 2. Major neuro/psych d/o **\|** NR 3. Substance abuse **\|** NR 4. Poor quality EEG **\|** unable to perform spectral analysis | 1. Gyeonggi, Ansan 2. Korean 3. NR 4. 330M, 314F (51%)* 5. NR 6. NR 7. NR 8. NR 9. **Age:** range 45-69 yrs; mean 57.7±6.5 yrs (M), 57.9±6.9 yrs (F) | 1. ↑age is negatively associated with sleep quality and quantity – diff are more pronounced in M than F 2. Age does not affect sleep in a uniform matter 3. Discussion on N3 being proportionally less in Koreans than Caucasians |
| Yuksel D. et al. (2020);  *Sleep Health.* | 1. USA, California, Menlo Park, SRI International; Sleep laboratory 2. Study sex & age diff in stress related sleep disturbances 3. Research concerns sex diff 4. Given by participant / NR 5. NA | 1. NR 2. a. Free of major mental & phy condition **\|** NR 3. No insomnia/other sleep d/o **\|** NR 4. Keep sleep-wake cycle for 5d **\|** NR 5. NR | 1. NR 2. NR 3. NR 4. NR | 1. California, Davis‡ 2. Caucasian: 45 (M), 37 (F) 3. NR 4. 57M, 49F (54%)* 5. NR 6. NR 7. NR 8. NR 9. **Age:** range 12.1-19.9; mean 15±1.9 yrs (M), 15.3±2 yrs (F)   **Pubertal Developmental Scale**: 2.7±0.7 (M), 3.3±0.7(F) | 1. ↑nocturnal HR during REM and NREM in F 2. More ANS upregulation in older F |
| Zhang Y. Z. et al. (2021);  *J Neurosci*. | 1. USA, California, Davis, University of California, Davis 2. Compile a dataset of longitudinal measurements of spindle microstructure in adolescence 3. Research concerns sex diff and age 4. Given by participant / NR 5. NR | 1. NR 2. NR 3. NR 4. NR | 1. NR 2. NR 3. NR 4. NR | 1. NR 2. NR 3. NR 4. 51M, 47F (52%)   C6: 17M, 11F (61%)  C9: 15M, 17F (47%)  C12: 19M, 19F (50%)*   1. NR 2. NR 3. NR 4. NR 5. **Age:** range 5.96-18.4 yrs* | 1. Central spindle amp ↓from 12 to 16 yrs of age, aligning with the timing of synaptic pruning 2. Linear projection of age-related change in spindle freq |

*Parameters considered in analysis of sex diff; † Study uses the terms men and women (gender) but is referring to sex; ‡ Study indicated the recruitment took place in the local population.

*Abbreviations:* **AI**, Apnea-hypopnea Index; **alc**, alcohol; **ANOVA**, analysis of variance; **BDS**, Beck Depression Scale; **btw**, between; **BMI**, Body Mass Index; **C,** Cohort; **caff**, caffeine; **CC**, contraceptive; **cog,** cognitive; **CNS**, Central Nervous System; **CS**, Cross-sectional; **d**, days; **diff**, differences; **DMS-IV,** Diagnostic and Statistical Manual of Mental Disorders, Fourth Edition; **d/o**, disorder; **EEG**, electroencephalogram; **ESS**, Epworth Sleepiness Scale **F**, female; **freq**, frequency; **FMSE**, Folstein Mini-Mental Status Examination; **FS**, fast spindle; **GA**, gestational age; **GDS**, Geriatric Depression Scale Short Form; **Hx**, History; **HRT**, Hormone Replacement Therapy; **IQ**, Intelligence Quotient; **L**, longitudinal; **M**, male; **Meds**, medicine; **Min**, minutes; **Mos**, months; **MSLT**, Multiple Sleep Latency Test; **neuro**, neurological; **NR**, not reported; **NREM**, non-REM; **pcp**, Participants; **PDS**, Pubertal Developmental Scale; **phys**, Physical; **PLMI**, Periodic Limb Movements Index; **PLMAI**, Periodic Limb Movements Arousal Index; **PLMD**, Periodic Limb Movement Disorder; **PMA**, postmenstrual age; **PNA**, postnatal age; **PSG**, Polysomnographic recording; **PSQI**, Pittsburgh Sleep Questionnaire Inventory; **psy**, psychiatric; **REM**, rapid eye movement; **sig**, significant; **SPLD**, spindle; **SWA**, Slow Wave Activity; **w**, with; **wk**, week; **wt**, weight; **w/o**, without; **w/i**, within; **yrs**, years. Symbols: ↓, decreased; ↑, increased, δ, delta; σ, sigma
