# Supplementary material for: Sex differences in electrical activity of the brain during sleep: a systematic review of electroencephalographic findings across the human lifespan
Source: Biomed Eng Online. 2025 Mar 12;24:33. doi: 10.1186/s12938-025-01354-z (PMC11899717; doi:10.1186/s12938-025-01354-z)
Supplement: Supplementary file 3 — Supplementary material 3. [file 12938_2025_1354_MOESM3_ESM.docx]

**Supplementary Material S3.** Statistical approaches for the consideration of Plus variables in the study of sex differences.

| **Study** | **Investigated Parameter** | **Statistical Analysis** | **Key Findings** |
| --- | --- | --- | --- |
| **Armitage (2000)** | Age effect on delta amplitude | Regression (age effect by group & sex) | Significant agre-related decrease in normal control women (R² = 0.76, p = 0.01) |
| **Armitage (2000)** | Age effect on delta power | Regression (age effect by group & sex) | Age effect statistically significant for normal control women (R² = 0.62, p = 0.04) |
| **Campbell (2005)** | Delta power density & Tanner stage correlation | Correlation coefficient analysis | Weak negative correlation in girls at first recording (r = -0.38 for C9, r = -0.46 for C12) |
| **Campbell (2012)** | Age of steepest delta decline | Gompertz equation fit; Nonlinear mixed effect analysis; Structural equation modeling | Sex + Tanner explained 67% of variation (p < 0.0001) |
| **Carrier (2001)** | EEG spectral density across NREM | Mixed model analysis (effects of age, gender, NREM period) | Significant main effect of gender (p = 0.001) and interaction gender x NREM period (p = 0.027) |
| **Dijk (1989)** | Decay of EEG power density | Nonlinear regression | Absolute power density: female Po and P00 significantly greater (p < 0.01); relative power density: males greater Po (p < 0.05) and females greater P00 (p < 0.01) |
| **Ventura (2022)** | Sleep spindle spectral power | Univariate & multivariate analyses | Higher in females (p = 0.022 [univariate], p = 0.032 [multivariate]) |
| **Yoon (2021)** | Absolute spectral power | Multiple regression | Sex differences not significant in regression |
| **Zhang (2021)** | Spindle density peak | Mixed-effect models | Sex differences not significant in peak or age of peak (p = 0.40 [density], p = 0.84 [age of peak]) |
