# Supplementary material for: Sex differences in electrical activity of the brain during sleep: a systematic review of electroencephalographic findings across the human lifespan
Source: Biomed Eng Online. 2025 Mar 12;24:33. doi: 10.1186/s12938-025-01354-z (PMC11899717; doi:10.1186/s12938-025-01354-z)
Supplement: Supplementary file 4 — Supplementary material 4. [file 12938_2025_1354_MOESM4_ESM.docx]

**Supplementary Material S4**

**Supplementary Material S4a**. QUIPS Six Potential Sources of Bias and assessment criteria

| **QUIPS Six Potential Sources of Bias** | | **Assessment Criteria** | |
| --- | --- | --- | --- |
| 1. | Study Design | 1. | Was the research question or objective in this paper clearly stated? |
|  |  | 10. | Was the exposure(s) assessed more than once over time? |
|  |  | 12. | Were the outcome assessors blinded to the exposure status of participants? |
| 2. | Study Participation | 2. | Was the study population clearly specified and defined?  *Authors reported at least 2/3 of the following: age, substance intake, study setting.* |
|  |  | 3. | Was the participation rate of eligible persons at least 50%? |
|  |  | 4. | Were all the subjects selected or recruited from the same or similar populations (including the same time period)? Were inclusion and exclusion criteria for being in the study prespecified and applied uniformly to all participants? |
| 3. | Study Attrition | 13. | Was loss to follow-up after baseline 20% or less? |
| 4. | Associated Factors | 9. | Were the exposure measures (independent variables) clearly defined, valid, reliable, and implemented consistently across all study participants? |
| 5. | Outcome Measures/ Confounding Account | 11. | Were the outcome measures (dependent variables) clearly defined, valid, reliable, and implemented consistently across all study participants? |
|  |  | 14. | Were key potential confounding variables measured and adjusted statistically for their impact on the relationship between exposure(s) and outcome(s)? *Authors considered (at least) key potential confounding variable age.* |
| 6. | Analysis | 5. | Was a sample size justification, power description, or variance and effect estimates provided? |
|  |  | 6. | For the analyses in this paper, were the exposure(s) of interest measured prior to the outcome(s) being measured? |
|  |  | 7. | Was the timeframe sufficient so that one could reasonably expect to see an association between exposure and outcome if it existed? |
|  |  | 8. | For exposures that can vary in amount or level, did the study examine different levels of the exposure as related to the outcome (e.g., categories of exposure, or exposure measured as continuous variable)? |

**Supplementary Material S4b.** Summary of scoring

| **1. Crude Score** | Each Assessment Criteria was rated as yes (Y), no (N), cannot determine (CD), not reported (NR), or not applicable (NA). The crude score was calculated by dividing the number of 'yes' (Y) responses by the total number of applicable criteria. Criteria rated as not applicable (NA) were excluded from the total. |
| --- | --- |
| **2. Source of Bias Rating** | Each Source of Bias was rated as “+”, “-”, or not applicable (NA):   - **“+”** when most Assessment Criteria were rated as yes (Y) in that Source of Bias category. - **“-”** when one or more Assessment Criteria were rated as no (N) in that Source of Bias category. - **Not applicable (NA)** when all Assessment Criteria in that Source of Bias category were rated as NA. |
| **3. Overall Rating** | Finally, each study quality was summarized qualitatively into three groups:   - **Excellent (“++”)** when all or most of the Sources of Bias were rated as “+”. Studies that had more than one Assessment Criteria rated as not reported (NR) or cannot determine (CD) cannot be as rated “++”. - **Good (“+”)** when more than 50% of the Sources of Bias were rated as “+”. - **Fair (“-”)** when 50% or more of the Sources of Bias were rated as “-”. |

**Supplementary Material S4c**. Quality assessment of studies from original searches, using the QUIPS Six Potential Sources of Bias

| **Study** | **Assessment Criteria** | | | | | | | | | | | | | | **Crude Score** | **Source of Bias Rating** | | | | | | | | | **Overall Rating** |
| --- | --- | --- | --- | --- | --- | --- | --- | --- | --- | --- | --- | --- | --- | --- | --- | --- | --- | --- | --- | --- | --- | --- | --- | --- | --- |
|  | **1** | **2** | **3** | **4** | **5** | **6** | **7** | **8** | **9** | **10** | **11** | **12** | **13** | **14** |  | **1** | **2** | **3** | **4** | | **5** | | **6** | |  |
| Armitage et al 1995 | Y | Y | NR | CD (CD/Y) | N | Y | NA | NA | Y | NA | Y | NA | NA | N | 5/9 | + | - | NA | | + | | - | | - | **-**  **Fair** |
| Armitage et al 2000 | Y | Y | NR | CD (CD/Y) | Y | Y | Y | NA | Y | NA | Y | NA | Y | Y | 9/11 | + | - | + | | + | | + | | + | **+**  **Good** |
| Baker et al 2012 | Y | Y | NR | Y (Y/Y) | N | Y | Y | NA | Y | NA | Y | NA | Y | Y | 9/11 | + | + | + | | + | | + | | + | **++**  **Excellent** |
| Campbell et al 2005 | Y | Y | NR | CD (CD/CD) | Y | Y | Y | NA | Y | NA | Y | NA | Y | Y | 9/11 | + | - | + | | + | | + | | + | **+**  **Good** |
| Campbell et al 2012 | Y | Y | NR | CD (Y/CD) | Y | Y | Y | NA | Y | NA | Y | NA | Y | Y | 9/11 | + | - | + | | + | | + | | + | **+**  **Good** |
| Carrier et al 2001 | Y | Y | NR | CD (CD/Y) | Y | Y | Y | NA | Y | NA | Y | NA | Y | Y | 9/11 | + | - | + | | + | | + | | + | **+**  **Good** |
| Carrier et al 2011 | Y | Y | NR | CD (CD/Y) | Y | Y | NA | NA | Y | NA | Y | NA | NA | Y | 7/9 | + | - | NA | | + | | + | | + | **+**  **Good** |
| Dijk et al 1989 | Y | Y | NR | CD (CD/Y) | Y | Y | NA | NA | Y | NA | Y | NA | NA | N | 6/9 | + | - | NA | | + | | - | | + | **+**  **Good** |
| Feinberg et al 2006 | Y | Y | NR | Y (Y/Y) | Y | Y | Y | NA | Y | NA | Y | NA | NR | Y | 9/11 | + | + | - | | + | | + | | + | **+**  **Good** |
| Fukuda et al 1999 | Y | Y | NR | CD (CD/Y) | N | Y | Y | NA | Y | NA | Y | NA | Y | N | 8/11 | + | - | + | | + | | - | | - | **-**  **Fair** |
| Kluge et al 2010 | Y | Y | NR | CD (CD/Y) | Y | Y | Y | NA | Y | NA | Y | NA | Y | N | 8/11 | + | - | + | | + | | - | | + | **+**  **Good** |
| Latta et al 2005 | Y | Y | NR | Y (Y/Y) | Y | Y | Y | NA | Y | NA | Y | NA | Y | N | 9/11 | + | + | + | | + | | - | | + | **++**  **Excellent** |
| Ma et al 2011 | Y | N | NR | N (Y/N) | N | Y | NA | NA | Y | NA | Y | NA | NA | N | 5/9 | + | - | NA | | + | | - | | - | **-**  **Fair** |
| Markovic et al 2020 | Y | Y | NR | CD (CD/Y) | N | Y | NA | NA | Y | NA | Y | NA | NA | Y | 6/9 | + | - | NA | | + | | + | | - | **+**  **Good** |
| Mongrain et al 2005 | Y | Y | NR | CD (CD/Y) | Y | Y | Y | NA | Y | NA | Y | NA | Y | N | 8/11 | + | - | + | | + | | - | | + | **+**  **Good** |
| Ringli et al 2013 | Y | Y | NR | CD (CD/Y) | N | Y | NA | NA | Y | NA | Y | NA | NA | Y | 6/9 | + | - | NA | | + | | + | | - | **+**  **Good** |
| Ujma et al 2019 | Y | Y | NR | N (N/Y) | Y | Y | NA | NA | Y | NA | Y | NA | NA | Y | 7/9 | + | - | NA | | + | | + | | + | **+**  **Good** |
| Yoon et al 2021 | Y | Y | Y | Y (Y/Y) | Y | Y | NA | NA | Y | NA | Y | NA | NA | Y | 9/9 | + | + | NA | | + | | + | | + | **++**  **Excellent** |

*Abbreviations:* **CD**, cannot determine; **N**, no; **NA**, not applicable; **NR**, not reported; **Y**, yes

**Supplementary Material S4d**. Quality assessment of studies from repeated searches, using the QUIPS Six Potential Sources of Bias

| **Study** | **Assessment Criteria** | | | | | | | | | | | | | | **Crude Score** | **Source of Bias Rating** | | | | | | | | | **Overall Rating** |
| --- | --- | --- | --- | --- | --- | --- | --- | --- | --- | --- | --- | --- | --- | --- | --- | --- | --- | --- | --- | --- | --- | --- | --- | --- | --- |
|  | **1** | **2** | **3** | **4** | **5** | **6** | **7** | **8** | **9** | **10** | **11** | **12** | **13** | **14** |  | **1** | **2** | **3** | **4** | | **5** | | **6** | |  |
| Dorokhov et al 2024 | Y | Y | NR | Y (Y/Y) | Y | Y | CD* | NA | Y | NA | Y | NA | NR | N | 7/11 | + | + | - | | + | | - | | + | **+**  **Good** |
| Hejazi et al 2024 | Y | Y | NR | CD (CD/Y) | Y | Y | Y | NA | Y | NA | Y | NA | NR | Y | 9/11 | + | - | - | | + | | + | | + | **+**  **Good** |
| Luo et al 2024 | Y | N | CD** | N (N/CD) | Y | Y | Y | NA | Y | NA | Y | NA | NA | N | 6/10 | + | - | NA | | + | | - | | + | **+**  **Good** |
| Mourtazaevet al 1995 | Y | Y | NR | Y (Y/Y) | Y | Y | Y | NA | Y | NA | Y | NA | N | Y | 9/11 | + | + | - | | + | | + | | + | **+**  **Good** |
| Pun et al 2023 | Y | Y | NR | N (N/N) | Y | Y | Y | NA | Y | NA | Y | NA | NA | N | 7/11 | NA | - | NA | | + | | - | | + | **-**  **Fair** |
| Rosinvil et al 2021 | Y | Y | NR | N  (N/CD) | Y | Y | Y | NA | Y | NA | Y | NA | NA | Y | 8/10 | + | - | NA | | + | | + | | + | **+**  **Good** |
| Ujma et al 2022 | Y | Y | NR | CD (CD/Y) | N | Y | Y | NA | Y | NA | Y | NA | NR | Y | 7/11 | + | - | - | | + | | + | | + | **+**  **Good** |
| Ventura et al 2022 | Y | Y | NR | Y (Y/Y) | N | Y | Y*** | NA | Y | NA | Y | NA | NR | Y | 8/11 | + | + | - | | + | | + | | + | **+**  **Good** |
| Yuksel et al 2021 | Y | Y | NR | CD (CD/Y) | Y | Y | Y | NA | Y | NA | Y | NA | NA | N | 7/10 | + | - | NA | | + | | - | | + | **+**  **Good** |
| Zhang et al 2021 | Y | Y | NR | CD (CD/Y) | Y | Y | Y | NA | Y | NA | Y | NA | Y | Y | 9/11 | + | - | + | | + | | + | | + | **+**  **Good** |

*Abbreviations:* **CD**, cannot determine; **N**, no; **NA**, not applicable; **NR**, not reported; **Y**, yes

*CD since this was a nap study
**CD since the data was coming from a database of multiple studies
***Y since these were babies and their sleep patterns were not formed for day and night sleep
