# Supplementary material for: Sex differences in electrical activity of the brain during sleep: a systematic review of electroencephalographic findings across the human lifespan
Source: Biomed Eng Online. 2025 Mar 12;24:33. doi: 10.1186/s12938-025-01354-z (PMC11899717; doi:10.1186/s12938-025-01354-z)
Supplement: Supplementary file 5 — Supplementary material 5. [file 12938_2025_1354_MOESM5_ESM.pdf]

Supplementary Material S5

PROSPERO
International prospective register of systematic reviews

Sex Differences in Sleep Electroencephalography Markers Among Healthy Human Subjects

Tatyana Mollayeva, Chi Kiu Lee, Jessica Babineau

To enable PROSPERO to focus on COVID-19 submissions, this registration record has undergone basic automated checks for eligibility and is published exactly as submitted. PROSPERO has never provided peer review, and usual checking by the PROSPERO team does not endorse content. Therefore, automatically published records should be treated as any other PROSPERO registration. Further detail is provided **here**.

Citation

Tatyana Mollayeva, Chi Kiu Lee, Jessica Babineau. Sex Differences in Sleep Electroencephalography Markers Among Healthy Human Subjects. PROSPERO 2022 CRD42022327644 Available from: https://www.crd.york.ac.uk/prospero/display_record.php?ID=CRD42022327644

Review question

To investigate sex difference in sleep electrophysiological markers (i.e., EEG waves, local fields potential, etc) in healthy human subjects.

Searches

Search terms used: evoked response potential, spectral analysis, power spectral density, sleep electroencephalogram; EEG, delta activity, alpha activity, beta activity, theta activity, gamma activity, sigma spectral power, sleep stages, slow wave, nonrapid eye movement sleep, rapid eye movement, polysomnography, plasticity, sleep spindle, synchronization

Types of study to be included

Observational studies (prospective cohort; cross-sectional, case control, case series)

Condition or domain being studied

Sex difference in electrical activity in sleep

Participants/population

Healthy humans

Intervention(s), exposure(s)

None

Comparator(s)/control

Male versus female persons

Context

With the increased diagnostic and investigative utility of electroencephalography (EEG) in sleep medicine, and attention to scientific inquiry concerning sex differences in brain structure and function, interest in the scientific study and analysis of electrical signals generated by male and female brains has been activated. We aim to summarise scientific evidence regarding sex differences in sleep EEG activity in healthy humans. We will include studies that sufficient descriptive data of any given measure of sleep EEG markers, separately for female and male healthy participants of all ages. Studies reporting samples with any disorder or medical condition will be excluded.

Main outcome(s)

Sleep electroencephalography markers

Measures of effect

Any measure of effect as reported by researchers.

Additional outcome(s)

Not applicable

Measures of effect

Not applicable

Data extraction (selection and coding)

The authors' name, the year of publication, country of study origin, the sample size studied, the relative number of male and female persons, and the age of male and female persons will be retrieved from all included studies, along with information on all studied covariates. We will consist of all sleep EEG markers reported, including frequency, power, synchronization, etc. Descriptive data on the reported sleep EEG markers will be extracted separately for male and female persons.

Risk of bias (quality) assessment

Two reviewers independently will assess study quality using the National Institutes of Health study quality assessment tools. The validity of each study will be appraised in two steps. The first step will assess items related to potential sources of bias according to the most critical criteria for external and internal design validity within the cohort, cross-sectional, case-control, or case series. The second step will judge the presence of potential biases as “yes,” “not reported,” or “cannot determine.” The two reviewers will initially meet for a calibration review. They will independently review one study of each type and discuss each item on the list to clarify its meaning and interpretation. Following this, the methodological quality of each study will be rated across a set of items, independently by the same two reviewers. In cases of disagreement between the two reviewers, a team discussion will take place, with the goal of consensus being reached in each case. We will use the Scottish Intercollegiate Guidelines Network methodology to summarize the evidence as follows: ++ (high) when all quality criteria are fulfilled, permitting one “cannot determine;” + (moderate) when most criteria are fulfilled, and − (low) when few criteria are fulfilled.

Strategy for data synthesis

If studies’ heterogeneity values permit, we will conduct a meta-analysis, which will be based on means, standard deviations, and the sample size. Transformations will be applied if data are presented in median and/or range. If studies reporting sleep EEG markers are stratified by the recording time (e.g., first night-consecutive night, etc.), age (e.g., children versus youth, etc.), or other variables (e.g., intelligence level, etc.), data will be extracted for all subgroups, as long as male and female persons were compared under the same condition. If heterogeneity across PICOs will not allow meta-analysis, we will summarize the evidence and

present an overview of the findings across sleep EEG markers according to developmental stage. We will also organize and present the results according to the approach taken to investigate sex, using forest plots to depict sex differences reported as odds with confidence intervals. Studies that offered β coefficients and partial R^2 will be depicted in pie charts, showing the contributions of different variables, including sex, to the studied outcome. Finally, studies that stratified results by sex will be reported on separately.

Analysis of subgroups or subsets

We will organize studied sleep EEG markers into the following categories: Alpha, 7.5 to 13 Hz; Beta, >14 Hz; Theta, 3.5 to 7.5 Hz; Delta, <3 Hz and Other. We will group the results, if data permit, according to developmental stage (e.g., children, adolescents, adults, and elderly). Finally, to capture and interpret other characteristics that intersect with and contribute to sex effects in the study results, we will monitor the inclusion of all potential covariates, including education, socioeconomic status, and other studied variables related to the outcomes of interest.

Contact details for further information

Tatyana Mollayeva
tatyana.mollayeva@utoronto.ca

Organisational affiliation of the review

Toronto Rehabilitation Institute
https://kite-uhn.com/

Review team members and their organisational affiliations

Dr Tatyana Mollayeva. Toronto Rehabilitation Institute
Ms Chi Kiu Lee. University of Toronto
Mrs Jessica Babineau. KITe Toronto Rehab UHN

Type and method of review

Diagnostic, Systematic review

Anticipated or actual start date

01 December 2021

Anticipated completion date

31 October 2022

Funding sources/sponsors

Tatyana Mollayeva is supported by the Canada Research Chair in Neurological Disorders and Brain Health

Grant number(s)
State the funder, grant or award number and the date of award
Canadian Institutes of Health Research

Conflicts of interest

None known

Language

English

Country

Canada

Stage of review

Review Ongoing

Subject index terms status

Subject indexing assigned by CRD

Subject index terms

Electroencephalography; Female; Humans; Male; Research Subjects; Sex Characteristics; Sleep

Date of registration in PROSPERO

10 May 2022

Date of first submission

22 April 2022

Details of any existing review of the same topic by the same authors

None exist

Stage of review at time of this submission

Stage	Started	Completed
Preliminary searches	Yes	No
Piloting of the study selection process	Yes	Yes
Formal screening of search results against eligibility criteria	Yes	No
Data extraction	No	No
Risk of bias (quality) assessment	No	No
Data analysis	No	No

The record owner confirms that the information they have supplied for this submission is accurate and complete and they understand that deliberate provision of inaccurate information or omission of data may be construed as scientific misconduct.

The record owner confirms that they will update the status of the review when it is completed and will add publication details in due course.

Versions

10 May 2022
10 May 2022

This information has been provided by the named contact for this review. CRD has accepted this information in good faith and registered the review in PROSPERO. The registrant confirms that the information supplied for this submission is accurate and complete. CRD bears no responsibility or liability for the content of this registration record, any associated files or external websites.
