# Supplementary material for: Sex differences in electrical activity of the brain during sleep: a systematic review of electroencephalographic findings across the human lifespan
Source: Biomed Eng Online. 2025 Mar 12;24:33. doi: 10.1186/s12938-025-01354-z (PMC11899717; doi:10.1186/s12938-025-01354-z)
Supplement: Supplementary file 7 — Supplementary material 7. [file 12938_2025_1354_MOESM7_ESM.pdf]

Supplementary Material S7

SEARCH STRATEGIES FOR ALL DATABASES

Database: MEDLINE(R) ALL <1946 to November 19, 2021>
Platform: Ovid
Date Searched: November 22, 2021

Search Strategy:
1 Sex Characteristics/ (58174)
2 Sex Factors/ (275399)
3 exp sex distribution/ (65992)
4 exp Gender Identity/ (21490)
5 (sex adj2 (differenc* or charact*)).tw,kf. (55140)
6 sex-related.tw,kf. (7404)
7 sex-based.tw,kf. (2179)
8 (sexes adj2 (differenc* or charact*)).tw,kf. (2283)
9 (gender adj2 (differenc* or charact*)).tw,kf. (45745)
10 gender-related.tw,kf. (5453)
11 gender-based.tw,kf. (4189)
12 ((male? or men or man or masculin* or boy?) and (female? or women or woman or feminin* or girl?)).ti,kf. (42291)
13 sex.ti,kf. (126413)
14 sexes.ti,kf. (1618)
15 gender*.ti,kf. (64337)
16 or/1-15 (566771)
17 exp Electroencephalography/ (172853)
18 Electroencephalogra*.tw,kf. (66069)
19 EEG.tw,kf. (85248)
20 Polysomnography/ (23193)
21 polysomnogr*.tw,kf. (21282)
22 PSG.tw,kf. (5671)
23 or/17-22 (230508)
24 exp Evoked Potentials/ (120978)
25 (Evoked adj2 (Potential? or respons*)).tw,kf. (67948)
26 (spectral adj2 (power or analys*)).tw,kf. (28081)
27 ((delta or alpha or beta or theta or gamma or sigma) adj2 (power or band? or range? or activit*)).tw,kf. (72805)
28 slow wave?.tw,kf. (12767)
29 non-rapid eye movement?.tw,kf. (2999)
30 nonrapid eye movement?.tw,kf. (595)
31 NREM.tw,kf. (4340)
32 rapid eye movement?.tw,kf. (11218)
33 REM.tw,kf. (15702)
34 exp Neuronal Plasticity/ (44310)
35 plasticit*.tw,kf. (102333)

36 spindle?.tw,kf. (49678)
37 synchronization*.tw,kf. (25341)
38 K-Complex*.tw,kf. (648)
39 or/24-38 (446613)
40 exp Sleep/ (87504)
41 exp Sleep Wake Disorders/ (98716)
42 sleep*.tw,kf. (204863)
43 40 or 41 or 42 (238803)
44 16 and (23 or 39) and 43 (1597)
45 44 not (exp animals/ not exp humans/) (1536)
46 limit 45 to english language (1434)

****

Database: Embase Classic+Embase <1947 to 2021 November 19>
Platform: Ovid
Date Searched: November 22, 2021

Search Strategy:
1 exp sex difference/ (432371)
2 "gender and sex"/ or exp gender identity/ (19885)
3 (sex adj2 (differenc* or charact*)).tw,kf. (73571)
4 (sex adj2 (differenc* or charact*)).tw,kf. (73571)
5 sex-related.tw,kf. (9109)
6 sex-based.tw,kf. (2819)
7 (sexes adj2 (differenc* or charact*)).tw,kf. (3052)
8 (gender adj2 (differenc* or charact*)).tw,kf. (66747)
9 gender-related.tw,kf. (7284)
10 gender-based.tw,kf. (5271)
11 ((male? or men or man or masculin* or boy?) and (female? or women or woman or feminin* or girl?)).ti,kf. (51306)
12 sex.ti,kf. (151001)
13 sexes.ti,kf. (1822)
14 gender*.ti,kf. (89695)
15 or/1-14 (650058)
16 exp electroencephalography/ (151197)
17 exp electroencephalogram/ (160108)
18 Electroencephalogra*.tw,kf. (81792)
19 EEG.tw,kf. (144052)
20 polysomnography/ (42397)
21 polysomnography device software/ (21)
22 polysomnogr*.tw,kf. (38962)
23 PSG.tw,kf. (13441)
24 or/16-23 (354528)

25 exp evoked response/ (80034)
26 (Evoked adj2 (Potential? or respons*)).tw,kf. (89287)
27 (spectral adj2 (power or analys*)).tw,kf. (37519)
28 ((delta or alpha or beta or theta or gamma or sigma) adj2 (power or band? or range? or activit*)).tw,kf. (86496)
29 slow wave?.tw,kf. (20344)
30 nonREM sleep/ (8870)
31 non-rapid eye movement?.tw,kf. (3835)
32 NREM.tw,kf. (7618)
33 REM sleep/ (30140)
34 sleep parameters/ (5135)
35 rapid eye movement?.tw,kf. (14620)
36 REM.tw,kf. (27088)
37 plasticity/ (20852)
38 exp cell plasticity/ (81152)
39 plasticit*.tw,kf. (127665)
40 spindle?.tw,kf. (68545)
41 synchronization*.tw,kf. (30075)
42 K-Complex*.tw,kf. (1002)
43 or/25-42 (544513)
44 exp sleep/ (274434)
45 exp sleep disorder/ (269994)
46 exp sleep stage/ (44456)
47 sleep*.tw,kf. (318771)
48 or/44-47 (478598)
49 15 and (24 or 43) and 48 (2741)
50 49 not ((exp animal/ or exp invertebrate/ or nonhuman/ or animal experiment/ or animal tissue/ or animal model/ or exp plant/ or exp fungus/) not (exp human/ or human tissue/)) (2612)
51 limit 50 to english language (2492)
52 limit 51 to conference abstracts (506)
53 51 not 52 (1986)
54 53 not medline.cr. (1817)

****

Database: APA PsycInfo <1806 to November Week 3 2021>
Platform: Ovid
Date Searched: November 22, 2021

Search Strategy:
1 exp human sex differences/ (118214)
2 sex roles/ (18478)
3 exp gender identity/ (41425)

4 (sex adj2 (differenc* or charact*)).ti,ab,id. (39289)
5 sex-related.ti,ab,id. (2283)
6 sex-based.ti,ab,id. (631)
7 (sexes adj2 (differenc* or charact*)).ti,ab,id. (504)
8 (gender adj2 (differenc* or charact*)).ti,ab,id. (52262)
9 gender-related.ti,ab,id. (3306)
10 gender-based.ti,ab,id. (3448)
11 ((male? or men or man or masculin* or boy?) and (female? or women or woman or feminin* or girl?)).ti,id. (55445)
12 sex.ti,id. (97159)
13 sexes.ti,id. (676)
14 gender*.ti,id. (94617)
15 or/1-14 (273226)
16 exp electroencephalography/ (32263)
17 Electroencephalogra*.ti,ab,id. (23958)
18 EEG.ti,ab,id. (41906)
19 polysomnography/ (1960)
20 polysomnogr*.ti,ab,id. (5694)
21 PSG.ti,ab,id. (1247)
22 or/16-21 (58309)
23 exp evoked potentials/ (38751)
24 (Evoked adj2 (Potential? or respons*)).ti,ab,id. (21549)
25 (spectral adj2 (power or analys*)).ti,ab,id. (3663)
26 ((delta or alpha or beta or theta or gamma or sigma) adj2 (power or band? or range? or activit*)).ti,ab,id. (15088)
27 slow wave?.ti,ab,id. (5690)
28 non-rapid eye movement?.ti,ab,id. (1131)
29 nonrapid eye movement?.ti,ab,id. (484)
30 NREM.ti,ab,id. (2521)
31 nrem sleep/ (1972)
32 rapid eye movement?.ti,ab,id. (4507)
33 REM.ti,ab,id. (9157)
34 rem sleep/ (4599)
35 exp neural plasticity/ (19742)
36 plasticit*.ti,ab,id. (34320)
37 rapid eye movement/ (1394)
38 spindle?.ti,ab,id. (2041)
39 synchronization*.ti,ab,id. (7259)
40 K-Complex*.ti,ab,id. (206)
41 or/23-40 (117726)
42 exp sleep/ (38187)
43 exp sleep wake disorders/ (21165)
44 sleep wake cycle/ (4445)
45 sleep*.ti,ab,id. (84577)

46 or/42-45 (93111)
47 15 and (22 or 41) and 46 (361)
48 limit 47 to animal (40)
49 limit 48 to human (4)
50 47 not (48 not 49) (325)
51 limit 50 to english language (311)
52 limit 51 to ("0200 book" or "0240 authored book" or "0280 edited book" or "0300 encyclopedia" or "0400 dissertation abstract") (23)
53 51 not 52 (288)

****

Database: Scopus
Platform: Elsevier
Date Searched: November 22, 2021

Search Strategy:
(TITLE-ABS-KEY(sex W/2 (differenc* OR charact*)) OR TITLE-ABS-KEY("sex-related" OR "sex-based" OR "gender-related" OR "gender-based") OR TITLE-ABS-KEY(sexes W/2 (differenc* or charact*)) OR TITLE-ABS-KEY(gender W/2 (differenc* or charact*)) OR TITLE((male OR males OR men OR man OR masculin* OR boy OR boys) AND (female OR females OR women OR woman OR feminin* OR girl OR girls)) OR KEY((male OR males OR men OR man OR masculin* OR boy OR boys) AND (female OR females OR women OR woman OR feminin* OR girl OR girls)) OR TITLE(sex OR sexes OR gender*) OR KEY(sex OR sexes OR gender*)) AND TITLE-ABS-KEY(sleep*) AND TITLE-ABS-KEY(EEG OR Electroencephalogra* OR PSG OR polysomnogr* OR "slow wave" OR "non-rapid eye movement" OR NREM OR "rapid eye movement" OR REM OR plasticit* OR splindle* or synchronization* OR "k-complex" OR (Evoked W/2 (Potential* OR respons*)) OR (spectral W/2 (power OR analys*)) OR ((delta or alpha or beta or theta or gamma or sigma) W/2 (power or band* or range* or activit*))) AND NOT INDEX(medline or embase) AND (LIMIT-TO (LANGUAGE,"English")) AND (LIMIT-TO (SRCTYPE,"j"))
Results: 189 Results

****

Database: Dissertations and These Global
Platform: Proquest
Date Searched: November 22, 2021

Search Strategy: (NOFT sex W/2 (differenc* OR charact*) OR NOFT "sex-related" OR NOFT "sex-based" OR NOFT "gender-related" OR NOFT "gender-based" OR NOFT sexes W/2 (differenc* or charact*) OR NOFT gender W/2 (differenc* or charact*) OR ti((male OR males OR men OR man OR masculin* OR boy OR boys) AND (female OR females OR

women OR woman OR feminin* OR girl OR girls)) OR ti(sex OR sexes OR gender*)) AND (NOFT(sleep*)) AND (NOFT(EEG OR Electroencephalogra* OR PSG OR polysomnogr* OR "slow wave" OR "non-rapid eye movement" OR NREM OR "rapid eye movement" OR REM OR plasticit* OR splindle* or synchronization* OR "k-complex" OR (Evoked W/2 (Potential* OR respons*)) OR (spectral W/2 (power OR analys*)) OR ((delta or alpha or beta or theta or gamma or sigma) W/2 (power or band* or range* or activit*))))
Results: 10 results

Updated Searches

Search Strategies for All Databases

Database: Ovid MEDLINE(R) ALL <1946 to December 12, 2024>

Platform: Ovid

Date Searched: December 13, 2024

#	Query	Results from 13 Dec 2024
1	Sex Characteristics/	63,654
2	Sex Factors/	284,958
3	exp sex distribution/	67,067
4	exp Gender Identity/	28,497
5	(sex adj2 (differenc* or charact*)).tw,kf.	72,284
6	sex-related.tw,kf.	9,413
7	sex-based.tw,kf.	3,736
8	(sexes adj2 (differenc* or charact*)).tw,kf.	2,938
9	(gender adj2 (differenc* or charact*)).tw,kf.	55,618
10	gender-related.tw,kf.	6,555
11	gender-based.tw,kf.	6,542
12	((male? or men or man or masculin* or boy?) and (female? or women or woman or feminin* or girl?)).ti,kf.	48,982
13	sex.ti,kf.	155,698
14	sexes.ti,kf.	1,952
15	gender*.ti,kf.	86,728
16	or/1-15	638,283
17	exp Electroencephalography/	177,866
18	Electroencephalogra*.tw,kf.	80,975
19	EEG.tw,kf.	103,295
20	Polysomnography/	26,281
21	polysomnogr*.tw,kf.	25,699
22	PSG.tw,kf.	7,300
23	or/17-22	251,001
24	exp Evoked Potentials/	127,846
25	(Evoked adj2 (Potential? or respons*)).tw,kf.	73,940
26	(spectral adj2 (power or analys*)).tw,kf.	32,546
27	((delta or alpha or beta or theta or gamma or sigma) adj2 (power or band? or range? or activit*)).tw,kf.	84,228
28	slow wave?.tw,kf.	14,030
29	non-rapid eye movement?.tw,kf.	3,689
30	nonrapid eye movement?.tw,kf.	693
31	NREM.tw,kf.	5,242
32	rapid eye movement?.tw,kf.	13,331

33	REM.tw,kf.	18,314
34	exp Neuronal Plasticity/	48,366
35	plasticit*.tw,kf.	125,069
36	spindle?.tw,kf.	56,198
37	synchronization*.tw,kf.	30,250
38	K-Complex*.tw,kf.	716
39	or/24-38	508,515
40	exp Sleep/	105,236
41	exp Sleep Wake Disorders/	116,891
42	sleep*.tw,kf.	259,284
43	40 or 41 or 42	295,481
44	16 and (23 or 39) and 43	1,805
45	44 not (exp animals/ not exp humans/)	1,727
46	limit 45 to english language	1,622

Database: Embase Classic+Embase <1947 to 2024 December 12>
Platform: Ovid
Date Searched: December 13, 2024

#	Query	Results from 13 Dec 2024
1	exp sex difference/	498,321
2	"gender and sex"/ or exp gender identity/	26,033
3	(sex adj2 (differenc* or charact*)).tw,kf.	97,175
4	(sex adj2 (differenc* or charact*)).tw,kf.	97,175
5	sex-related.tw,kf.	11,741
6	sex-based.tw,kf.	4,941
7	(sexes adj2 (differenc* or charact*)).tw,kf.	3,941
8	(gender adj2 (differenc* or charact*)).tw,kf.	80,295
9	gender-related.tw,kf.	8,721
10	gender-based.tw,kf.	7,949
11	((male? or men or man or masculin* or boy?) and (female? or women or woman or feminin* or girl?)).ti,kf.	60,037
12	sex.ti,kf.	187,331
13	sexes.ti,kf.	2,216
14	gender*.ti,kf.	115,468
15	or/1-14	772,133
16	exp electroencephalography/	184,959
17	exp electroencephalogram/	195,952
18	Electroencephalogra*.tw,kf.	101,564
19	EEG.tw,kf.	171,628

20	polysomnography/	53,162
21	polysomnography device software/	131
22	polysomnogr*.tw,kf.	47,052
23	PSG.tw,kf.	16,811
24	or/16-23	424,704
25	exp evoked response/	94,743
26	(Evoked adj2 (Potential? or respons*)).tw,kf.	98,141
27	(spectral adj2 (power or analys*)).tw,kf.	43,135
28	((delta or alpha or beta or theta or gamma or sigma) adj2 (power or band? or range? or activit*)).tw,kf.	101,878
29	slow wave?.tw,kf.	22,547
30	nonREM sleep/	11,791
31	non-rapid eye movement?.tw,kf.	4,799
32	NREM.tw,kf.	9,351
33	REM sleep/	35,445
34	sleep parameters/	6,947
35	rapid eye movement?.tw,kf.	17,625
36	REM.tw,kf.	31,916
37	plasticity/	23,095
38	exp cell plasticity/	100,451
39	plasticit*.tw,kf.	155,622
40	spindle?.tw,kf.	78,443
41	synchronization*.tw,kf.	36,084
42	K-Complex*.tw,kf.	1,120
43	or/25-42	640,414
44	exp sleep/	311,445
45	exp sleep disorder/	280,467
46	exp sleep stage/	53,966
47	sleep*.tw,kf.	401,179
48	or/44-47	573,894
49	15 and (24 or 43) and 48	3,402
50	49 not ((exp animal/ or exp invertebrate/ or nonhuman/ or animal experiment/ or animal tissue/ or animal model/ or exp plant/ or exp fungus/) not (exp human/ or human tissue/))	3,205
51	limit 50 to english language	3,077
52	limit 51 to conference abstracts	643
53	51 not 52	2,434
54	53 not medline.cr.	2,230

Database: APA PsycInfo <1806 to December 2024 Week 1>
Platform: Ovid

Date Searched: December 13, 2024

#	Query	Results from 13 Dec 2024
1	exp human sex differences/	131,323
2	sex roles/	19,893
3	exp gender identity/	52,614
4	(sex adj2 (differenc* or charact*)).ti,ab,id.	43,681
5	sex-related.ti,ab,id.	2,644
6	sex-based.ti,ab,id.	836
7	(sexes adj2 (differenc* or charact*)).ti,ab,id.	583
8	(gender adj2 (differenc* or charact*)).ti,ab,id.	58,063
9	gender-related.ti,ab,id.	3,774
10	gender-based.ti,ab,id.	4,791
11	((male? or men or man or masculin* or boy?) and (female? or women or woman or feminin* or girl?)).ti,id.	58,098
12	sex.ti,id.	105,681
13	sexes.ti,id.	715
14	gender*.ti,id.	111,669
15	or/1-14	311,163
16	exp electroencephalography/	39,382
17	Electroencephalogra*.ti,ab,id.	29,102
18	EEG.ti,ab,id.	48,475
19	polysomnography/	2,450
20	polysomnogr*.ti,ab,id.	6,710
21	PSG.ti,ab,id.	1,524
22	or/16-21	68,075
23	exp evoked potentials/	43,354
24	(Evoked adj2 (Potential? or respons*)).ti,ab,id.	23,041
25	(spectral adj2 (power or analys*)).ti,ab,id.	4,212
26	((delta or alpha or beta or theta or gamma or sigma) adj2 (power or band? or range? or activit*)).ti,ab,id.	18,134
27	slow wave?.ti,ab,id.	6,158
28	non-rapid eye movement?.ti,ab,id.	1,402
29	nonrapid eye movement?.ti,ab,id.	518
30	NREM.ti,ab,id.	2,872
31	nrem sleep/	2,250
32	rapid eye movement?.ti,ab,id.	5,318
33	REM.ti,ab,id.	10,094
34	rem sleep/	5,156
35	exp neural plasticity/	21,519
36	plasticit*.ti,ab,id.	38,193

37	rapid eye movement/	1,463
38	spindle?.ti,ab,id.	2,315
39	synchronization*.ti,ab,id.	8,373
40	K-Complex*.ti,ab,id.	217
41	or/23-40	132,097
42	exp sleep/	47,571
43	exp sleep wake disorders/	26,669
44	sleep wake cycle/	5,278
45	sleep*.ti,ab,id.	100,340
46	or/42-45	110,265
47	15 and (22 or 41) and 46	437
48	limit 47 to animal	53
49	limit 48 to human	5
50	47 not (48 not 49)	389
51	limit 50 to english language	374
52	limit 51 to ("0200 book" or "0240 authored book" or "0280 edited book" or "0300 encyclopedia" or "0400 dissertation abstract")	24
53	51 not 52	350

Database: Scopus
Platform: Elsevier
Date Searched: December 13, 2024

(TITLE-ABS-KEY(sex W/2 (differenc* OR charact*)) OR TITLE-ABS-KEY("sex-related" OR "sex-based" OR "gender-related" OR "gender-based") OR TITLE-ABS-KEY(sexes W/2 (differenc* or charact*)) OR TITLE-ABS-KEY(gender W/2 (differenc* or charact*)) OR TITLE((male OR males OR men OR man OR masculin* OR boy OR boys) AND (female OR females OR women OR woman OR feminin* OR girl OR girls)) OR KEY((male OR males OR men OR man OR masculin* OR boy OR boys) AND (female OR females OR women OR woman OR feminin* OR girl OR girls)) OR TITLE(sex OR sexes OR gender*) OR KEY(sex OR sexes OR gender*)) AND TITLE-ABS-KEY(sleep*) AND TITLE-ABS-KEY(EEG OR Electroencephalogra* OR PSG OR polysomnogr* OR "slow wave" OR "non-rapid eye movement" OR NREM OR "rapid eye movement" OR REM OR plasticit* OR splindle* or synchronization* OR "kcomplex" OR (Evoked W/2 (Potential* OR respons*)) OR (spectral W/2 (power OR analys*)) OR ((delta or alpha or beta or theta or gamma or sigma) W/2 (power or band* or range* or activit*))) AND NOT INDEX(medline or embase) AND (LIMIT-TO (LANGUAGE,"English")) AND (LIMIT-TO (SRCTYPE,"j"))
Results: 138 Results

Database: Dissertations and These Global
Platform: Proquest

Date Searched: December 13, 2024

(NOFT sex W/2 (differenc* OR charact*) OR NOFT "sex-related" OR NOFT "sex-based" OR NOFT "gender-related" OR NOFT "gender-based" OR NOFT sexes W/2 (differenc* or charact*) OR NOFT gender W/2 (differenc* or charact*) OR ti((male OR males OR men OR man OR masculin* OR boy OR boys) AND (female OR females OR women OR woman OR feminin* OR girl OR girls)) OR ti(sex OR sexes OR gender*)) AND (NOFT(sleep*)) AND (NOFT(EEG OR Electroencephalogra* OR PSG OR polysomnogr* OR "slow wave" OR "non-rapid eye movement" OR NREM OR "rapid eye movement" OR REM OR plasticit* OR splindle* or synchronization* OR "k-complex" OR (Evoked W/2 (Potential* OR respons*)) OR (spectral W/2 (power OR analys*)) OR ((delta or alpha or beta or theta or gamma or sigma) W/2 (power or band* or range* or activit*))))
Results: 11 results
